# Diversity and Divergence of Dinoflagellate Histone Proteins

**DOI:** 10.1534/g3.115.023275

**Published:** 2015-12-08

**Authors:** Georgi K. Marinov, Michael Lynch

**Affiliations:** Department of Biology, Indiana University, Bloomington, Indiana 47405

**Keywords:** chromatin, dinoflagellates, histone code, histones, transcription

## Abstract

Histone proteins and the nucleosomal organization of chromatin are near-universal eukaroytic features, with the exception of dinoflagellates. Previous studies have suggested that histones do not play a major role in the packaging of dinoflagellate genomes, although several genomic and transcriptomic surveys have detected a full set of core histone genes. Here, transcriptomic and genomic sequence data from multiple dinoflagellate lineages are analyzed, and the diversity of histone proteins and their variants characterized, with particular focus on their potential post-translational modifications and the conservation of the histone code. In addition, the set of putative epigenetic mark readers and writers, chromatin remodelers and histone chaperones are examined. Dinoflagellates clearly express the most derived set of histones among all autonomous eukaryote nuclei, consistent with a combination of relaxation of sequence constraints imposed by the histone code and the presence of numerous specialized histone variants. The histone code itself appears to have diverged significantly in some of its components, yet others are conserved, implying conservation of the associated biochemical processes. Specifically, and with major implications for the function of histones in dinoflagellates, the results presented here strongly suggest that transcription through nucleosomal arrays happens in dinoflagellates. Finally, the plausible roles of histones in dinoflagellate nuclei are discussed.

A core feature of eukaryotic genome biology is the nucleosomal organization of chromatin. Nucleosomes consist of a histone octamer containing two copies of each of the four core histone proteins H2A, H2B, H3, and H4, wrapped around ∼147 bp of DNA (Luger *et al.* 1997). In addition, the linker histone H1 binds to the nucleosome and the linker DNA between individual nucleosomes.

The major exception from this almost universal organization is the dinoflagellate lineage. Dinoflagellates exhibit numerous highly unusual features, such as the organization of their mitochondrial (Waller and Jackson 2009) and plastid (Zhang *et al.* 1999; Barbrook and Howe 2000) genomes, but their nuclei are particularly striking (Rizzo 2003). Dinoflagellate chromatin does not exhibit a banding pattern upon nuclease digestion, it contains little acid-soluble protein (the ratio of basic proteins to DNA is ∼10% compared to the typical 1:1; Rizzo and Nooden 1972; Herzog and Soyer 1981), and chromosomes exist in a permanently condensed liquid crystalline state (Rill *et al.* 1989). Histone proteins are not readily detected in dinoflagellates, and until quite recently they were thought to be completely absent. So unusual is dinoflagellate chromatin that at one time dinoflagellates were suggested to be “mesokaryotes”, *i.e.*, intermediate between prokaryotes and eukaryotes (Dodge 1965). We now know that dinoflagellates firmly belong to the alveolates, together with apicomplexans and ciliates, and that the loss of nucleosomes is a derived feature. But the mystery of dinoflagellate chromatin remains largely unresolved.

Several reports have identified histone-like proteins in dinoflagellates (Chan *et al.* 2006; Chan and Wong 2007; Wargo and Rizzo 2000; Chudnovsky *et al.* 2002; Sala-Rovira *et al.* 1991; Wong *et al.* 2003; Rizzo and Burghardt 1982). More recently, it was found that dinoflagellates express virus-derived nucleoproteins, completely unrelated to histones (dinoflagellate viral nucleoproteins; DVNPs), which seem to substitute for histones as far as the packaging of DNA is concerned (Gornik *et al.* 2012). However, multiple reports have also identified histone genes and low levels of histone proteins in several species. These include studies of transcriptomes from *Lingulodinium* (Roy and Morse 2012), *Symbiodinium* (Bayer *et al.* 2012), and *Alexandrium catenella* (Zhang *et al.* 2014), the draft genome sequence of *S. minutum* (Shoguchi *et al.* 2013), and environmental transcriptomes (Lin *et al.* 2010).

These observations suggest that histones do play some role in dinoflagellate biology, but its precise nature remains unclear. A somewhat underappreciated fact is that the loss of nucleosomes has far more profound consequences than the mere packaging of DNA, as the post-translational modifications (PTMs) of histone proteins and the “histone code” they constitute (Jenuwein and Allis 2001) play a key role in most aspects of chromatin biology. These modifications happen primarily (but not only) in the N-terminal tails of histones and serve as platforms for the recruitment of specific PTM “reader” domain-containing proteins (Kouzarides 2007). Hundreds of histone modifications have been identified, densely covering histone tails (Huang *et al.* 2014), which is one explanation for the extreme conservation of their sequence across very deeply diverging lineages of eukaryotes (Waterborg 2012; Postberg *et al.* 2010; Feng and Jacobsen 2011).

In the light of the deep conservation and fundamental importance of the histone code, it is of significant interest to know the extent to which it is conserved in dinoflagellates given that histones are present but are not the major constituent of chromatin in these organisms. Such insights can shed light on the functional roles of histone proteins in dinoflagellate biology. In this study, these issues are addressed by carrying out a detailed survey of the sequence of histone proteins, as well as the presence or absence of chromatin mark writers, readers, and erasers in available transcriptomic and genomic data from a large number of dinoflagellate species.

## Materials and Methods

### Genomic and transcriptomic sequence data

Marine Microbial Eukaryote Transcriptome Sequencing Project (MMETSP) transcriptome datasets and assemblies were downloaded on June 19, 2014. *Glenodinium foliaceum* (Stein 1883) and *Kryptoperidinium foliaceum* (Lindemann 1924) are listed separately following the submission labels, even though they are considered synonymous (Gómez 2005). Low-quality transcriptome assemblies, featuring very low numbers of assembled transcripts, were removed. A full list of the samples used is provided in Supporting Information, Table S1. In addition, genome assemblies and annotations for *Perkinsus marinus* (accession number GCF_000006405.1) and *S. minutum* were used. Additional genome assemblies and annotations were downloaded from the NCBI (*Thecamonas trahens*: GCA_000142905.1, *Acanthamoeba castellanii*: GCF_000313135.1, *Monosiga brevicollis*: GCF_000002865.2, *Paramecium caudatum*: GCA_000715435.1, *Capsaspora owczarzaki*: GCF_000151315.1, *Trichomonas vaginalis*: GCF_000002825.2, *Ectocarpus siliculosus*: GCA_000310025.1) or from the EnsemblProtists (*Plasmodium falciparum*, *Toxoplasma gondii*, *Entamoeba histolytica*, *Chlamydomonas reinhardtii*, *Micromonas pusilla*, *Tetrahymena thermophila*, *Guillardia theta*, *Emiliania huxleyi*, *Naegleria gruberi*, *Dictyostelium discoideum*, *Phytophthora infestans*, *Cyanidioschyzon merolae*) and EnsemblFungi (*Saccharomyces cerevisiae*, *Schizosaccharomyces pombe*) databases.

### Sequence analysis

Histone proteins were identified using a combination of BLASTP searches (using histone sequences from *Homo sapiens*, *S. cerevisiae*, *Drosophila melanogaster*, *Arabidopsis thaliana*, and *T. thermophila* as queries, and an e-value of $10^{-10}$ as cutoff) and HMMER3.0 (Eddy 2011) scans against the Pfam 27.0 database (Finn *et al.* 2014) (scanning for histone fold and linker histone domains).

For most of the analyses presented, incomplete hits (*i.e.*, partial sequences without both a start and a stop codon) were removed. For the analysis of H3 histone tails, proteins with complete N-termini but incomplete C-termini were included; in addition, H3 sequences were manually examined and in cases where additional amino acid residues were present in front of an otherwise clearly conventional histone H3 tail, such residues were removed (their most likely origin is the presence of an earlier start codon in the transcript assembly and the most parsimonious explanation is that they are not part of the actual protein). Clear cases of misassembly (such as concatenated copies of histone proteins in the same sequence) were also removed.

Multiple sequence alignments were carried out using MUSCLE (Edgar 2004; version 3.8.31) and visualized with JalView (Waterhouse *et al.* 2009; version 2.8.2). Phylogenetic analysis was carried out using MEGA 6.0 (Tamura *et al.* 2013) (selection of best substitution model) and RAxML (Stamatakis 2014) (version 8.0.26; generation of maximum likelihood trees and bootstrap analysis).

### Gene expression quantification

Sequencing reads were first mapped (as 2 × 50 bp sequences) to the transcriptome assemblies using Bowtie (Langmead *et al.* 2009; version 1.0.1), with the following settings: -v 3 -a -t -X 1000. Transcript-level quantification was then carried out with eXpress (Roberts and Pachter 2013; version 1.5.1). Transcript per million (TPM) values were used for subsequent analysis.

## Results

### Histone proteins in dinoflagellates

**Figure 1:**
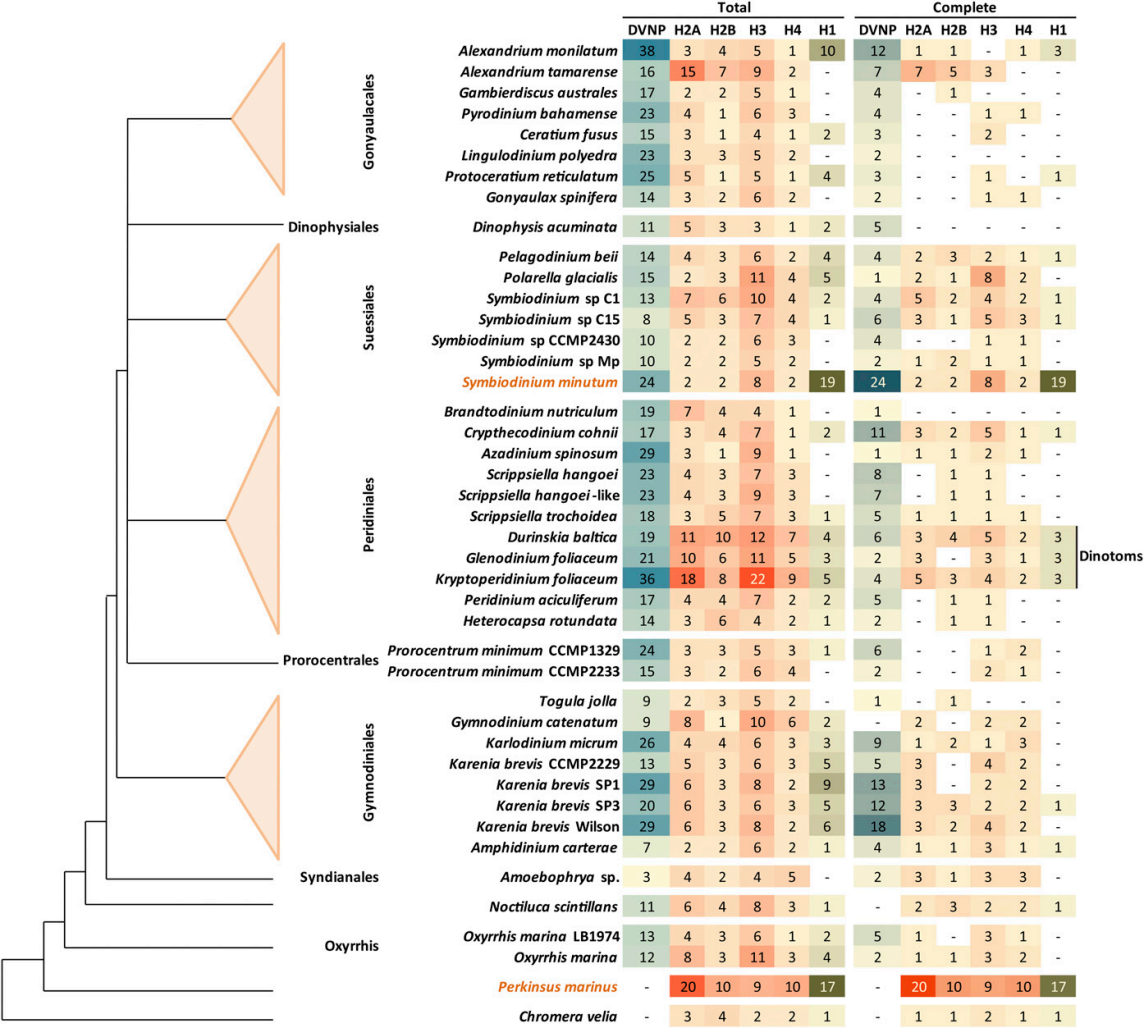
Detection of histones and DVNP proteins in dinoflagellate transcriptomic and genomic assemblies. “Total” refers to the number of unique proteins detected, while “complete” refers to the subset of full-length proteins (*i.e.*, assembled transcripts in which both start and stop codons are present). *Symbiodinium minutum* and *Perkinsus marinus* are colored differently as genome assemblies are available for these species, while only the transcriptomic space has been sampled for all others. The dinotoms are also highlighted separately as they contain an unreduced diatom endosymbiont, meaning that their transcriptomes contain transcripts from two different eukaryotic genomes, only one of which is a dinoflagellate. The cladogram was generated following previously published phylogenies (Orr *et al.* 2014).

Their often very large size and existing technological limitations have so far prevented the complete sequencing of dinoflagellate genomes, and little is known in detail about their sequence and organization. Fortunately, in recent years, transcriptomic data from a large number of dinoflagellates has become available, in particular through the efforts of the MMETSP (Keeling *et al.* 2014). MMETSP transcriptome assemblies and translations served as the main dataset for this study. In addition, a draft genomic sequence exists for *S. minutum* (Shoguchi *et al.* 2013) even though it is far from complete, and a genome assembly is available from the NCBI database for *P. marinus*, a member of the early branching, sister to dinoflagellates lineage, the perkinsids; annotated proteins from these genome assemblies were also included. Finally, an MMETSP transcriptome for *Chromera velia*, a photosynthetic relative of apicomplexans, was used as an outgroup/control. The species considered and their evolutionary relationships are shown in Figure 1, and the full list of datasets can be found in Table S1. In total, the analysis focused on 40 dinoflagellate species/isolates with transcriptome assemblies, two species with genome sequences, and the *C. velia* transcriptome.

A combination of hidden Markov model (HMM) scans and BLASTP searches against translated transcriptomes (see the *Materials and Methods* section and figure legends for more details) was used to identify histone proteins. A similar search for DVNPs was also carried out. Part of the reality of working with transcriptome assemblies is that proteins are not always completely assembled, which can confound certain analyses. For these reasons, hits were divided into complete and incomplete sequences, and putative misassemblies were filtered out (details in the *Materials and Methods* section).

Figure 1 shows the number of fully and incompletely assembled core histone proteins, putative linker histones, and DVNPs in all species studied. All four core histones were identified in all dinoflagellates, usually in multiple distinct variants, with histone H3 exhibiting a particularly great diversity. Putative linker histones were also identified in many species; the breadth of its phylogenetic distribution suggests that histone H1 is present in all dinoflagellates, with the failures to detect it being false negatives.

A unique representative of the complexity of dinoflagellate biology deserves a special mention. The largest number of histones were identified in *Durinskia baltica, Kryptoperidinium foliaceum, and Glenodinium foliaceum*. These species are known as “dinotoms” (Imanian *et al.* 2010), as they harbor a tertiary diatom endosymbiont (Dodge 1971; Tomas and Cox 1973; Figueroa *et al.* 2009), which has not been reduced and retains a large genome. This means that they have both a dinoflagellate nucleus and a diatom one, the latter with conventional nucleosomal organization. Thus, all results that follow should be interpreted with this caveat in mind in the case of dinotoms.

Another potentially confounding factor concerns the purity of the samples studied, many of which were not axenic (Table S1). In some cases, the presence of other eukaryotes is necessary to maintain dinoflagellate cultures. For example, *Dinophysis* contains kleptoplastids of cryptophyte origin (Schnepf and Elbrächter 1988; Nagai *et al.* 2008), which apparently do not contain a nucleomorph (Lucas and Vesk 1990), and are extracted from the ciliate *Myrionecta rubra* (=*Mesodinium rubra*) (Takishita *et al.* 2002), which in turn acquires them from cryptophytes (Johnson *et al.* 2006); *Dinophysis* is grown together with *Myrionecta*. The other such example involves *Oxyrrhis marina*, which is heterotrophic and is often grown with other eukaryotes as prey (Lowe *et al.* 2011), in this case the diatom *Phaeodactylum tricornutum*.

Thus, there is a possibility that histones and other proteins identified in transcriptome assemblies do not belong to the listed species. Dinoflagellate transcripts are subject to *trans*-splicing, thus in principle, the presence or absence of splice leaders can be used to identify transcripts of dinoflagellate origin. However, this is best done by using the splice leader to positively select transcripts during library construction (Gavelis *et al.* 2015), while conventional RNA-seq is highly vulnerable to underrepresentation of the extreme 5′ ends of transcripts, meaning that the absence of splice leaders is not a reliable marker for the nondinoflagellate origin of transcripts.

Despite these caveats, two observations argue against contamination being a major issue with the analysis presented here: first, histones are found in both axenic and nonaxenic cultures (Table S1 and Figure 1), and second, as will become clear below, the properties of putative dinoflagellate histones are quite unique, making them unlikely to come from other eukaryotes.

### Expression levels of dinoflagellate histones

**Figure 2:**
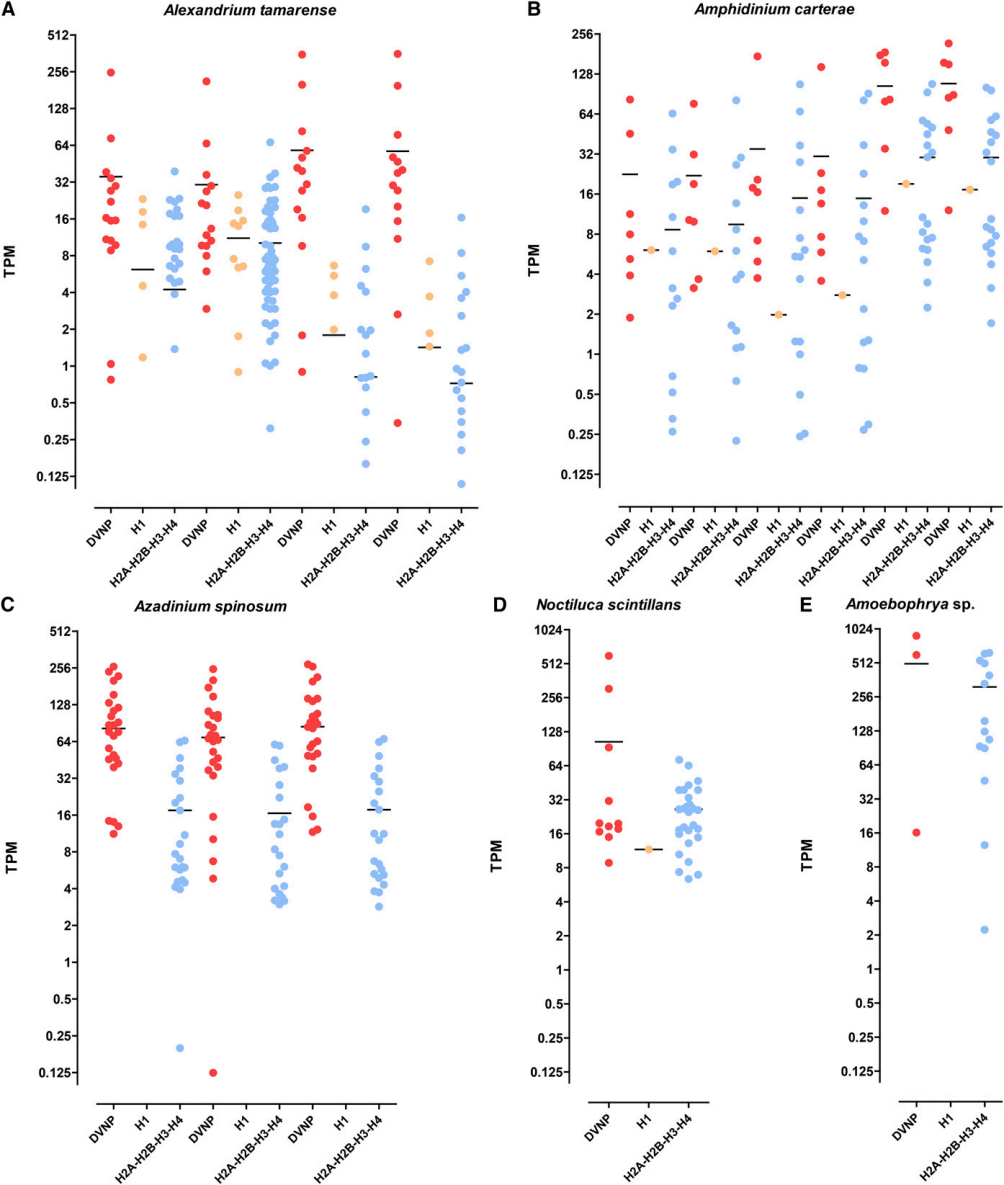
Expression levels of DVNP, linker histone, and histone genes in dinoflagellates in TPM (transcripts per million). (A) *Alexandrium tamarense*; from left to right: SRR1296765, SRR1296766, SRR1300221, SRR1300222; (B) *Amphidinium carterae*; from left to right: SRR1294391, SRR1294392, SRR1294393, SRR1294394, SRR1296757, SRR1296758; (C) *Azadinium spinosum*; from left to right: SRR1300306, SRR1300307, SRR1300308; (D) *Noctiluca scintillans*: SRR1296929; (E) *Amoebophrya* sp.: SRR1296703. See Figure S2, Figure S3, Figure S4, Figure S5, Figure S6, and Figure S7 for data for other species.

The expression levels of histones can provide additional information about their functional significance in dinoflagellates. To this end, transcriptomes were quantified by aligning the raw reads back to the assemblies and carrying out transcript-level quantification using eXpress (Roberts and Pachter 2013). Figure 2 and Figure S2, Figure S3, Figure S4, Figure S5, Figure S6, and Figure S7 show the distribution of expression values for core histones, linker histones, and DVNPs. In all nondinotom species, DVNPs are significantly more highly expressed than histones, although considerable variations in the relative levels are observed.

While these results should be treated with caution as it is well known that replication-dependent histone mRNAs are not polyadenylated in other eukaryotes (Marzluff 2005), while the datasets studied were generated after polyA-selection, these observations are consistent with histones being present at relatively low levels and DVNPs being the main packaging proteins in dinoflagellates.

### Properties of dinoflagellate histones

**Figure 3:**
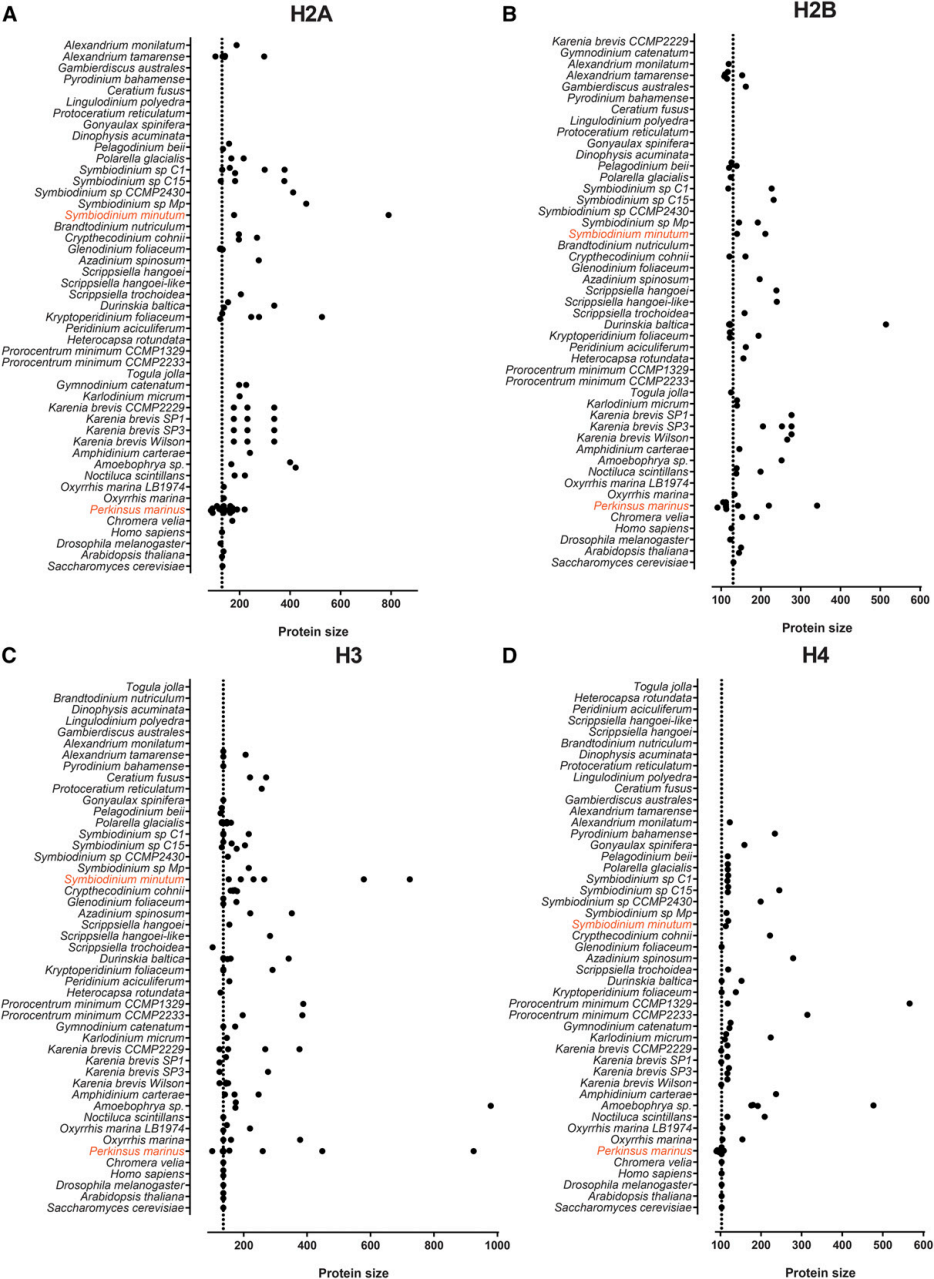
Distribution of histone protein lengths in dinoflagellates. (A) Histone H2A; (B) Histone H2B; (C) Histone H3; (D) Histone H4. Values for *Homo sapiens*, *Drosophila melanogaster*, *Saccharomyces cerevisiae*, and *Arabidopsis thaliana* core histones are shown at the bottom of each panel for comparison.

Previous reports in individual species have noted the increased length of dinoflagellate histones compared to conventional core histones. Figure 3 shows the lengths of all complete H2A, H2B, H3, and H4 proteins identified in this study. Dinoflagellate histones are indeed frequently elongated. However, this is not a universal feature, as many normal-length histones are also observed, and often the same species expresses both long and short histone variants.

The elongation of dinoflagellate histone proteins is not due to the presence of additional protein domains as only core histone domains are readily identifiable (see example in Figure S1), with only two exceptions: a TRAM-LAG1-CLN8 domain in a *K. foliaceum* H2A, and a NAD(P)-binding domain in an *A. tamarense* H2A; their functional significance is currently unclear.

**Figure 4:**
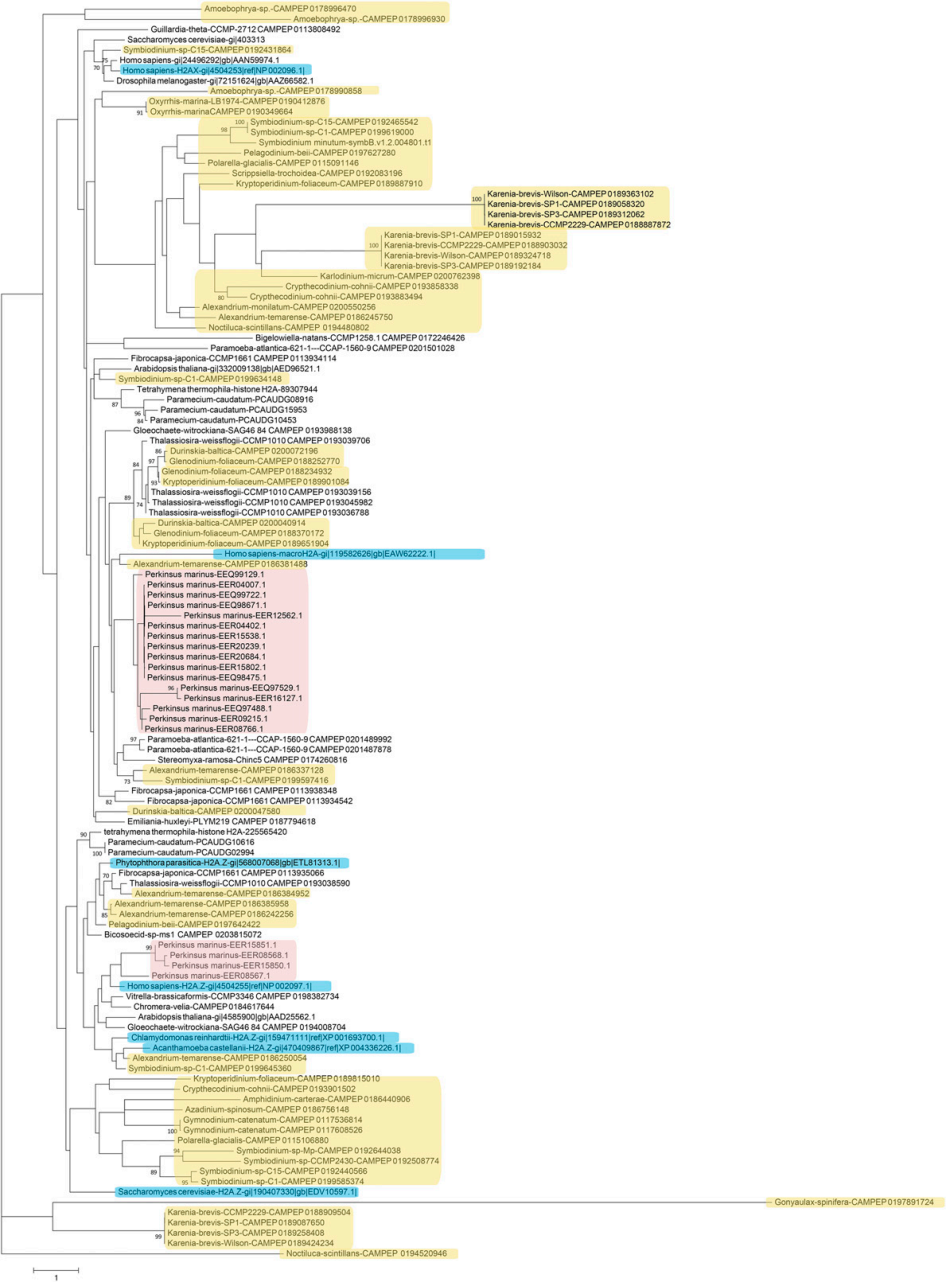
Maximum likelihood phylogenetic tree of H2A protein sequences in dinoflagellates. The tree was generated using RAxML (version 8.0.26) under the LG+G model and with 100 bootstrap replicates. Additional H2A sequences from genome and MMETSP assemblies of other protists were included (the ciliates *Paramecium caudatum* and *Tetrahymena thermophila*, the cryptophyte *Guillardia theta*, the diatom *Thalassiosira weissflogii*, the discosean amoeba *Paramoeba atlantica*, another amoebozoan, *Stereomyxa ramosa*, the bicosoecid *Bicosoecid* sp., the chlorarachniophyte *Bigelowiella natans*, the two chromerids *Chromera velia* and *Vitrella brassicaformis*, the glaucophyte *Gloeochaete witrockiana*, the haptophyte *Emiliania huxleyi*, the raphidophyte *Fibrocapsa japonica*) as well as sequences from human, yeast, flies, and *Arabidopsis*. The H2A variants H2A.Z (from multiple eukaryotes), and H2A.X and macroH2A (from *Homo sapiens*) were also included, and are highlighted in blue. Dinoflagellate sequences are marked in yellow while perkinsid proteins are colored in pink.

**Figure 5:**
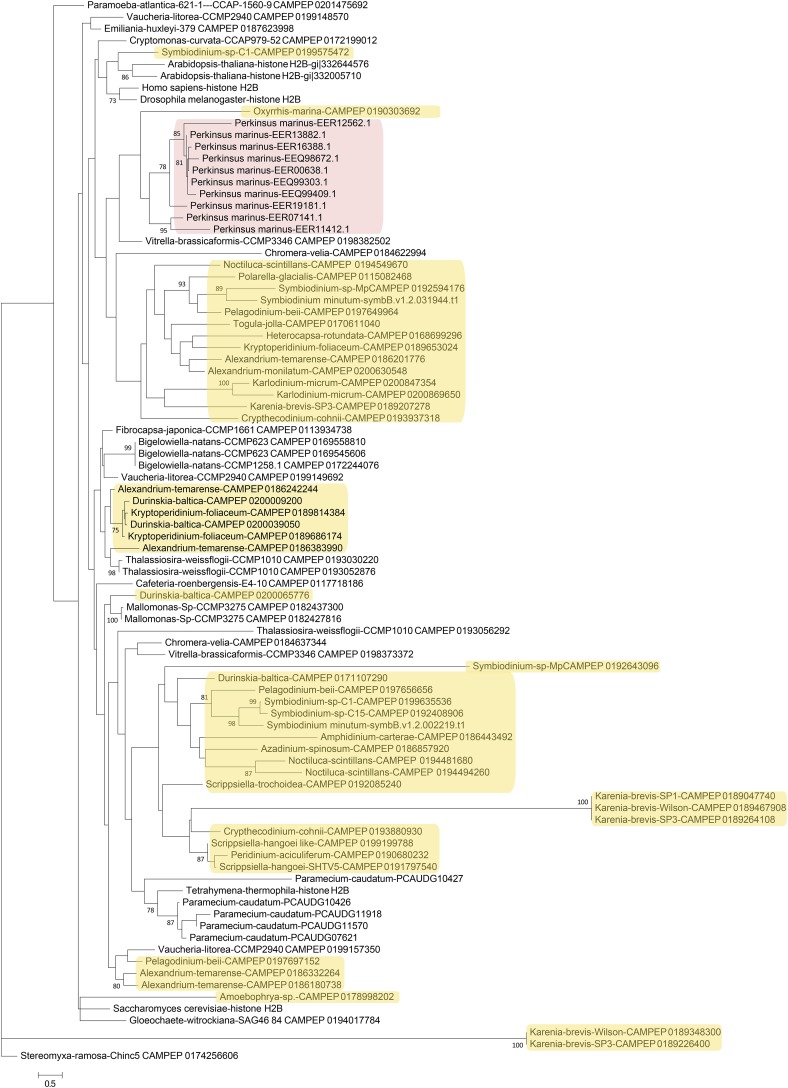
Maximum likelihood phylogenetic tree of H2B protein sequences in dinoflagellates and perkinsids. The tree was generated using RAxML (version 8.0.26) under the LG+G model and with 100 bootstrap replicates. Additional sequences from genome sequences and MMETSP assemblies of other protists were included (the two chromerids *Chromera velia* and *Vitrella brassicaformis*, the ciliates *Paramecium caudatum* and *Tetrahymena thermophila*, the discosean amoeba *Paramoeba atlantica*, the bicosoecid *Cafeteria roenbergensis*, the chlorarachniophyte *Bigelowiella natans*, the chrysophyte *Mallomonas*, the cryptophyte *Cryptomonas curvata*, the diatom *Thalassiosira weissflogii*, the glaucophyte *Gloeochaete witrockiana*, the haptophyte *Emiliania huxleyi*, the raphidophyte *Fibrocapsa japonica*, the xanthopyte *Vaucheria litorea*) as well as sequences from human, yeast, flies, and *Arabidopsis*. Dinoflagellate sequences are highlighted in yellow while perkinsid proteins are marked in pink.

**Figure 6:**
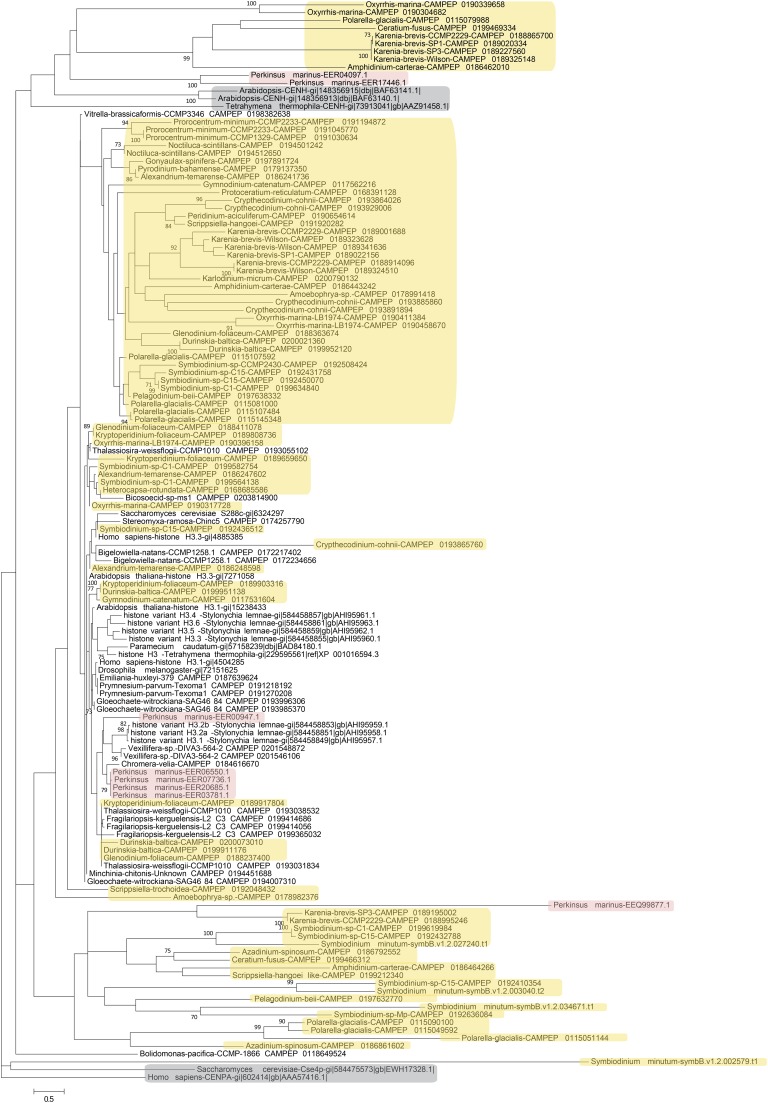
Maximum likelihood phylogenetic tree of H3 protein sequences in dinoflagellates and perkinsids. The tree was generated using RAxML (version 8.0.26) under the LG+G model and with 100 bootstrap replicates. Additional sequences from genome sequences and MMETSP assemblies of other protists were included (the eight H3 variants from the ciliate *Stylonychia lemnae*, H3 sequences from two other ciliates, *Paramecium caudatum* and *Tetrahymena thermophila*, the bicosoecid *Bicosoecid* sp., the chlorarachniophyte *Bigelowiella natans*, the two chromerids *Chromera velia* and *Vitrella brassicaformis*, the diatoms *Thalassiosira weissflogii* and *Fragilariopsis kerguelensis*, the glaucophyte *Gloeochaete witrockiana*, the haptophytes *Emiliania huxleyi* and *Prymnesium parvum*, the amoebozoans *Stereomyxa ramosa* and *Vexillifera* sp., the *Bolidomonas* heterokont, the cercozoan *Minchinia chitonis*) as well as sequences from human, yeast, flies, and *Arabidopsis*. Centromeric histone H3 variants from *Homo sapiens*, *Saccharomyces cerevisiae*, *Arabidopsis*, and *Tetrahymena thermophila* were also included and are highlighted in gray. Dinoflagellate sequences are highlighted in yellow while perkinsid proteins are marked in pink.

**Figure 7:**
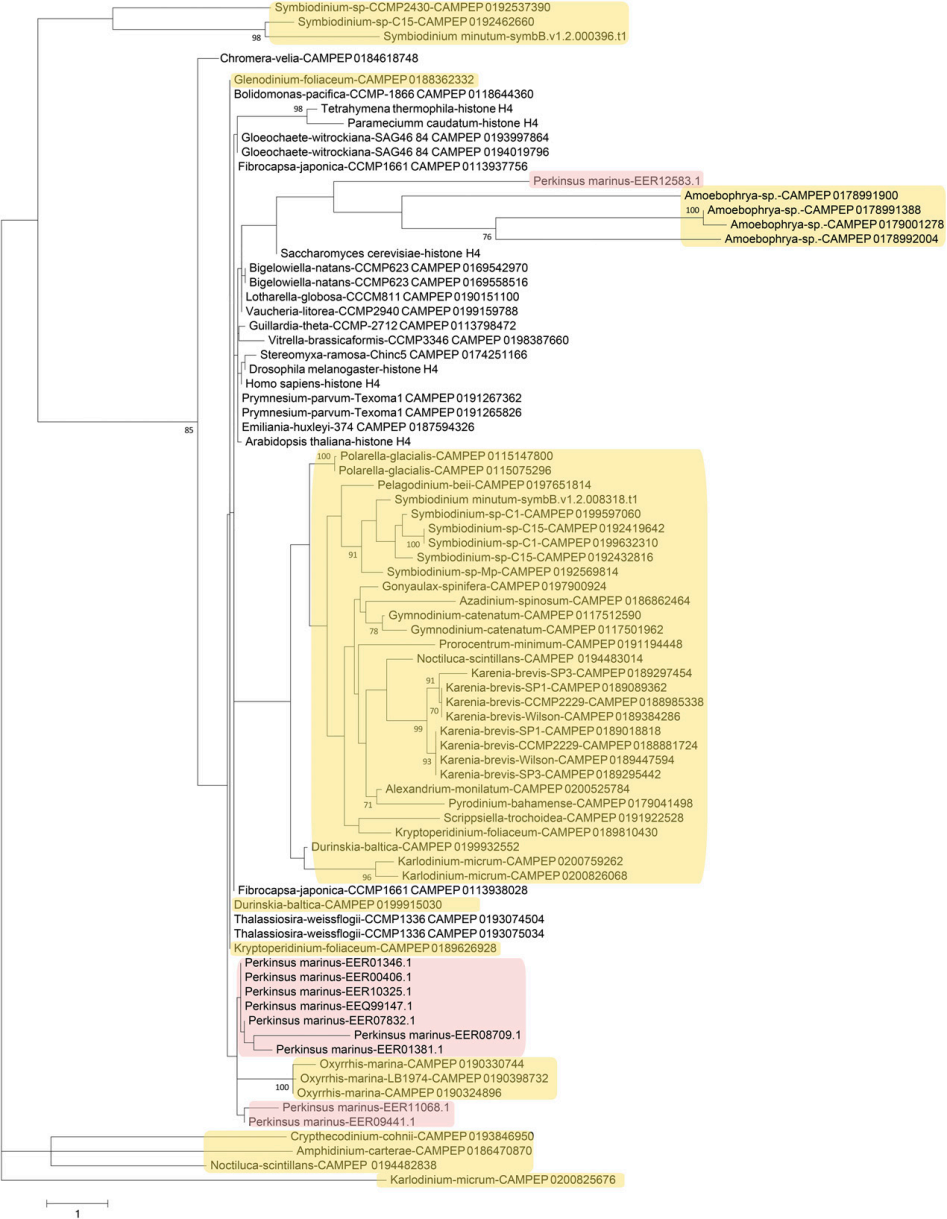
Maximum likelihood phylogenetic tree of H4 protein sequences in dinoflagellates and perkinsids. The tree was generated using RAxML (version 8.0.26) under the LG+G model and 100 bootstrap replicates. Additional sequences from genome sequences and MMETSP assemblies of other protists (the two ciliates, *Paramecium caudatum* and *Tetrahymena thermophila*, the amoebozoan *Stereomyxa ramosa*, the *Bolidomonas* heterokont, the chlorarachniophytes *Bigelowiella natans* and *Lotharella globosa*, the two chromerids *Chromera velia* and *Vitrella brassicaformis*, the cryptophyte *Guillardia theta*, the diatom *Thalassiosira weissflogii*, the glaucophyte *Gloeochaete witrockiana*, the haptophytes *Emiliania huxleyi* and *Prymnesium parvum*, the raphidophyte *Fibrocapsa japonica*, the xanthopyte *Vaucheria litorea*) were included as well as sequences from human, yeast, flies, and *Arabidopsis*. Dinoflagellate sequences are highlighted in yellow while perkinsid proteins are marked in pink.

Next, phylogenetic analysis was carried out on histones from dinoflagellates and from a number of other unicellular and multicellular species (Figure 4, Figure 5, Figure 6, and Figure 7). Most eukaryotes express multiple variants of the four core histones (Talbert *et al.* 2012); some of the additional variants are thought to be ancestral to all eukaryotes. In particular, H2A.Z is often incorporated in nucleosomes surrounding transcription start sites, and specific variants of histone H3 associate with centromeres. H2A.Z and centromeric H3 from several divergent eukaryotes were also included in the analysis in order to identify their putative dinoflagellate homologs (Figure 4 and Figure 6). However, it should be noted that such functional homologs might not be identifiable from sequence alone, as many histone variants are known to be rapidly evolving and/or to be polyphyletic or paraphyletic (Talbert *et al.* 2012), and numerous cases of loss, gain, and replacement of variants, even between closely related species, have been documented (Baldi and Becker 2013; Drinnenberg *et al.* 2014; Wang *et al.* 2011).

The patterns emerging from these comparisons are complex, with the deep branches of all trees being poorly resolved. This is particularly true for H2A and H2B, which is not surprising as these are in general the most variable core histones within eukaryotes and exhibit the highest degree of innovation in terms of novel histone variants in different lineages.

Within H2A histones, a group of putative H2A.Z variants is observed (Figure 4), but in addition to it, there are several other very deep branches consisting of proteins that might serve as novel variants with currently unknown functions. One other histone variant can be identified from sequence – H2A.X, which is best known for its role during DNA damage response (Ismail and Hendzel 2008). H2A.X is characterized by the presence of a SQ(E/D)Φ phosphorylation motif at the C-terminus of the protein (Talbert *et al.* 2012), the exact sequence of which varies but is generally constant within the broad divisions of eukaryotes. A number of dinoflagellate histones do contain similar motifs at their C-terminus, but a great deal of diversity is observed among them (Table S2): SQEF, SQEY, SQQY, and SQDF motifs are all observed. Of note, most of the H2A variants in the *P*. genome contain SQ(E/D)Φ motifs, one being SQEI, and eight SQEM; the functional significance of having so many putative H2A.X variants is currently unclear.

Excluding putative H2A.Z variants, a few dinoflagellate H2A proteins cluster closely with conventional H2A, but most of these are from dinotoms and very similar to H2A sequences from the diatom *Thalassiosira*, suggesting that they are of endosymbiont origin. The majority of dinoflagellate H2A sequences are highly divergent. Similar patterns are observed for histone H2B (Figure 5), with a dinotom-specific cluster, many highly derived sequences from both dinotoms and other dinoflagellates, and a small number of proteins clustering with conventional core histone H2B.

Deeply diverging sequences also constitute the majority of dinoflagellate histone H3 proteins (Figure 6). No group of dinoflagellate histones that robustly clusters with centromeric H3 sequences from other eukaryotes could be identified, but given the very rapid evolution of centromeric H3 proteins, this is not surprising. Thus, the presence of centromeric histones in dinoflagellates cannot be excluded. Notably, several deeply diverging H3 variants are also observed in *P. marinus*.

Histones H3.1 and H3.3 form a pair of sequence variants shared between many eukaryotes. H3.1 is deposited during the S-phase of the cell cycle while H3.3 replaces it as a result of transcriptional activity outside of it (Hake and Allis 2006; Otero *et al.* 2014). H3.3 and H3.1 are distinguished by a very small number of residues, in particular and classically, the presence at position 31 of an S (metazoans) or T (plants) in H3.3 *vs.* A in H3.1; some other variations on that theme are also observed in other eukaryotes (Talbert *et al.* 2012). In a few dinoflagellates, including *A. tamarense*, some *Symbiodinium* isolates, and *Heterocapsa rotundata*, pairs of very similar histones distinguished by the presence or absence of a phosphorylatable amino acid at position 31, are observed (Figure S8). Although there are only a few such species and their phylogenetic distribution is patchy, these observations do suggest that such pairs of H3 variants might be present in dinoflagellates, especially if one of the other distinctions between H3.3 and H3.1 also holds, namely that the former is replication-independent and with polyadenylated mRNAs while the other is replication-dependent and with mRNAs lacking polyA tails (Marzluff *et al.* 2008), and is thus systematically under-represented in polyA-selected RNA-seq libraries.

Histone H4 displays the most striking divergence among dinoflagellate histones (Figure 7). Aside from the dinotom-specific group, most dinoflagellate H4 proteins form a well-defined group of highly derived sequences. In addition, a *Symbiodinium*-specific deeply divergent variant is observed, and *Amoebophrya* expresses four even more divergent variants. Curiously, such variants are also found in the *P. marinus* genome, in addition to the more conventional H4 variants it contains. Many of these are elongated variants. As H4 is the most conserved of all histones and also the one with the fewest known variants (Talbert *et al.* 2012), these are especially intriguing observations in terms of their functional implications.

### Conservation and divergence of the histone code in dinoflagellates

As discussed above, the question of the functional importance of dinoflagellate histones is closely related to the extent of conservation and divergence of the dinoflagellate histone code. To address this question, the conservation of key post-translationally modified histone residues relative to other eukaryotes was assessed. Before the results from these comparisons are described, a brief overview of the significance of these residues is presented.

#### The histone code:

Hundreds of histone modifications have been identified (Huang *et al.* 2014), including monoubiquitination, acetylation, mono-, di-, and trimethylation of lysines, mono- and symmetric and asymmetric dimethylation of arginines, phosphorylation of serines, threonines, and tyrosines, isomerization of prolines, and others (Figure S9). While the great majority have not been characterized in any detail, the role of a significant number is fairly well understood, which has revealed both the functional importance of the histone code and the strong constraints that it imposes on the corresponding residues across eukaryotes. The presence or absence of these residues in dinoflagellate histones can be highly informative about the conservation of the relevant aspects of the histone code and chromatin biology. This approach has some limitations, as conservation of amino acid residues need not be solely due to their PTMs, and does not on its own mean that the relevant modifications are in fact deposited *in vivo* and have a conserved function. However, the absence of an important modified residue does mean that the corresponding histone marks are not conserved, and that their functionality has been lost (or, possibly, adopted by other residues elsewhere on nucleosomes).

The key histone marks in eukaryotes and their functions, as revealed by studies in yeast, mammals, and other model systems will be briefly described before the state of the histone code in dinoflagellates is discussed. The list is by no means exhaustive and the discussion is by necessity simplified as the complexity of the histone code is immense. The focus will be on a subset of marks associated with the following processes: transcriptional activation, initiation, and elongation, the formation of heterochromatin and other repressive chromatin environments, and the regulation of chromatin dynamics during mitosis.

##### Repression and heterochromatin:

Heterochromatinization is a major mechanism for the permanent repression of transposable elements and some host genes in eukaryotes; heterochromatin is also often found in telomeric and centromeric regions. The main mechanisms for establishing and maintaining heterochromatin are deeply conserved (including in other alveolates; Liu *et al.* 2007; Schwope and Chalker 2014) and likely ancestral to all eukaryotes, with histone marks playing a key role. The classic heterochromatin histone mark is the trimethylation of lysine 9 of histone H3 (H3K9me3). H3K9me3 recruits the heterochromatin protein HP1 (Lachner *et al.* 2001; Bannister *et al.* 2001), which leads to compaction of chromatin. It is also bound by H3K9me3 methyltransferases (Tachibana *et al.* 2001), which can further methylate neighboring H3K9 residues, and has a positive feedback loop relationship with cytosine DNA methylation (Stancheva 2005).

Another mechanism for gene repression is mediated by the H3K27me3 mark and involves the action of Polycomb proteins, which both deposit and are recruited by it. The process has been extensively characterized in both plants and metazoans (Zheng and Chen 2011; Simon and Kingston 2013), and also involves the monoubiquitination of histone H2A (H2AK119ub1; Shilatifard 2006).

Additional histone marks associated with repressive chromatin include H4K20me3 (Balakrishnan and Milavetz 2010) and H3K64me3 (Daujat *et al.* 2009). It should be noted that heterochromatin is not a homogeneous entity, and that different marks or combinations of marks may characterize distinct types of repressive chromatin.

##### Transcription activation and initiation:

The first histone modification to be assigned potential functional significance was histone acetylation, which usually exercises a general stimulating effect on transcription by reducing the positive charge of histones and loosening histone–DNA interactions (Allfrey *et al.* 1964). Many lysines on histones are acetylated (Figure S9; the ones discussed in some detail here include: H3K9ac, H3K14ac H3K18ac, H3K23ac, H3K27ac, H3K56ac, H3K64ac, H4K5ac, H4K8ac, H4K12ac, H4K16ac, H4K59ac, H4K79ac, H4K91ac, H2AK4ac, H2AK8ac, H2AK12ac, H2BK5ac, H2BK12ac, H2BK16ac, and H2BK20ac), but this general activating effect is not their sole function. For example, H3K27ac has been identified as a marker of active enhancer elements in metazoans (Rada-Iglesias *et al.* 2010; Creyghton *et al.* 2010), H3K56ac plays a role in the activation of transcription but is also important for histone exchange and nucleosome assembly (Rufiange *et al.* 2007; Xu *et al.* 2005; Xie *et al.* 2009), etc.

In all eukaryotes studied so far, active promoters are specifically marked by H3K4me3. The exact biochemical mechanisms in which H3K4me3 is involved have not yet been completely elucidated (Shilatifard 2012), but its deep conservation suggests that it plays a key role in the process of initiation.

In addition, multiple histone marks have been correlated with transcription activation, but the mechanistic details of their role are not entirely clear. These include the phosphorylation of threonine 6 on histone H3 (H3T6ph), which prevents the demethylation of H3K4me3 (Metzger *et al.* 2010), H3S28ph, which marks active promoters and transcribed gene bodies (Drobic *et al.* 2010; Sawicka *et al.* 2014) and cross-talks negatively with H3K27me3 (Fischle *et al.* 2003), and several arginine methylations. H3R2 is known to be both symmetrically and asymmetrically methylated, with asymmetric methylation (H3R2me2a) being mutually exclusive with H3K4me3 (Guccione *et al.* 2007; Kirmizis *et al.* 2007) while H3R2me2s correlates positively with it (Yuan *et al.* 2012). Similarly, H4R3me2s is associated with repression (Tsutsui *et al.* 2013; Dhar *et al.* 2012), and H4R3me2a with activation (Sun *et al.* 2011).

##### Transcription elongation:

The eukaryote transcription cycle involves a complexly choreographed sequence of nucleosome remodeling events and PTMs on histones and the polymerase itself. Because nucleosomes are inhibitory to it, the nucleosome barrier has to be overcome for transcription to proceed. This is achieved through a combination of histone acetylation and the action of the FACT complex (Reinberg and Sims 2006; Pavri *et al.* 2006; Zhu *et al.* 2005), which partially disassembles nucleosomes, allowing for the polymerase to proceed, and then reassembles them. The action of FACT is associated with the transient monoubiquitination of histone H2B (H2BK120ub1 in mammals).

The role of H3K36me3 in transcription elongation is particularly well understood mechanistically (Wagner and Carpenter 2012). The acetylation marks deposited during elongation need to be erased if cryptic transcription from within the now more open chromatin is to be prevented (Kaplan *et al.* 2003). H3K36me3 is deposited by methyltransferases (KMTs) associated with the elongating polymerase, and then serves as a recruitment mark for histone deacetylases (HDACs), which remove the acetylation marks and close the cycle. However, this is not the only function of H3K36me3, as it has also been shown to play a significant role in the process of splicing in metazoans (Kolasinska-Zwierz *et al.* 2009; Schwartz *et al.* 2009).

Another site of modification associated with elongation is H3K79 (H3K79me2/3; Nguyen and Zhang 2011). H3K79me is also notable for being catalyzed by the Dot1 KMT (Feng *et al.* 2002) rather than a SET-domain methyltransferase as all other lysine methylations on histones.

An additional mark involved in elongation is H2AY57ph, which appears to be required for the H2BK120 ubiquitination/deubiquitination cycle (Basnet *et al.* 2014).

Finally, the isomerization of prolines 30 and 38 in H3 (H3P30iso, H3P38iso) can regulate the activity of H3K36 methyltransferases (Nelson *et al.* 2006).

##### Mitosis:

Chromatin undergoes dramatic transformations during mitosis, as it is duplicated in the S-phase and then compacted in the M phase. Histone marks play a key role in these processes (Desvoyes *et al.* 2014; Doenecke 2014), in particular several phosphorylatable residues on histones H3, H4, and H2A (Sawicka and Seiser 2012).

H3S10ph, although it can also occur in other contexts, is the best characterized such mark, involved in chromosomal condensation during mitosis (Hendzel *et al.* 1997). A key event during this process is the establishment of an interaction between the N-terminal tail of histone H4 and an acidic patch on H2A-H2B dimers on neighboring nucleosomes, which, however, is prevented by the presence of H4K16ac; H3S10ph recruits HDACs, which remove acetylation marks from H4 tails, allowing compaction to happen (Wilkins *et al.* 2014). H3S10ph also has the effect of excluding HP1, which is ejected from chromatin during the M phase, as HP1 cannot bind to H3 tails containing both H3K9me3 and H3S10ph (Fischle *et al.* 2005).

Additional marks implicated in the regulation of mitotic chromatin dynamics include H3T3ph (Polioudaki *et al.* 2004; Wang *et al.* 2010), H3T11ph (Preuss *et al.* 2003), which is also involved in transcriptional activation (Metzger *et al.* 2008), H3S28ph, H4S1ph and H2AS1ph (Barber *et al.* 2004), H2AS122ph (Yamagishi *et al.* 2010), H2AT120ph (van der Horst *et al.* 2015), and others. Most of these marks are conserved between plants and humans although the precise patterns of their chromosomal distribution may differ somewhat (Houben *et al.* 2007; Manzanero *et al.* 2000).

Histone marks also play a role in nucleosome assembly during and independently of replication. H3K56ac, which occurs in the core histone domain rather than the tail, facilitates histone exchange (Rufiange *et al.* 2007); other marks suggested to play such a role include H4K79ac and H4K91ac (Yang *et al.* 2011).

#### Conservation and divergence of dinoflagellate histone tails:

**Figure 8:**
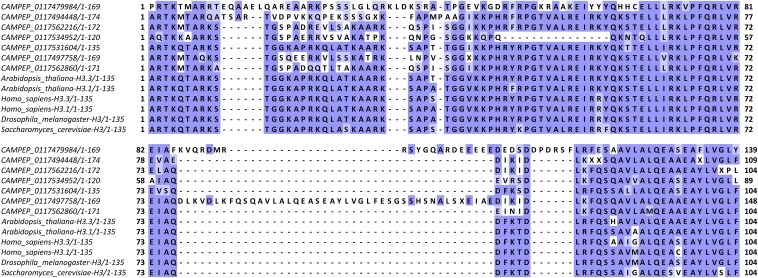
Multiple sequence alignments of the N-terminal regions of histone H3 sequences from *Gymnodinium catenatum* and of core histone H3 proteins from several other eukaryotes. Sequences were aligned using MUSCLE (Edgar 2004; version 3.8.31) and visualized using JalView (Waterhouse *et al.* 2009; version 2.8.2).

In the light of the histone code, many dinoflagellate histones present a curious mixture of conserved and divergent characters (Figure 8 shows H3 tails in *Gymnodinium catenatum*). The most well-known H3 variants are centromeric histones, and those usually have highly divergent histone tails (Figure S10). There are many examples of such tails in dinoflagellates, but there are also histones with tails fairly similar to the conventional state, and all sorts of variations between these two extremes. Thus *G. catenatum* expresses a variant that is a close match to the typical H3 sequence (CAMPEP_0117531604), and two very divergent ones (CAMPEP_0117479984 and CAMPEP_0117494448), one or both of which exhibit substitutions at many key residues of the histone code, including H3K9, H3K27, H3K36, and H3K79, as well as several insertions within the N-terminal tails and the core histone domain. The other variants in *Gymnodinium* show an intermediate level of divergence, with the region between residues 12 and 31 being particularly variable.

Similar observations can be made in many other species, although because of the certainly incomplete sampling of variants in transcriptome assemblies, such a comprehensive set of variations is not always seen.

#### The histone code in dinoflagellates:

To assess the conservation of the histone code, multiple sequence alignments of all variants of each core histone in each species and a reference (taken to be the human core histones, with histone H3.1 used in the case of H3) were carried out. All histone sequences with complete N-terminal tails were used for this analysis (*i.e.*, a protein was allowed to be incomplete at its C-terminus and not just the “complete” sequences shown in Figure 1 were included). This was done in order to capture all the histone tail diversity for which there is evidence in the data.

Conservation was then scored against the reference sequence as follows: for a given position *i* in the reference protein and a radius *r*, a precise match of the sequence with coordinates [i−r,i+r] was required to score that residue as conserved, *i.e.*, when r=0, only a conservation of the residue itself is required, but when r=1 and r=2, the 3-amino acid and 5-amino acid contexts were considered. This approach was adopted as histone marks are often deposited and read according to their sequence context. Thus, conservation of the context makes it more plausible that the modification is also conserved. Of course, there are limitations to such interpretations: residues and their context need not be conserved because of conservation of a particular histone mark, and the absence of strict conservation of context does not necessarily mean that the mark is not deposited (its reader and writer proteins might have evolved and adapted accordingly). Nevertheless, it remains true that the complete absence of a residue is almost certain evidence that the corresponding histone marks are absent too, and that strong conservation of its context boosts confidence in the preservation of at least the capacity to deposit them.

**Figure 9:**
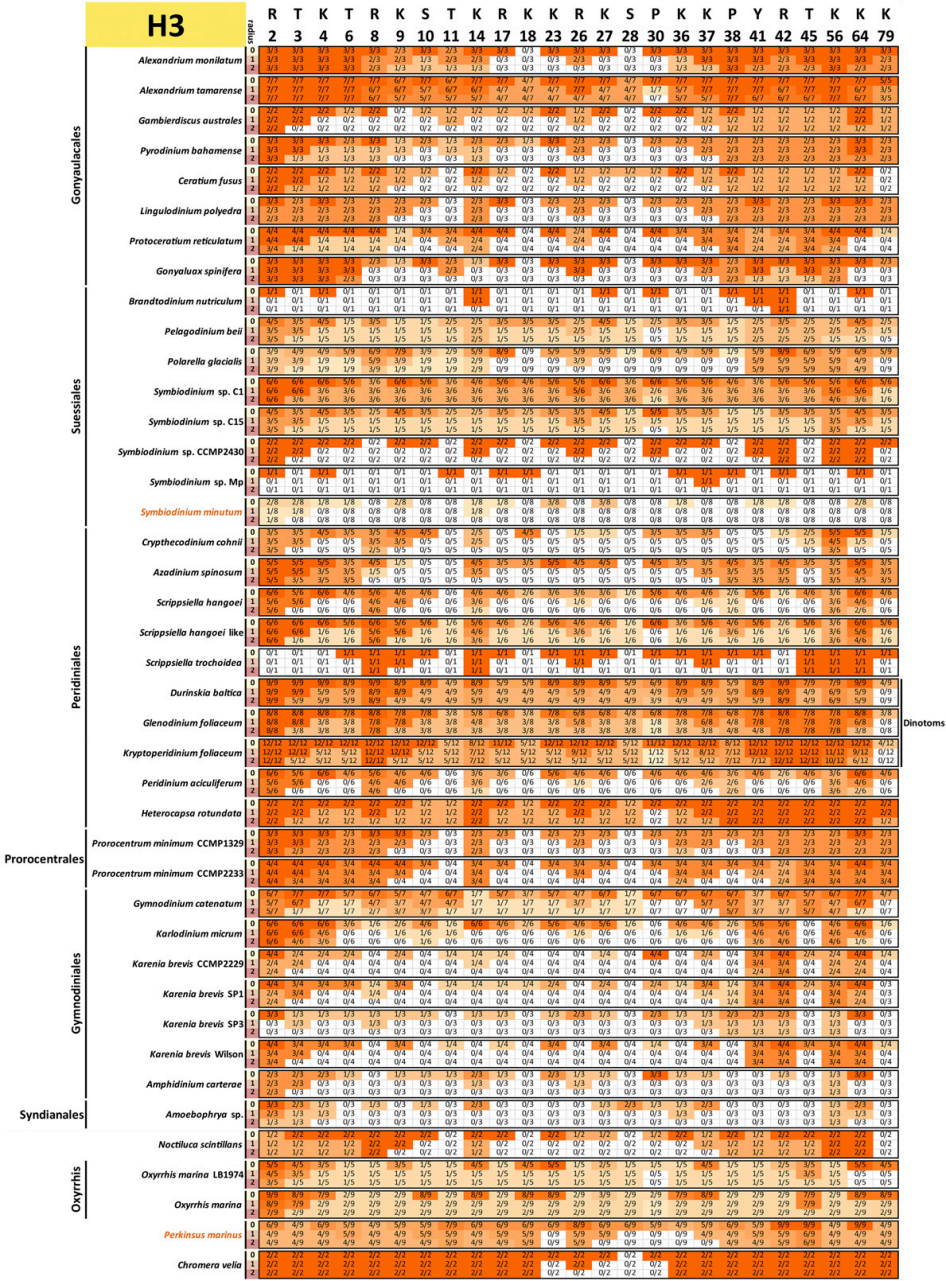
Conservation of key post-transcriptionally modified residues in dinoflagellate histone H3 proteins. The radius *r* refers to the size of the context considered when scoring conservation. When r=0, only the residue itself is considered; when r=1, a perfect match to the three-amino acid peptide also including the flanking residues on each side is required; when r=2, the five-amino acid peptide also including the two flanking residues on each side is considered. The fractions C/N indicate the number of histone H3 proteins *C* with conserved residues according to the criteria specified by the radius relative to the total number of histone H3 proteins *N* for each species. All histone H3 sequences with complete N-terminal tails identified in the transcriptomic data are included (the C-terminal portions of the protein were allowed to be incomplete for the purposes of this analysis, but the *N* factor in the C/N fractions was adjusted accordingly if necessary in cases when gaps in the alignments were due to an incomplete sequence).

**Figure 10:**
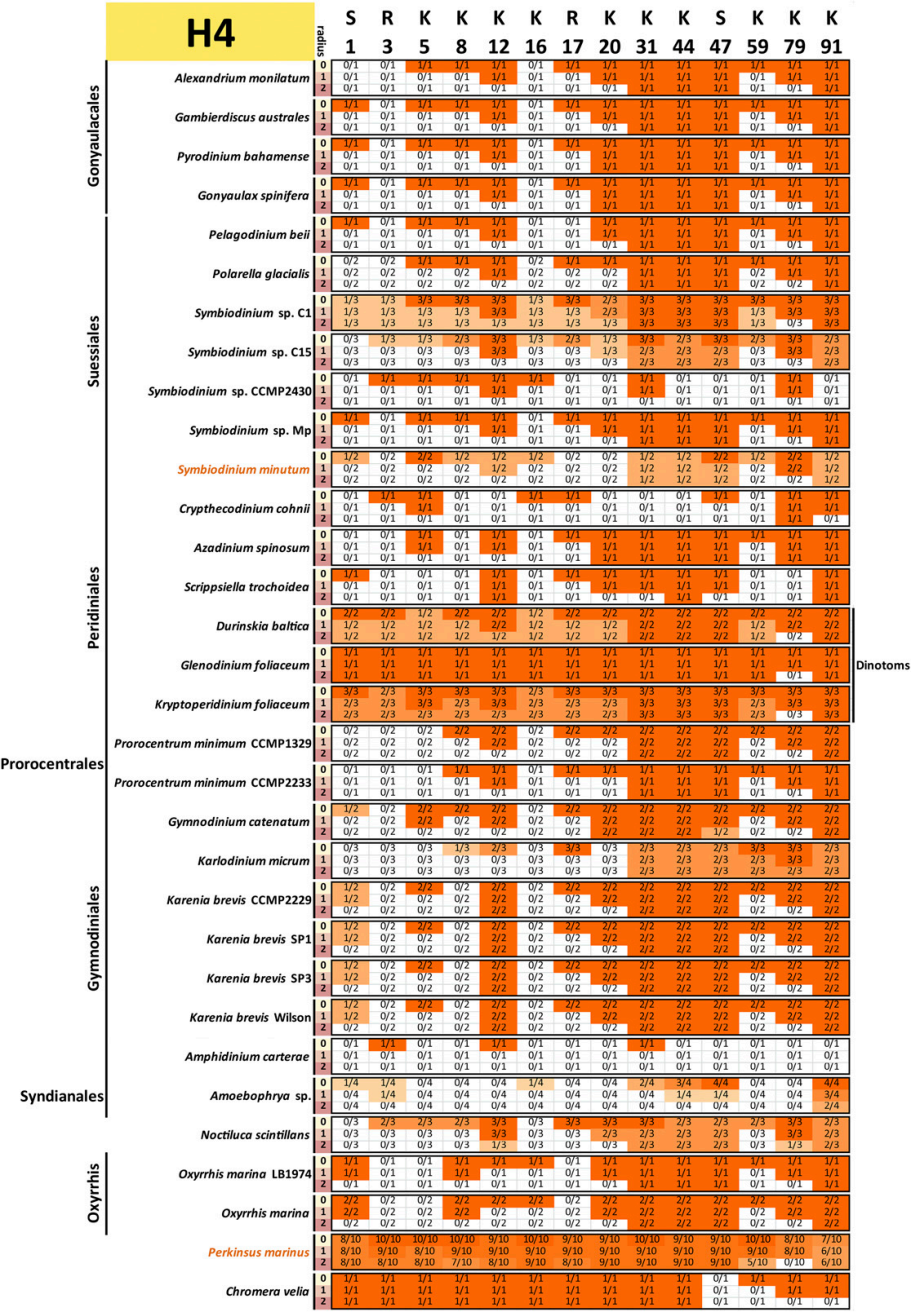
Conservation of key post-transcriptionally modified residues in dinoflagellate histone H4 proteins. The radius *r* refers to the size of the context considered when scoring conservation. When r=0, only the residue itself is considered; when r=1, a perfect match to the three-amino acid peptide also including the flanking residues on each side is required; when r=2, the five-amino acid peptide also including the two flanking residues on each side is considered. The fractions C/N indicate the number of histone H4 proteins *C* with conserved residues according to the criteria specified by the radius relative to the total number of histone H4 proteins *N* for each species. All histone H4 sequences with complete N-terminal tails identified in the transcriptomic data are included (the C-terminal portions of the protein were allowed to be incomplete for the purposes of this analysis, but the *N* factor in the C/N fractions was adjusted accordingly if necessary in cases when gaps in the alignments were due to an incomplete sequence).

**Figure 11:**
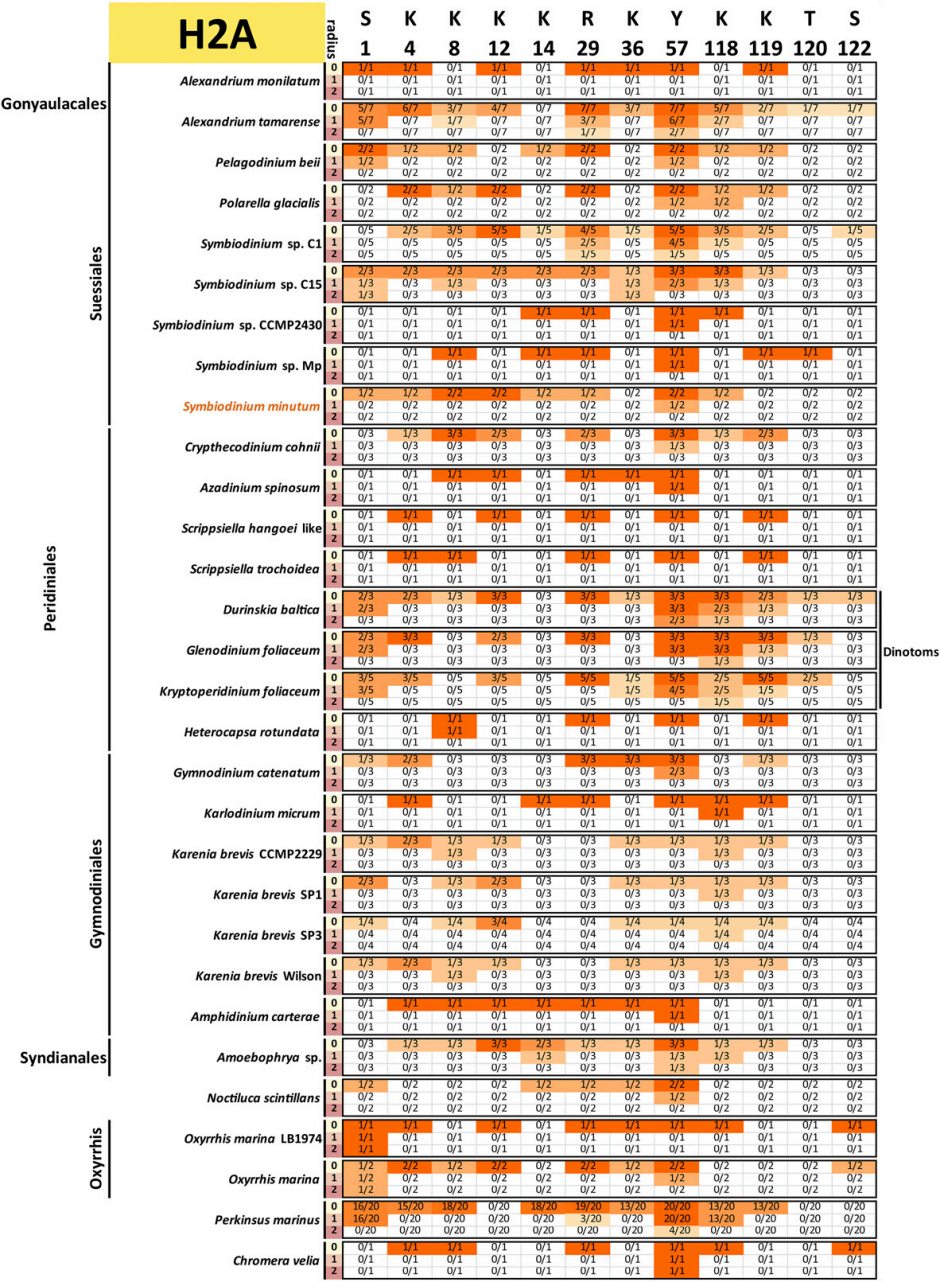
Conservation of key post-transcriptionally modified residues in dinoflagellate histone H2A proteins. The radius *r* refers to the size of the context considered when scoring conservation. When r=0, only the residue itself is considered; when r=1, a perfect match to the three-amino acid peptide also including the flanking residues on each side is required; when r=2, the five-amino acid peptide also including the two flanking residues on each side is considered. The fractions C/N indicate the number of histone H2A proteins *C* with conserved residues according to the criteria specified by the radius relative to the total number of histone H2A proteins *N* for each species. All histone H2A sequences with complete N-terminal tails identified in the transcriptomic data are included (the C-terminal portions of the protein were allowed to be incomplete for the purposes of this analysis, but the *N* factor in the C/N fractions was adjusted accordingly if necessary in cases when gaps in the alignments were due to an incomplete sequence).

**Figure 12:**
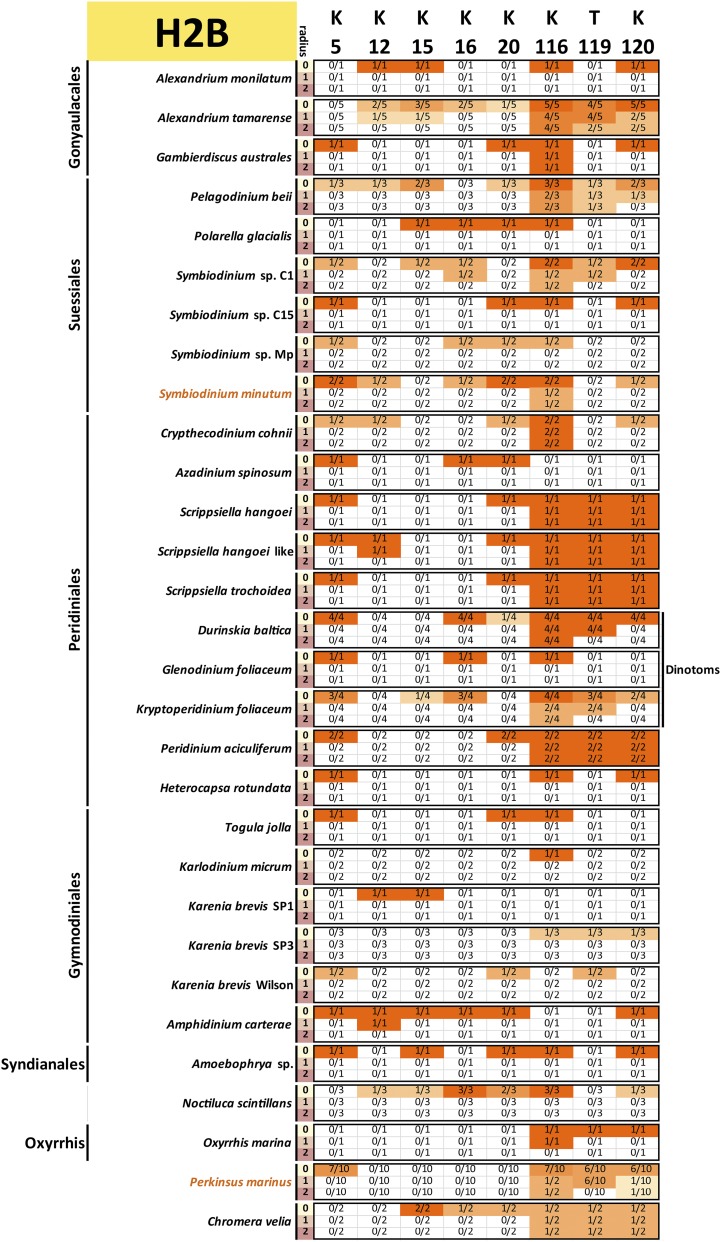
Conservation of key post-transcriptionally modified residues in dinoflagellate histone H2B proteins. The radius *r* refers to the size of the context considered when scoring conservation. When r=0, only the residue itself is considered; when r=1, a perfect match to the three-amino acid peptide also including the flanking residues on each side is required; when r=2, the five-amino acid peptide also including the two flanking residues on each side is considered. The fractions C/N indicate the number of histone H2B proteins *C* with conserved residues according to the criteria specified by the radius relative to the total number of histone H2B proteins *N* for each species. All histone H2B sequences with complete N-terminal tails identified in the transcriptomic data are included (the C-terminal portions of the protein were allowed to be incomplete for the purposes of this analysis, but the *N* factor in the C/N fractions was adjusted accordingly if necessary in cases when gaps in the alignments were due to an incomplete sequence).

The results from these comparisons for histones H3, H4, H2A, and H2B are shown in Figure 9, Figure 10, Figure 11, and Figure 12, respectively.

Before these results are discussed, it should be noted that only very divergent H3 histones are found in the assembled *S. minutum* contigs (even the residues that are conserved in one of the annotated variants according to the criteria used above are found in the context of an otherwise very divergent N-terminal tail). However, the transcriptomes of other *Symbiodinium* isolates contain more conventional histone H3 sequences, a discrepancy that, if contamination is to be excluded, is best explained as being due to the incompleteness of the *S. minutum* assembly, which is known to capture only a fraction of the total genomic sequence (Shoguchi *et al.* 2013).

##### Repression and heterochromatin:

H3K9 displays an intriguing pattern of presence and absence in dinoflagellates. It is present in at least one variant in the majority of species (with the exception of a few transcriptomes, in which only a single variant was assembled, and *Amoebophrya* and *Gambierdiscus australes*, in which all three and two H3 variants, respectively, do not have it), although its sequence context is often not well conserved. However, most species also have multiple variants without H3K9. Many of these belong to the group of variants with extended protein sequence and very divergent tails, which are more likely to have specialized functions such as those of centromeric H3 histones in other eukaryotes. Yet quite remarkably, H3K9 is not absent solely from H3 tails that are highly divergent; for example, *A. monilatum* expresses a 167-amino acid histone H3 (the increased length is due to a C-terminal extension), the N-terminal portion of which is overall a close match to human histone H3 with the notable exception of H3K9, which is substituted by a methionine (Figure S11). Functional homologs of HP1 have been identified in the two other major alveolate lineages, ciliates (Schwope and Chalker 2014) and apicomplexans (Flueck *et al.* 2009), but no clear homologs (defined as proteins with a pair of chromodomains and a chromo shadow domain) were detected in dinoflagellate transcriptomes. None were found in *C. velia* or *P. marinus* either, but at least in the case of *C. velia* this is most likely a false negative case. As H3K9 is also subject to numerous other post-transcriptional modifications, the possibility that its conservation is for other reasons and that the overall H3K9me3/HP1 heterochromatin formation mechanism is not conserved cannot be dismissed.

Almost all dinoflagellates possess at least one histone H3 variant with H3K27 (more than half of the variants possessing H3K27 is the typical condition), although as in the case of H3K9, its sequence context is not well conserved (because the neighboring H3S28 is usually missing). The number of H2A variants assembled is often low, thus it is not certain that all are present in the assemblies, but in most species a variant with H2AK119 (often ubiquitinated in concert with H3K27me3 deposition) is present.

H4K20 is observed in most dinoflagellate H4 histones with the exception of the four variants in *Amoebophrya* and the two variants in the *S. minutum* genome assembly.

H3K64 is a remarkably well conserved residue (although not always in its sequence context), being present in at least one (usually most) H3 variant in all dinoflagellates. As H3K64 resides in the core histone domain, this might be due to constraints other than its role in heterochromatin formation, but its strong conservation suggests that if nucleosomal heterochromatin does form in dinoflagellates, it might be playing a role in the process.

Overall, the analysis of heterochromatin-associated histone mark-bearing residues suggests that while the capacity to deposit these marks has been retained in most dinoflagellates, the constraints on their sequence context seem to have been relaxed. This is possibly due to a significant reduction of the amount and functional importance of nucleosomal heterochromatin, or even its complete loss.

##### Transcription activation and initiation:

With the exception of H4K12, the majority of acetylated lysines in the N-terminal histone tails display poor conservation across dinoflagellates. This is not entirely surprising as these lysines are known to be redundant with each other (Dion *et al.* 2005; Martin *et al.* 2004), and could be lost if constraints on histone sequence are relaxed.

The status of conservation of H3K4 is of particular interest given its strong association with active transcription start sites (TSSs) in other eukaryotes. It is found in all dinoflagellates (with the exception of the single divergent H3 variant assembled in *Brandtodinium nutriculum*), usually in most variants, and often in a well-conserved sequence context. Variants without it are also observed, and as with H3K9, these are not always of the elongated highly divergent varieties, but overall these observations suggest retention of the functional importance of H3K4me3 in dinoflagellates.

Other, less well characterized residues such as H3R2 and H3T6 are also fairly well conserved, but it could be that this is due to constraints on the H3K4 sequence context. Another methylated arginine, H4R3, displays a remarkable absence of conservation, being found in few dinoflagellates outside of dinotoms (but well conserved in *Perkinsus* and *Chromera*).

The conservation status of these marks suggests preservation of the pivotal role of H3K4me3 and a derived state for some other components of the histone code associated with the activation and repression of transcription.

##### Transcription elongation:

H3K36 is observed in the majority of dinoflagellates (with the possible exception of *Karenia brevis* among the species with a large number of assembled H3 variants), suggesting the possible conservation of its role in transcription elongation. H3K79 is less well conserved although still found in most species. The presence/absence pattern of H2BK120 is patchy, but this might well be due to incomplete assemblies as the number of observed H2B variants is often low.

The two prolines subject to isomerization, H3P30 and H3P38, are also found in most dinoflagellates but not in all tails and not always together.

Somewhat surprisingly, the residue implicated in transcriptional elongation that displays the strongest conservation is H2AY57.

Overall, these observations suggest that the capacity to deposit the key histone marks involved in transcriptional elongation is present, with some divergence, and the processes they are involved in might be conserved in dinoflagellates.

##### Mitosis:

The phosphorylatable residues involved in mitosis (H3S10ph, H3T3ph, H3T11ph, H3S28ph, H4S1ph, H2AS1ph, H2AS122ph, and H2AT120ph) display quite poor conservation in dinoflagellates. H3T3ph is an exception but this could be because of constraints on the neighboring H3K4. H3S10 is also present in at least one variant in many species, but is also completely absent in numerous others. Also remarkable is the absence of conservation of H4K16, the residue involved in the control of nucleosome compaction. Given these observations, it is quite likely that the H3K9me3S10ph switch does not operate in dinoflagellates and that histone phosphorylation plays a reduced (if any) role in mitosis.

Of note, all these residues are best conserved in *O. marina*, the earliest branching dinoflagellate included in this study. It is therefore possible that the corresponding processes are most preserved in *Oxyrrhis* while other dinoflagellates are in an even more derived state.

H3K56, H4K79, and H4K91, the acetylation of which has been implicated in the process of nucleosome assembly, are very well conserved across dinoflagellates, including at the level of their sequence context.

### Histone mark writers, erasers, and readers, and chromatin remodelers

Histone marks are deposited and erased by specific enzymes and read by proteins containing “reader” domains, recognizing particular modifications. Histone acetylation is carried out by several families of lysine acetyltransferases: KATs, or, in the context of histones, HATs (Marmorstein and Zhou 2014; Wang *et al.* 2008; Thomas and Voss 2007; Doyon and Côté 2004). Acetylation marks are removed by HDACs, of which there are also several classes (Mariadason 2008; Vaquero 2009), one of which includes members of the sirtuin protein family. Lysine methylation is carried out by SET-domain-containing proteins (Dillon *et al.* 2005), with the exception of H3K79 methylation and Dot1. Lysine methylation is primarily erased through the action of demethylases of the Jumonji (Jmj) family (Takeuchi *et al.* 2006). Arginine methyltransferases include PRMT5 and CARM1-type proteins (Karkhanis *et al.* 2011; Wysocka *et al.* 2006).

A variety of reader domains are known (Musselman *et al.* 2012). These include the bromodomain and the chromodomain, which bind to acetylated and methylated lysines, respectively (Sanchez *et al.* 2014; Blus *et al.* 2011), the PHD finger, which binds to a variety of unmodified and modified histone tail substrates, WD40 domains, which bind to trimethylated lysines among other targets, Tudor domains, which bind to both methylated lysines and arginines, PWWP domains, which bind to methylated lysines, and others.

ATP-dependent chromatin remodeling complexes, which move nucleosomes along DNA, are another important component of the eukaryote chromatin toolkit. Four major families of remodelers – SWI/SNF, ISWI, CHD, and INO80 – are known, distinguished by characteristic combinations of protein domains (Clapier and Cairns 2009).

**Figure 13:**
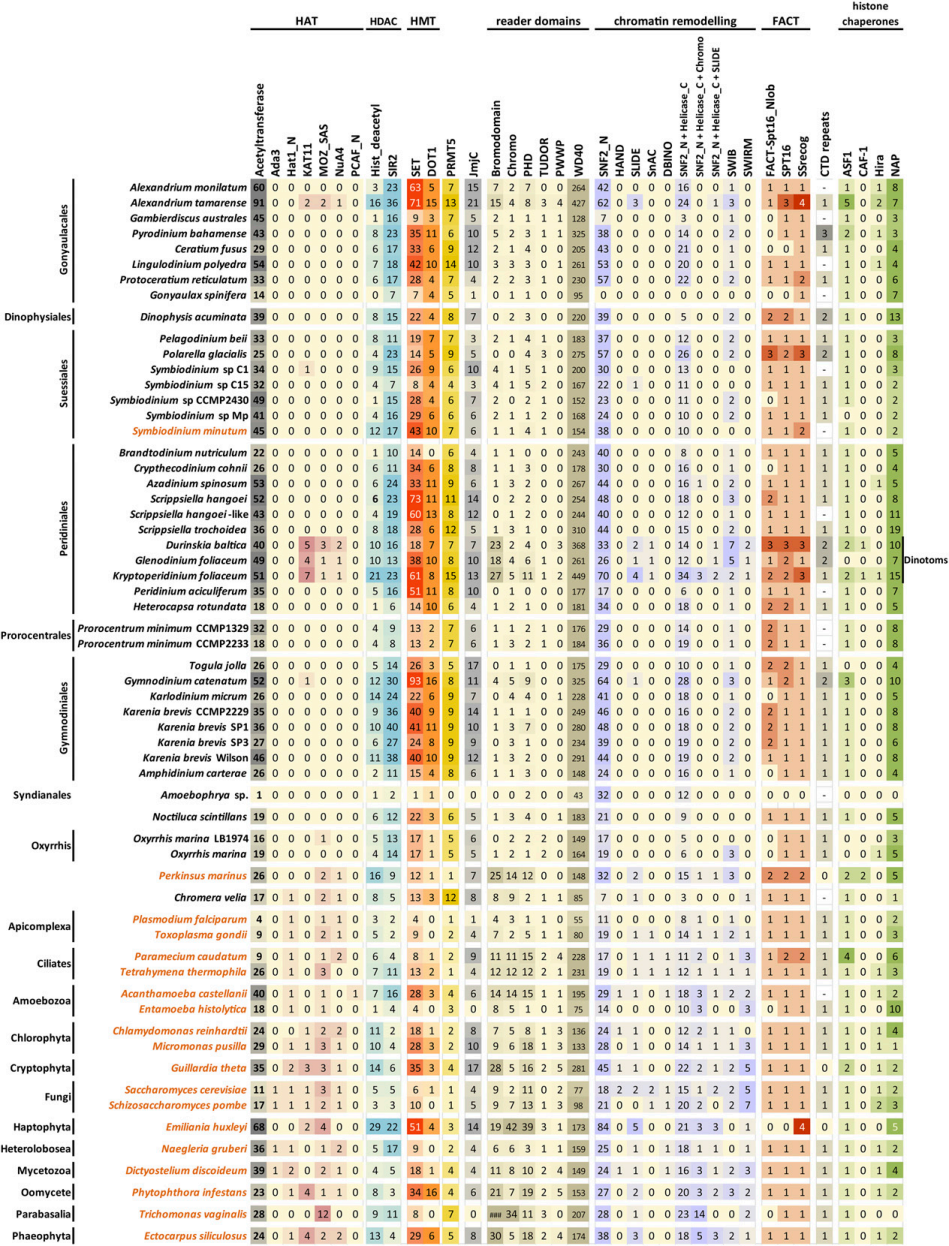
Presence and absence of histone mark writer, eraser and reader proteins, chromatin remodeling complexes, histone chaperones, the FACT complex, and RNA polymerase II CTD repeats in dinoflagellates and representative unicellular eukaryotes. There are multiple different HAT families and homologs from these were searched for separately. Except for the yeast Hat1 and the human PCAF protein, the domains found in GNAT-type acetyltransferases are also found in many acetyltransferase enzymes whose activity has little to do with chromatin, but the catalytic core of GNAT-type HAT complexes is formed by several proteins (Ada2, Ada3, Sgf29, Gcn5 in the case of the SAGA complex), and the adapter proteins (Muratoglu *et al.* 2003) were searched for in addition to acetyltransferase domains. The KAT11 domain is found in numerous HATs such as the metazoan p300 and CBP and the yeast Rtt109 (Wang *et al.* 2008). MYST-family HATs (Thomas and Voss 2007) were identified using searches for the MOZ_SAS domain, and; in addition, a search for the NuA4 domain, part of the EAF6 component of the NuA4 complex of which MYST HATs are often part of (Doyon and Côté 2004), was carried out. There are four classes of HDACs (Mariadason 2008), one of which, the sirtuins, is characterized by the presence of a SIR2 domain (although sirtuin specificity is not restricted to histones). Two classes of lysine methyltransferases are known too – the majority are of the SET-domain type, Dot1 is the exception. Known arginine methylation enzymes include PRMT5- and CARM1-type proteins (Karkhanis *et al.* 2011; Wysocka *et al.* 2006); CARM1 is not shown as no CARM1 proteins are found in any of the unicellular species shown here. Aside from the monoamine oxidase LSD1 (Rudolph *et al.* 2013), lysine demethylases contain a Jumonji domain (JmjC) (Johansson *et al.* 2014). Bromodomains (Sanchez *et al.* 2014) and chromodomains (Blus *et al.* 2011) are readers of lysine acetylation and methylation, respectively. WD40 domains bind to trimethylated lysines among other substrates, Tudor domains can bind to both methylated lysines and arginines, PWWP domains bind to methylated lysines, and PHD fingers can bind to a variety of unmodified and modified histone tail substrates (Musselman *et al.* 2012). The chromatin remodeling complexes of the SWI/SNF, ISWI, CHD, and INO80 families are characterized by the presence in their ATPase subunits of a combination of a SWI2_N and a Helicase_C domain (although the domains themselves are individually not necessarily restricted to proteins with such functions), plus additional domains in each of the families. These domains include HAND, SLIDE, SnAC, and DBINO as shown in the figure. In addition, the number of detected proteins containing both a SWI2_N and a Helicase_C domain is shown, as well as those containing all three of Chromo, SWI2_N, and Helicase_C domains (*i.e.*, putative CHD-family ATPases) and proteins with all three of the SLIDE, SWI2_N, and Helicase_C domains (putative ATPases in the ISWI family). SWIB and SWIRM are domains found in some of the noncatalytic components of SWI/SNF chromatin remodeling complexes. The FACT complex consists of two proteins, Spt16 and SSRP1; the latter contains the SSrecog domain. In addition to nucleosome chaperoning during transcription elongation, multiple histone chaperones are involved in replication-coupled and replication-independent nucleosome assembly and histone exchange (Burgess and Zhang 2013); among them, NAP1 loads H2A-H2B dimers (Mosammaparast *et al.* 2002), while CAF-1 and HIRA transfer H3-H4 dimers (Verreault *et al.* 1996; Ray-Gallet *et al.* 2002) during and independent of replication, respectively, which are in turn loaded onto them by ASF1 (English *et al.* 2006). The figure shows the number of proteins detected containing each of the domains (a threshold of $e\leq 10^{-8}$ was applied using HMMER3.0), with the exception of the CTD repeats of the Rpb1 subunit of RNA polymerase II, which were manually annotated in putative Rpb1 homologs (note that in that case “0” means that CTD repeats are not apparent in the Rpb1 homolog, while “–” refers to absence of detection of an Rpb1 homolog). The results for the organisms labeled in orange are based on available genome assemblies in contrast to those lettered in black, which are derived from transcriptome assemblies.

To evaluate the composition of the set of writer, eraser, and reader proteins and chromatin remodelers in dinoflagellates, putative homologs were identified by scanning datasets for the presence of their characteristic domains, or combinations of domains (Figure 13). In addition to *Perkinsus* and *Chromera*, the same analysis was also carried out on the sequenced and annotated genomes of a diverse set of unicellular eukaryotes, in order to compare the number of proteins in each group to what is observed in other protozoans.

Remarkably, most dinoflagellates contain a larger number of sirtuins and HDACs than most other eukaryotes. While these deacetylases need not be acting on histones (especially sirtuins), these observations support the occurrence of histone acetylation and deacetylation in dinoflagellates.

The corresponding HAT enzymes are more difficult to identify. There are multiple families of HATs, and not all of them act specifically on histones. In particular, the GCN5-related N-acetyltransferases type (GNAT-type) HATs also acetylate a wide variety of other substrates and are not even restricted to eukaryotes (Xie *et al.* 2014); these are abundant in dinoflagellates but it is not certain that they acetylate histones. For these reasons, the adapter components of the chromatin modifying complexes, in which they are usually found, were searched for. However, none were detected in any of the dinoflagellate species examined. Both KAT11 and MYST HATs were found in dinotoms, in *P. marinus*, and in *A. tamarense* among the dinoflagellates; a KAT11 HAT is found in one of the *Symbiodinium* isolates and in *Gymnodinium*, and a MYST HAT is found in *Oxyrrhis*. No other obvious HATs were found, thus the most likely explanation is that dinoflagellate HATs primarily belong to the GNAT family, and might participate in chromatin modifying complexes of derived composition.

The number of SET-domain proteins in dinoflagellates is strikingly large and exceeds anything observed in other eukaryotes. Not only that, but numerous Dot1 proteins are also observed, as is an expansion of the PRMT5 family. Accordingly, the diversity of Jmj proteins is also large. These are certainly underestimates given that they are based on transcriptomes and not complete genomes, although the extent of actual functional diversification is not yet clear. Of note, these observations are confirmed in the *S. minutum* genome assembly; therefore it is unlikely that they are due to diversity of alternative transcript products in the transcriptome.

In contrast to the expansion of lysine and arginine methyltransferases, the number of proteins with classical “reader” domains is reduced in dinoflagellates, with the exception of the WD40 domain. Proteins with bromodomains, chromodomains, PHD fingers, Tudor or PWWP domains are found throughout the dinoflagellate phylogeny, but they are fewer in number compared to other protozoans, with the exception of dinotoms. Dinoflagellates do express a large number of proteins with WD40 domains; however, the WD40 domain is by no means restricted to the context of reading chromatin marks, and is found in many other proteins in the cell (Xu and Min 2011).

Finally, putative chromatin remodelers in dinoflagellates were identified. The number of candidate SWI/SNF ATPases is comparable to that in other protozoans, although clear CHD and ISWI homologs are detected in few species other than dinotoms (Figure 13).

### The FACT complex

**Figure 14:**
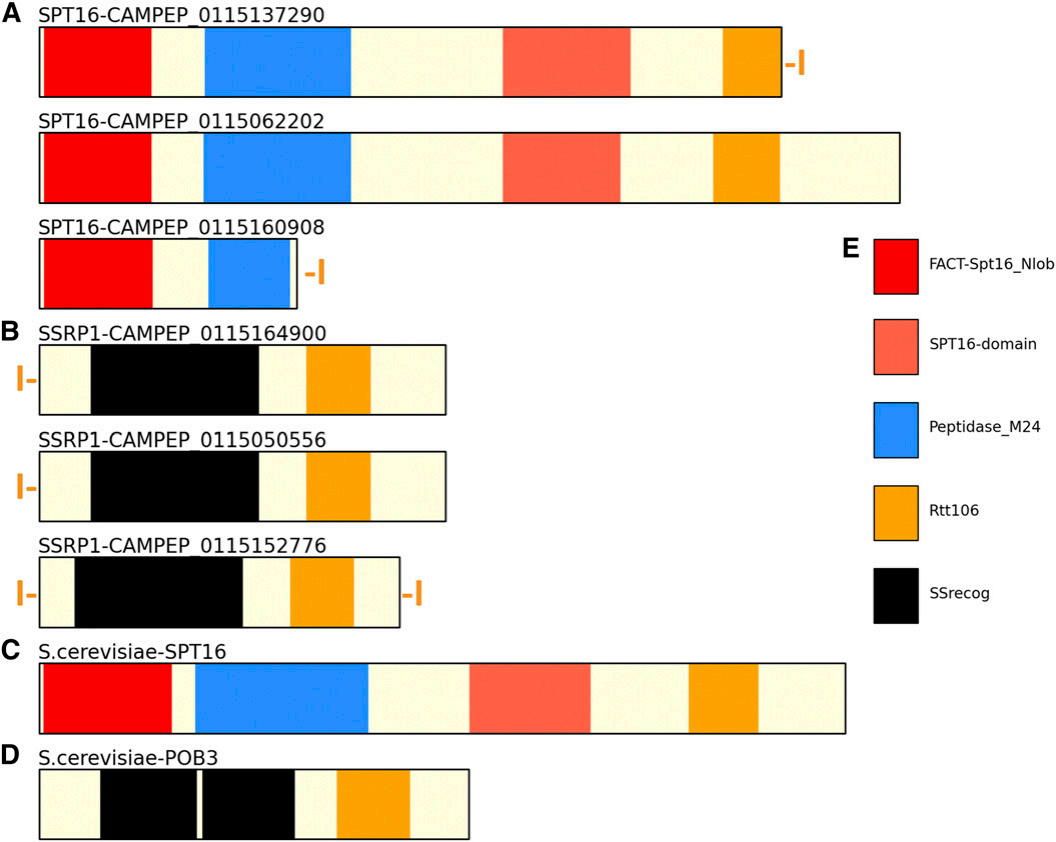
FACT complex subunits and their domain organization in *Polarella glacialis*. (A) *Polarella glacialis* SPT16 proteins; (B) *Polarella glacialis* SSRP1 proteins; (C) *Saccharomyces cerevisiae* SPT16; (D) *Saccharomyces cerevisiae* SSRP1 (POB3); (E) Domain color code. An orange “I” in front and/or after the protein indicates that the protein sequence is known to be not represented completely in the transcriptome assembly.

The FACT complex consists of two subunits, Spt16 and SSRP1, and plays a key role in transcription through chromatinized DNA templates (Orphanides *et al.* 1998; Reinberg and Sims 2006). Its presence or absence is therefore highly informative of the role that histones might play in dinoflagellates. Strikingly, homologs of the components of FACT are detected in almost all dinoflagellates (Figure 13), with an identical domain structure to that of FACT subunits in yeast and human (Figure 14), strongly implying that they indeed constitute a *bona fide* FACT complex. Furthermore, while the existence of multiple variants of each subunit in dinotoms is expected (Figure S12), a diversity of subunits is also observed in other species, as well as in *Perkinsus*. Intriguingly, in some cases variant SSRP1 subunits also contain HMG boxes, a DNA binding domain (Figure S13), which is not observed in FACT subunits of other eukaryotes; its functional significance is currently not clear.

The ubiquitous presence of the FACT complex throughout the dinoflagellate phylogeny suggests that transcription through nucleosomal arrays does occur in dinoflagellate nuclei.

### Histone chaperones

While the FACT complex disassembles and reassembles nucleosomes during transcription, other histone chaperones act during replication-dependent and replication-independent nucleosome assembly and histone exchange (Burgess and Zhang 2013). NAP1 loads H2A-H2B dimers (Mosammaparast *et al.* 2002), while CAF-1 is the replication-dependent and Hira the replication-independent chaperone for H3-H4 dimers (Verreault *et al.* 1996; Ray-Gallet *et al.* 2002); both receive the dimers from another loading factor, ASF1 (English *et al.* 2006).

Figure 13 shows the number of detected homologs for each factor. CAF-1 is only found in dinotoms and in *Perkinsus*, while Hira is detected in few but phylogenetically widely distributed dinoflagellate species. Given that ASF1 is found in almost all dinoflagellates, the absence of detection of Hira can be interpreted as a probable case of widespread false negatives. If, however, CAF-1 is genuinely absent in dinoflagellates (while Hira is present), that would have very intriguing implications for the role of nucleosomes in dinoflagellate chromatin (see *Discussion* section).

Remarkably, the majority of dinoflagellates express a large number of NAP1 proteins (Figure 13); this is also observed in a few other unicellular eukaryotes, but rarely to the same extent. One explanation for the expansion of the NAP1 family is that it might be due to an underlying diversification of NAP1 substrates, in the form of different H2A/H2B variants and dimers.

### RNA polymerase II CTD tails

The C-terminal domain of the largest subunit of RNA polymerase II (Rpb1) and its PTMS play a crucial role in coordinating numerous processes during the transcriptional cycle. In almost all eukaryotes (Yang and Stiller 2014) the tail consists of multiple copies of a consensus heptad sequence, YSPTSPS, which contains five phosphorylation sites and two proline isomerization sites. Specific modifications are deposited on and removed from these repeats during each phase of the transcriptional cycle, and they serve to recruit various effector and regulatory proteins (Buratowski 2009), constituting the so-called CTD code (Eick and Geyer 2013), operating in concert with the histone code.

Rpb1 homologs with CTD tails were identified in most dinoflagellates; curiously, in a few cases more than one homolog was found, even in nondinotom species. The heptad repeats are recognizable in most species (Figure 14); however, it is only in dinotom Rpb1 proteins that they closely match the consensus sequence (Figure S14B). In all other dinoflagellates, they are highly divergent (example shown in Figure S14A). This can be interpreted as a sign that some of the classical functionality of the CTD code has been lost in dinoflagellates, although divergent CTD repeats are seen in other eukaryotes too. The PTMs of the tails have not been directly studied in any such group, which will be necessary to clarify the roles they play in transcription.

## Discussion

This study presents the most comprehensive characterization of dinoflagellate histone proteins carried out so far. The presence of histones, despite their low abundance in dinoflagellate chromatin, is confirmed and generalized to all species for which genomic or transcriptomic sequence data are available. The properties of their sequences are integrated with the phylogenetic distribution of key components of the histone modification, chromatin remodeling, and transcriptional machineries. These analyses reveal that:

1. Core histones are present in all dinoflagellates, typically in multiple variants. The linker histone H1 is also detected in most species. Dinoflagellate histone variants include putative homologs, or at least functional analogs, of well-known variants in other eukaryotes, including H2A.Z, H2A.X, and possibly even H3.1/H3.3.
2. Overall, dinoflagellates express the most divergent histone proteins among all autonomous eukaryote nuclei: their histones exhibit frequent loss of key residues highly conserved among all other eukaryotes and many variants are elongated. The latter, however, is not a universal feature, as a fairly conventional set of histones (in terms of protein length, but not necessarily sequence conservation) is found in many species.
3. Dinoflagellate histones are expressed at lower levels than DVNPs, consistent with the limited role of histones in chromatin packaging.
4. The histone code exhibits significant divergence from the conventional eukaryote state but some of its key elements are conserved. These include H3K4 and some of the residues involved in transcription elongation. Histone marks associated with heterochromatin might also be conserved. The components of the histone code involved in chromatin dynamics during mitosis are among the least conserved.
5. Dinoflagellates express members of most protein families comprising the chromatin mark reader, writer, and eraser toolkit. Remarkably, lysine and arginine methyltransferases and demethylases have even expanded in the group. In contrast, the reader proteins display reduced diversity.
6. There is evidence for the presence of ATPases involved in chromatin remodeling in dinoflagellates.
7. Histone chaperones are definitely present and the NAP1 family has even expanded. It is possible that no replication-dependent H3-H4 chaperone is present, although that remains to be confirmed.
8. Remarkably, a complete FACT complex is also present, occasionally in multiple variants, meaning that transcription through nucleosome arrays occurs in dinoflagellates.
9. The RNA polymerase II CTD tails exhibit a high degree of divergence, making it unclear to what extent the CTD code is conserved.
10. Finally, *Perkinsus*, the sister lineage of dinoflagellates, has a mostly conventional set of histones, yet some of its histone variants exhibit divergence features that resemble what is observed in core dinoflagellates. This includes the otherwise most highly conserved and with very few variants histone H4. It is plausible that although perkinsid chromosomes are organized into typical nucleosomal chromatin, the first steps in the process of evolution toward the derived state seen in dinoflagellates might have taken place prior to the separation of the two groups.

How are we to interpret these observations? The presence of the FACT complex is key to the piecing together of a working model as it means that transcription through nucleosomes occurs in dinoflagellates. Less clear is the context in which it is happening. The permanent condensation and liquid crystalline state of dinoflagellate chromosomes is expected to not be particularly permissive to transcription. Thus, models of transcription in dinoflagellates have proposed that most of it happens on decondensed extrachromosomal chromatin loops that stick out of the permanently condensed general chromatin mass (Wisecaver and Hackett 2011). If that model is correct, it could be that these temporary loops associate with nucleosomes, and transcription proceeds in a more or less conventional manner, with promoters defined by the presence of H3K4me3 and maybe even H2A.Z, and a transcription elongation cycle involving the action of FACT and the deposition of H3K36me3 and some of the other marks usually associated with active transcription. The possible absence of replication-dependent histone chaperones also fits such a picture, as they would not be necessary if the primary mode of nucleosome assembly is related to active transcription outside of the S-phase (however, it is not directly compatible with the possible presence of replication-dependent and replication-independent H3.1/H3.3 variants). The observation that histone marks involved in chromatin condensation during mitosis are poorly conserved in dinoflagellates is also consistent with it.

Of course, many unknowns remain, for example, how the formation and identity of chromatin loops is regulated, which histones exactly associate with them, among the numerous variants observed, and what the role of the ones (if any) associating with other areas of the genome is. It is tempting and natural to think that the least derived histones are the ones forming nucleosomal arrays on loops.

This is the most plausible explanation, but it need not be true. Examples of significant innovation in the composition of the nucleosome are known; for example, in bdelloid rotifers, a metazoan lineage with otherwise normal chromatin, conventional histone H2A has apparently been completely replaced by an elongated H2A variant (Van Doninck *et al.* 2009). Given the uniqueness and extreme divergence of dinoflagellate nuclear organization, the possibility of analogous innovations cannot be rejected, including some that are restricted to individual lineages and not common to all dinoflagellates.

Additional clues might be provided by sperm cells, the other notable example of nuclei in which histones do not play a primary packaging role, a system that has been studied much more extensively. Chromatin undergoes a dramatic transformation during spermatogenesis as histones are largely replaced by protamines and it becomes condensed (Rathke *et al.* 2014), a condition reminiscent of that of dinoflagellate nuclei. However, not all histones are removed – recent genome-wide mapping studies have revealed that they remain associated with a small fraction of the genome (Hammoud *et al.* 2009; Erkek *et al.* 2013; Carone *et al.* 2014), in particular the promoters of developmental regulators. Of note, it seems that nucleosomal patterns upon nuclease digestion of sperm chromatin only become apparent under preparation conditions that stabilize protein–DNA interactions (Carone *et al.* 2014). Sperm is also the site of expression of many unique histone variants (Wiedemann *et al.* 2010; Soboleva *et al.* 2011; Schenk *et al.* 2011), thought to play a role in both the process of histone replacement and in the final condensed chromatin. Parallels can be drawn between that system and dinoflagellates: variant histones might be associated with specific regions of dinoflagellate genomes even within an otherwise permanently condensed chromatin mass.

Specialized histone variants from other organisms might also have analogs in dinoflagellates, and these are not limited to centromeric histones. The group that might be most informative is the kinetoplastids. Dinoflagellates and kinetoplastids have evolved convergently in many aspects of their biology (Lukes *et al.* 2009), including the polycistronic organization of genes and the ubiquity of *trans*-splicing. Histone marks and variants have been studied in some kinetoplastids and intriguing discoveries have been made. For example, in *Trypanosoma brucei* novel variants of all four core histones mark the boundaries of polycistronic transcription units (Siegel *et al.* 2009), and another histone H3 variant is associated with telomeres (Lowell and Cross 2004). Analogous variants might be present in dinoflagellates. Chromosome dynamics during mitosis in kinetoplastids is also unique and apparently either highly derived or very deeply diverging (Akiyoshi and Gull 2013); trypanosomatid H3 histones lack H3S10 (Sullivan *et al.* 2006), which might be related to this fact, and has similarities to the poor conservation of histone phosphorylation sites in dinoflagellates.

The restriction of histones to certain sections of the genome possibly combined with their diversification and subfunctionalization can explain the relaxation of the constraints on their sequence imposed by the histone code. The functional role of DVNPs is highly relevant for clarifying to what extent that hypothesis is true. The focus of this study has been on histones, but DVNPs and any other histone-like proteins in dinoflagellates are no less interesting. They could well function as much more than mere packaging proteins and be involved in a completely new “DVNP code”, analogous to the histone code. Not only that but a large number of distinct DVNPs are expressed in all dinoflagellates; the divisions of functions between them are completely unknown at present. A separate DVNP code might provide an explanation for the expansion of some portions of the epigenetic writer and eraser toolkit, as DVNPs are most likely methylated by novel, specialized KMT enzymes. Less clear in such context is why proteins with classical reader domains are reduced in number. Some possible ways out of this conundrum include the takeover of such functionalities by proteins with other domains (such as WD40) and the evolution of entirely novel ones.

DVNPs and permanent condensation of chromatin could also explain the poor conservation of histone phosphorylation during the mitotic cycle – dinoflagellate histones are not involved in a nucleosomal compaction and decompaction cycle during mitosis. Similarly, DVNPs are likely to be the main component of what is the equivalent of heterochromatin, which, if true, makes it unclear what functional roles might be left for histone-based heterochromatinization; this is the reason why the putative conservation of heterochromatin marks is the most uncertain among the modifications discussed here.

An enormous amount remains to be learned about dinoflagellate chromatin, transcription, and transcriptional and post-transcriptional regulation. The answers to these questions will derive from the application of the genomics and proteomics tools that have successfully been applied to the study of chromatin structure and histone modifications in model eukaryote systems. These include the use of targeted mass-spectrometry analysis to reveal the exact PTMs deposited *in vivo* onto histones and DVNPs (Tan *et al.* 2011), and of functional genomic assays such as ChIP-seq (Johnson *et al.* 2007), ATAC-seq (Buenrostro *et al.* 2013) and DNAse-seq (Hesselberth *et al.* 2009), and Hi-C/ChIA-PET (Lieberman-Aiden *et al.* 2009; Fullwood *et al.* 2009) to characterize chromatin structure, the distribution of histones, histone marks, and other chromatin-associated proteins along dinoflagellate genomes, and their three-dimensional organization. A prerequisite for such studies is the availability of high-quality and reasonably complete genome sequences from several species, which should be facilitated by the ongoing advances in genome sequencing technologies.

## Supplementary Material

Supporting Information
