# Supplementary material for: Diversity and Divergence of Dinoflagellate Histone Proteins
Source: G3 (Bethesda). 2015 Dec 8;6(2):397–422. doi: 10.1534/g3.115.023275 (PMC4751559; doi:10.1534/g3.115.023275)
Supplement: Supporting Information [file supp_6_2_397__index.html]

Diversity and Divergence of Dinoflagellate Histone Proteins — Diversity and Divergence of Dinoflagellate Histone Proteins — Supporting Information 

# Diversity and Divergence of Dinoflagellate Histone Proteins

## Supporting Information for Marinov and Lynch, 2016

**Files in this Data Supplement:**

- Supporting Information - File contains supporting figures and tables. (.pdf, 4,754 KB)
- Figure S9 - Known histone modifications in vertebrates. (.pdf, 281 KB)
- Figure S10 - Multiple sequences alignments of core histones H3 sequences and centromeric H3 variants in several eukaryotes. (.pdf, 883 KB)
- Figure S11 - Multiple sequences alignments of histones H3 sequences from *Alexandrium monilatum* and histone H3.1 from *Homo sapiens*. (.pdf, 488 KB)
- Figure S12 - FACT complex subunits and their domain organization in *Durinskia baltica*. (.pdf, 250 KB)
- Figure S13 - FACT complex subunits and their domain organization in *Alexandrium tamarense*. (.pdf, 259 KB)
- Figure S14 - RNA Polymerase II largest subunit CTD repeats in dinofagellates. (.pdf, 802 KB)
- Table S1 - Summary of MMETSP samples used in this study. (.pdf, 94 KB)
- Table S2 - Putative H2A.X histone variants in dinofagellates. (.pdf, 114 KB)
- Figure S1 - Protein domains in dinofagellate histones. (.pdf, 219 KB)
- Figure S2 - Expression levels of DVNP, linker histone and histone genes in dinofagellates. (.pdf, 288 KB)
- Figure S3 - Expression levels of DVNP, linker histone and histone genes in dinofagellates. (.pdf, 301 KB)
- Figure S4 - Expression levels of DVNP, linker histone and histone genes in dinofagellates. (.pdf, 318 KB)
- Figure S5 - Expression levels of DVNP, linker histone and histone genes in dinofagellates. (.pdf, 304 KB)
- Figure S6 - Expression levels of DVNP, linker histone and histone genes in dinofagellates. (.pdf, 219 KB)
- Figure S7 - Expression levels of DVNP, linker histone and histone genes in dinofagellates. (.pdf, 242 KB)
- Figure S8 - Putative H3.3/H3.1 histone variants in dinofagellates. (.pdf, 692 KB)
