# Supplementary material for: Diversity and Divergence of Dinoflagellate Histone Proteins
Source: G3 (Bethesda). 2015 Dec 8;6(2):397–422. doi: 10.1534/g3.115.023275 (PMC4751559; doi:10.1534/g3.115.023275)
Supplement: Supporting Information [file supp_g3.115.023275_023275SI.pdf]

# Supplementary Materials

## Supplementary Tables

**Table S1: Summary of MMETSP samples used in this study**. The table is based in information provided by the MMETSP project page.

| Sample name | Group | Family | Species | Strain | Clonal | Axenic |
|---|---|---|---|---|---|---|
| MMETSP0093 | Dinoflagellata | Goniodomataceae | *Alexandrium monilatum* | CCMP3105 | 1 | No |
| MMETSP0095 | Dinoflagellata | Goniodomataceae | *Alexandrium monilatum* | CCMP3105 | 1 | No |
| MMETSP0096 | Dinoflagellata | Goniodomataceae | *Alexandrium monilatum* | CCMP3105 | 1 | No |
| MMETSP0097 | Dinoflagellata | Goniodomataceae | *Alexandrium monilatum* | CCMP3105 | 1 | No |
| MMETSP0378 | Dinoflagellata | Goniodomataceae | *Alexandrium tamarense* | CCMP1771 | 1 | 1 |
| MMETSP0380 | Dinoflagellata | Goniodomataceae | *Alexandrium tamarense* | CCMP1771 | 1 | 1 |
| MMETSP0382 | Dinoflagellata | Goniodomataceae | *Alexandrium tamarense* | CCMP1771 | 1 | 1 |
| MMETSP0384 | Dinoflagellata | Goniodomataceae | *Alexandrium tamarense* | CCMP1771 | 1 | 1 |
| MMETSP0795 | Dinoflagellata | Goniodomaceae | *Amoebophrya* sp. | Ameob2 | 1 | No |
| MMETSP0258 | Dinoflagellata | Gymnodiniaceae | *Amphidinium carterae* | CCMP1314 | Unknown | No |
| MMETSP0259 | Dinoflagellata | Gymnodiniaceae | *Amphidinium carterae* | CCMP1314 | Unknown | No |
| MMETSP0398C | Dinoflagellata | Gymnodiniaceae | *Amphidinium carterae* | CCMP1314 | 1 | No |
| MMETSP0399 | Dinoflagellata | Gymnodiniaceae | *Amphidinium carterae* | CCMP1314 | 1 | |
| MMETSP1036 | Dinoflagellata | Unknown | *Azadinium spinosum* | 3D9 | 1 | 1 |
| MMETSP1037 | Dinoflagellata | Unknown | *Azadinium spinosum* | 3D9 | 1 | 1 |
| MMETSP1038 | Dinoflagellata | Unknown | *Azadinium spinosum* | 3D9 | 1 | 1 |
| MMETSP1462 | Dinoflagellata | Peridiniaceae | *Brandtodinium nutriculum* | RCC3387 | 1 | No |
| MMETSP1074 | Dinoflagellata | Ceratiaceae | *Ceratium fusus* | PA161109 | 1 | No |
| MMETSP1075 | Dinoflagellata | Ceratiaceae | *Ceratium fusus* | PA161109 | 1 | No |
| MMETSP0323 | Dinoflagellata | Crypthecodiniacea | *Crypthecodinium cohnii* | Seligo | 1 | 1 |
| MMETSP0324 | Dinoflagellata | Crypthecodiniacea | *Crypthecodinium cohnii* | Seligo | 1 | 1 |
| MMETSP0325 | Dinoflagellata | Crypthecodiniacea | *Crypthecodinium cohnii* | Seligo | 1 | 1 |
| MMETSP0326 | Dinoflagellata | Crypthecodiniacea | *Crypthecodinium cohnii* | Seligo | 1 | 1 |
| MMETSP0797 | Dinoflagellata | Dinophysiaceae | *Dinophysis acuminata* | DAEP01 | Unknown | No |
| MMETSP0116 | Dinoflagellata | Peridiniaceae | *Durinskia baltica* | CSIRO CS-38 | No | No |
| MMETSP0117 | Dinoflagellata | Peridiniaceae | *Durinskia baltica* | CSIRO CS-38 | No | No |
| MMETSP0766 | Dinoflagellata | Goniodomaceae | *Gambierdiscus australes* | CAWD 149 | 1 | No |
| MMETSP0118 | Dinoflagellata | Peridiniaceae | *Glenodinium foliaceum* | CCAP 1116/3 | No | No |
| MMETSP0119 | Dinoflagellata | Peridiniaceae | *Glenodinium foliaceum* | CCAP 1116/3 | No | No |
| MMETSP1439 | Dinoflagellata | Gonyaulacaceae | *Gonyaulax spinifera* | CCMP409 | Unknown | No |
| MMETSP0784 | Dinoflagellata | Gymnodiniaceae | *Gymnodinium catenatum* | GC744 | 1 | No |
| MMETSP1148 | Dinoflagellata | Gymnodiniaceae | *Gyrodinium dominans* | SPMC 103 | No | No |
| MMETSP0503 | Dinoflagellata | Heterocapsaceae | *Heterocapsa rotundata* | SCCAP K-0483 | No | No |
| MMETSP0448 | Dinoflagellata | Heterocapsaceae | *Heterocapsa triquestra* | CCMP 448 | 1 | No |
| MMETSP0027 | Dinoflagellata | Gymnodiniaceae | *Karenia brevis* | CCMP2229 | 1 | No |
| MMETSP0029 | Dinoflagellata | Gymnodiniaceae | *Karenia brevis* | CCMP2229 | 1 | No |
| MMETSP0030 | Dinoflagellata | Gymnodiniaceae | *Karenia brevis* | CCMP2229 | 1 | No |
| MMETSP0031 | Dinoflagellata | Gymnodiniaceae | *Karenia brevis* | CCMP2229 | 1 | No |
| MMETSP0201 | Dinoflagellata | Gymnodiniaceae | *Karenia brevis* | Wilson | Unknown | No |
| MMETSP0202 | Dinoflagellata | Gymnodiniaceae | *Karenia brevis* | Wilson | Unknown | No |
| MMETSP0527 | Dinoflagellata | Gymnodiniaceae | *Karenia brevis* | SP3 | 1 | No |
| MMETSP0528 | Dinoflagellata | Gymnodiniaceae | *Karenia brevis* | SP3 | 1 | No |
| MMETSP0573 | Dinoflagellata | Gymnodiniaceae | *Karenia brevis* | SP1 | 1 | No |
| MMETSP0574 | Dinoflagellata | Gymnodiniaceae | *Karenia brevis* | SP1 | 1 | No |
| MMETSP0648 | Dinoflagellata | Gymnodiniaceae | *Karenia brevis* | Wilson | 1 | No |
| MMETSP0649 | Dinoflagellata | Gymnodiniaceae | *Karenia brevis* | Wilson | 1 | No |
| MMETSP1015 | Dinoflagellata | Gymnodiniaceae | *Karlodinium micrum* | CCMP2283 | Unknown | No |
| MMETSP1016 | Dinoflagellata | Gymnodiniaceae | *Karlodinium micrum* | CCMP2283 | Unknown | No |
| MMETSP1017 | Dinoflagellata | Gymnodiniaceae | *Karlodinium micrum* | CCMP2283 | Unknown | No |
| MMETSP0120 | Dinoflagellata | Peridiniaceae | *Kryptoperidinium foliaceum* | CCMP 1326 | No | No |
| MMETSP0121 | Dinoflagellata | Peridiniaceae | *Kryptoperidinium foliaceum* | CCMP 1326 | No | No |

*Continued on next page*

Table S1 – *Continued from previous page*

| Sample name | Group | Family | Species | Strain | Clonal | Axenic |
|---|---|---|---|---|---|---|
| MMETSP1032 | Dinoflagellata | Gonyaulacaceae | *Lingulodinium polyedra* | CCMP 1738 | 1 | No |
| MMETSP1033 | Dinoflagellata | Gonyaulacaceae | *Lingulodinium polyedra* | CCMP 1738 | 1 | No |
| MMETSP1034 | Dinoflagellata | Gonyaulacaceae | *Lingulodinium polyedra* | CCMP 1738 | 1 | No |
| MMETSP1035 | Dinoflagellata | Gonyaulacaceae | *Lingulodinium polyedra* | CCMP 1738 | 1 | No |
| MMETSP0253 | Dinoflagellata | Noctilucaceae | *Noctiluca scintillans* | | No | No |
| MMETSP0468 | Dinoflagellata | Oxyrrhinaceae | *Oxyrrhis marina* | | No | No |
| MMETSP0469 | Dinoflagellata | Oxyrrhinaceae | *Oxyrrhis marina* | | No | No |
| MMETSP0470 | Dinoflagellata | Oxyrrhinaceae | *Oxyrrhis marina* | | No | No |
| MMETSP0471 | Dinoflagellata | Oxyrrhinaceae | *Oxyrrhis marina* | | No | No |
| MMETSP1424 | Dinoflagellata | Oxyrrhinaceae | *Oxyrrhis marina* | LB1974 | No | No |
| MMETSP1425 | Dinoflagellata | Oxyrrhinaceae | *Oxyrrhis marina* | LB1974 | No | No |
| MMETSP1426 | Dinoflagellata | Oxyrrhinaceae | *Oxyrrhis marina* | LB1974 | No | No |
| MMETSP1338 | Dinoflagellata | Suessiaceae | *Pelagodinium beii* | RCC1491 | | |
| MMETSP0370 | Dinoflagellata | Peridiniaceae | *Peridinium aciculiferum* | PAER-2 | 1 | No |
| MMETSP0371 | Dinoflagellata | Peridiniaceae | *Peridinium aciculiferum* | PAER-2 | 1 | No |
| MMETSP1440 | Dinoflagellata | Suessiaceae | *Polarella glacialis* | CCMP2088 | 1 | No |
| MMETSP0227 | Dinoflagellata | Suessiaceae | *Polarella glacialis* | CCMP 1383 | Unknown | No |
| MMETSP0053 | Dinoflagellata | Prorocentraceae | *Prorocentrum minimum* | CCMP1329 | | 1 |
| MMETSP0055 | Dinoflagellata | Prorocentraceae | *Prorocentrum minimum* | CCMP1329 | | 1 |
| MMETSP0056 | Dinoflagellata | Prorocentraceae | *Prorocentrum minimum* | CCMP1329 | | 1 |
| MMETSP0057 | Dinoflagellata | Prorocentraceae | *Prorocentrum minimum* | CCMP1329 | | 1 |
| MMETSP0267 | Dinoflagellata | Prorocentraceae | *Prorocentrum minimum* | CCMP2233 | Unknown | No |
| MMETSP0268 | Dinoflagellata | Prorocentraceae | *Prorocentrum minimum* | CCMP2233 | Unknown | No |
| MMETSP0269 | Dinoflagellata | Prorocentraceae | *Prorocentrum minimum* | CCMP2233 | Unknown | No |
| MMETSP0228 | Dinoflagellata | Gonyaulacaceae | *Protoceratium reticulatum* | CCCM535 (=CCMP1889) | Unknown | No |
| MMETSP0796 | Dinoflagellata | Gonyaulacaceae | *Pyrodinium bahamense* | pbaha01 | 1 | No |
| MMETSP0359 | Dinoflagellata | Peridiniaceae | *Scrippsiella hangoei* | SHTV-5 | 1 | No |
| MMETSP0360 | Dinoflagellata | Peridiniaceae | *Scrippsiella hangoei* | SHTV-5 | 1 | No |
| MMETSP0361 | Dinoflagellata | Peridiniaceae | *Scrippsiella hangoei* | SHTV-5 | 1 | No |
| MMETSP0367 | Dinoflagellata | Peridiniaceae | *Scrippsiella hangoei*-like | SHHI-4 | 1 | No |
| MMETSP0368 | Dinoflagellata | Peridiniaceae | *Scrippsiella hangoei*-like | SHHI-4 | 1 | No |
| MMETSP0369 | Dinoflagellata | Peridiniaceae | *Scrippsiella hangoei*-like | SHHI-4 | 1 | No |
| MMETSP0270 | Dinoflagellata | Peridiniaceae | *Scrippsiella trochoidea* | CCMP3099 | No | No |
| MMETSP0271 | Dinoflagellata | Peridiniaceae | *Scrippsiella trochoidea* | CCMP3099 | No | No |
| MMETSP0272 | Dinoflagellata | Peridiniaceae | *Scrippsiella trochoidea* | CCMP3099 | No | No |
| MMETSP1115 | Dinoflagellata | Symbiodiniaceae | *Symbiodinium* sp. | CCMP2430 | No | No |
| MMETSP1116 | Dinoflagellata | Symbiodiniaceae | *Symbiodinium* sp. | CCMP2430 | No | No |
| MMETSP1117 | Dinoflagellata | Symbiodiniaceae | *Symbiodinium* sp. | CCMP2430 | No | No |
| MMETSP1122 | Dinoflagellata | Symbiodiniaceae | *Symbiodinium* sp. | Mp | No | No |
| MMETSP1123 | Dinoflagellata | Symbiodiniaceae | *Symbiodinium* sp. | Mp | No | No |
| MMETSP1124 | Dinoflagellata | Symbiodiniaceae | *Symbiodinium* sp. | Mp | No | No |
| MMETSP1125 | Dinoflagellata | Symbiodiniaceae | *Symbiodinium* sp. | Mp | No | No |
| MMETSP1367 | Dinoflagellata | Symbiodiniaceae | *Symbiodinium* sp. | C1 | Unknown | No |
| MMETSP1369 | Dinoflagellata | Symbiodiniaceae | *Symbiodinium* sp. | C1 | Unknown | No |
| MMETSP1370 | Dinoflagellata | Symbiodiniaceae | *Symbiodinium* sp. | C15 | Unknown | No |
| MMETSP1371 | Dinoflagellata | Symbiodiniaceae | *Symbiodinium* sp. | C15 | Unknown | No |
| MMETSP0224 | Dinoflagellata | Gymnodiniaceae | *Togula jolla* | CCCM 725 | Unknown | No |
| MMETSP0924C | Perkinsida | Perkinsidae | *Perkinsus chesapeaki* | ATCC PRA-65 | 1 | No |
| MMETSP0925 | Perkinsida | Perkinsidae | *Perkinsus chesapeaki* | ATCC PRA-65 | 1 | |
| MMETSP0922 | Perkinsida | Perkinsidae | *Perkinsus marinus* | ATCC 50439 | 1 | No |
| MMETSP0923 | Perkinsida | Perkinsidae | *Perkinsus marinus* | ATCC 50439 | 1 | |
| MMETSP0290 | Chromerida | Unknown | *Chromera velia* | CCMP2878 | 1 | 1 |

**Table S2: Putative H2A.X histone variants in dinoflagellates**. The H2A.X variants of histone H2A are characterized by the presence of a SQ(E/D)Φ phosphorylation motif at the C-terminus of the protein (Talbert et al. 2012). Note that the motif is usually SQDY in heterokonts (Talbert et al. 2012), which include diatoms and thus the endosymbiont of dinotoms, thus one of proteins listed below in *Durinskia baltica* is most likely to be of endosymbiont origin.

| Species | Protein | Length | C-terminal sequence |
|---|---|---|---|
| *Perkinsus marinus* | EER08766.1 | 137 | **SQEM** |
| *Perkinsus marinus* | EER09215.1 | 135 | **SQEM** |
| *Perkinsus marinus* | EER15538.1 | 162 | **SQEM** |
| *Perkinsus marinus* | EER15802.1 | 136 | **SQEI** |
| *Perkinsus marinus* | EEQ99722.1 | 155 | **SQEM** |
| *Perkinsus marinus* | EER04007.1 | 164 | **SQEM** |
| *Perkinsus marinus* | EER04402.1 | 164 | **SQEM** |
| *Perkinsus marinus* | EEQ98671.1 | 138 | **SQEM** |
| *Perkinsus marinus* | EEQ97488.1 | 92 | **SQEM** |
| *Symbiodinium* sp. C15 | CAMPEP_0192465542 | 177 | **SQEY** |
| *Symbiodinium* sp. C1 | CAMPEP_0199619000 | 181 | **SQEY** |
| *Symbiodinium* sp. C1 | CAMPEP_0199597416 | 160 | **SQEY** |
| *Scrippsiella trochoidea* | CAMPEP_0192083196 | 204 | **SQEY** |
| *Polarella glacialis* | CAMPEP_0115091146 | 166 | **SQEY** |
| *Pelagodinium beii* | CAMPEP_0197627280 | 157 | **SQEY** |
| *Oxyrrhis marina* LB1974 | CAMPEP_0190412876 | 136 | **SQQY** |
| *Oxyrrhis marina* | CAMPEP_0190349664 | 136 | **SQQY** |
| *Noctiluca scintillans* | CAMPEP_0194480802 | 179 | **SQEF** |
| *Kryptoperidinium foliaceum* | CAMPEP_0189651904 | 130 | **SQEF** |
| *Karlodinium micrum* | CAMPEP_0200762398 | 199 | **SQEF** |
| *Glenodinium foliaceum* | CAMPEP_0188370172 | 132 | **SQEF** |
| *Durinskia baltica* | CAMPEP_0200040914 | 137 | **SQDF** |
| *Durinskia baltica* | CAMPEP_0200047580 | 153 | **SQDY** |
| *Crypthecodinium cohnii* | CAMPEP_0193858338 | 196 | **SQEF** |
| *Crypthecodinium cohnii* | CAMPEP_0193883494 | 196 | **SQEF** |
| *Alexandrium tamarense* | CAMPEP_0186381488 | 131 | **SQSY** |
| *Alexandrium tamarense* | CAMPEP_0186337128 | 141 | **SQEY** |
| *Alexandrium monilatum* | CAMPEP_0200550256 | 187 | **SQEF** |
| *Symbiodinium minutum* | symbB.v1.2.004801.t1 | 177 | **SQEY** |

# Supplementary Figures

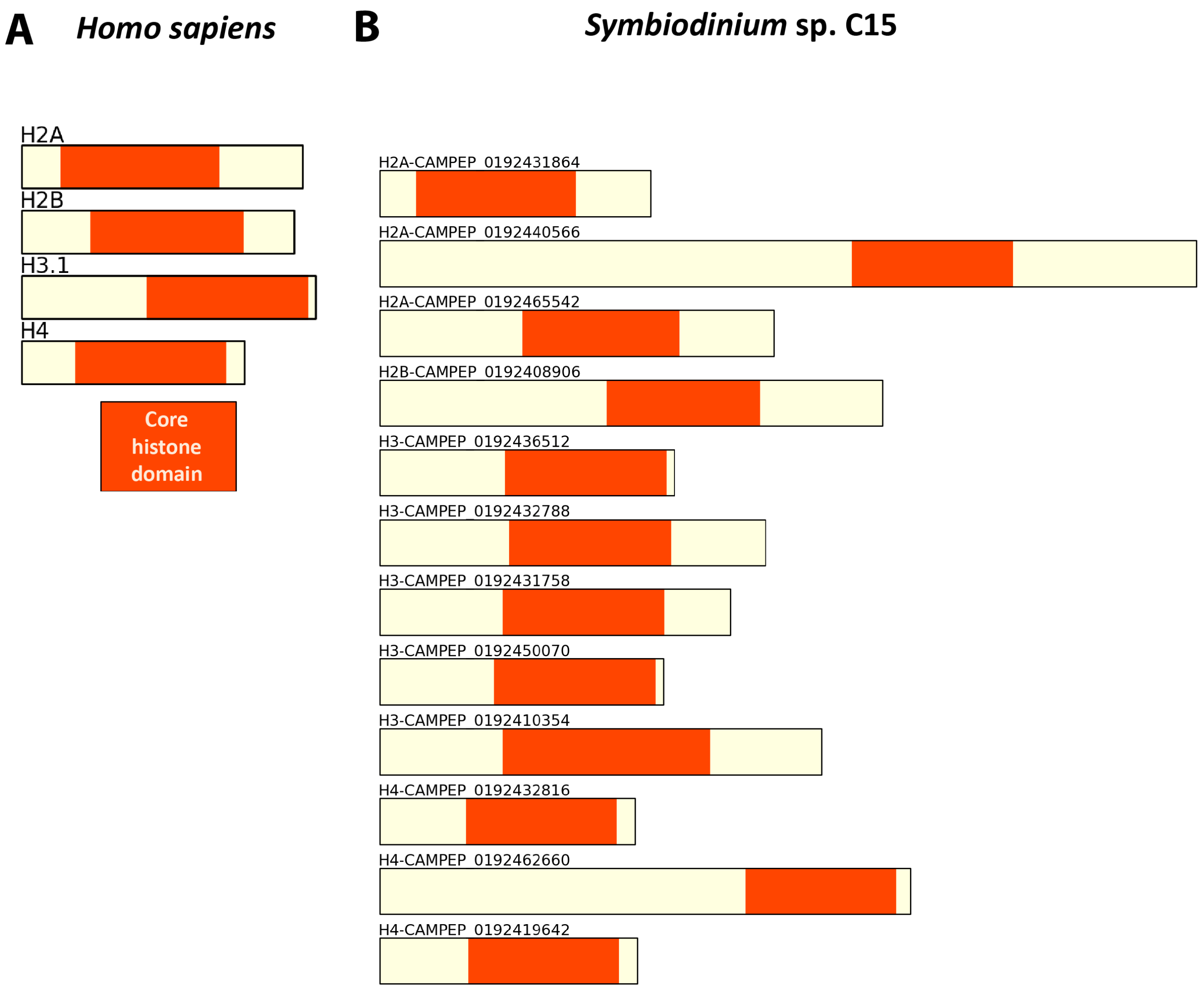


**Figure S1: Protein domains in dinoflagellate histones**. (A) *Homo sapiens* histones, shown for reference; (B) *Symbiodinium* sp. C15 histones.

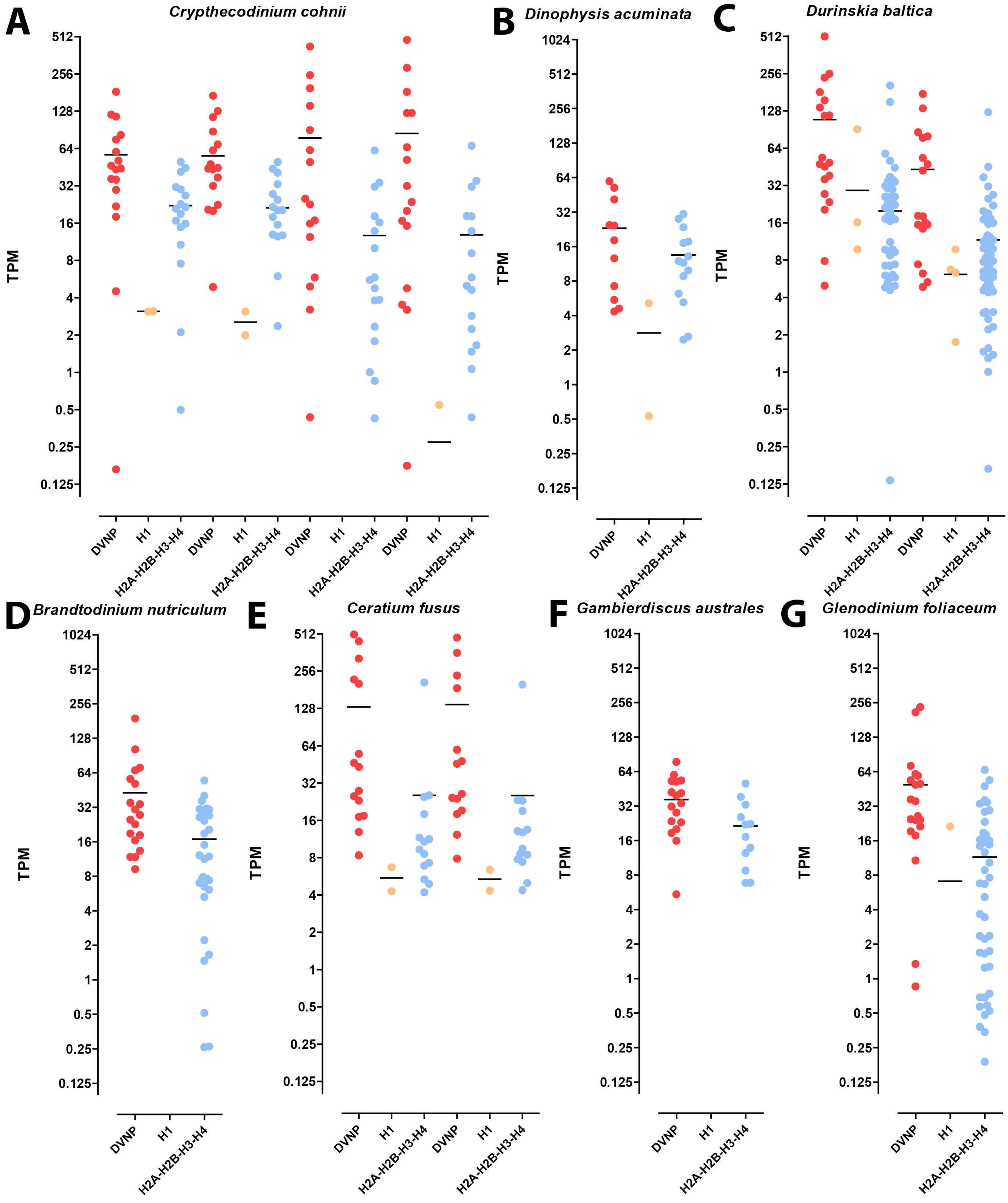


**Figure S2: Expression levels of DVNP, linker histone and histone genes in dinoflagellates.** (A) *Crypthecodinium cohnii*; from left to right: SRR1296889, SRR1296890, SRR1296960, SRR1296961; (B) *Dinophysis acuminata*; SRR1296701; (C) *Durinskia baltica*; from left to right: SRR1296839, SRR1296941; (D) *Brandtodinium nutriculum*: SRR1300537; (E) *Ceratium fusus*: from left to right: SRR1300300, SRR1300301; (F) *Gambierdiscus australes*: SRR1296893; (G) *Glenodinium foliaceum*: SRR1296842.

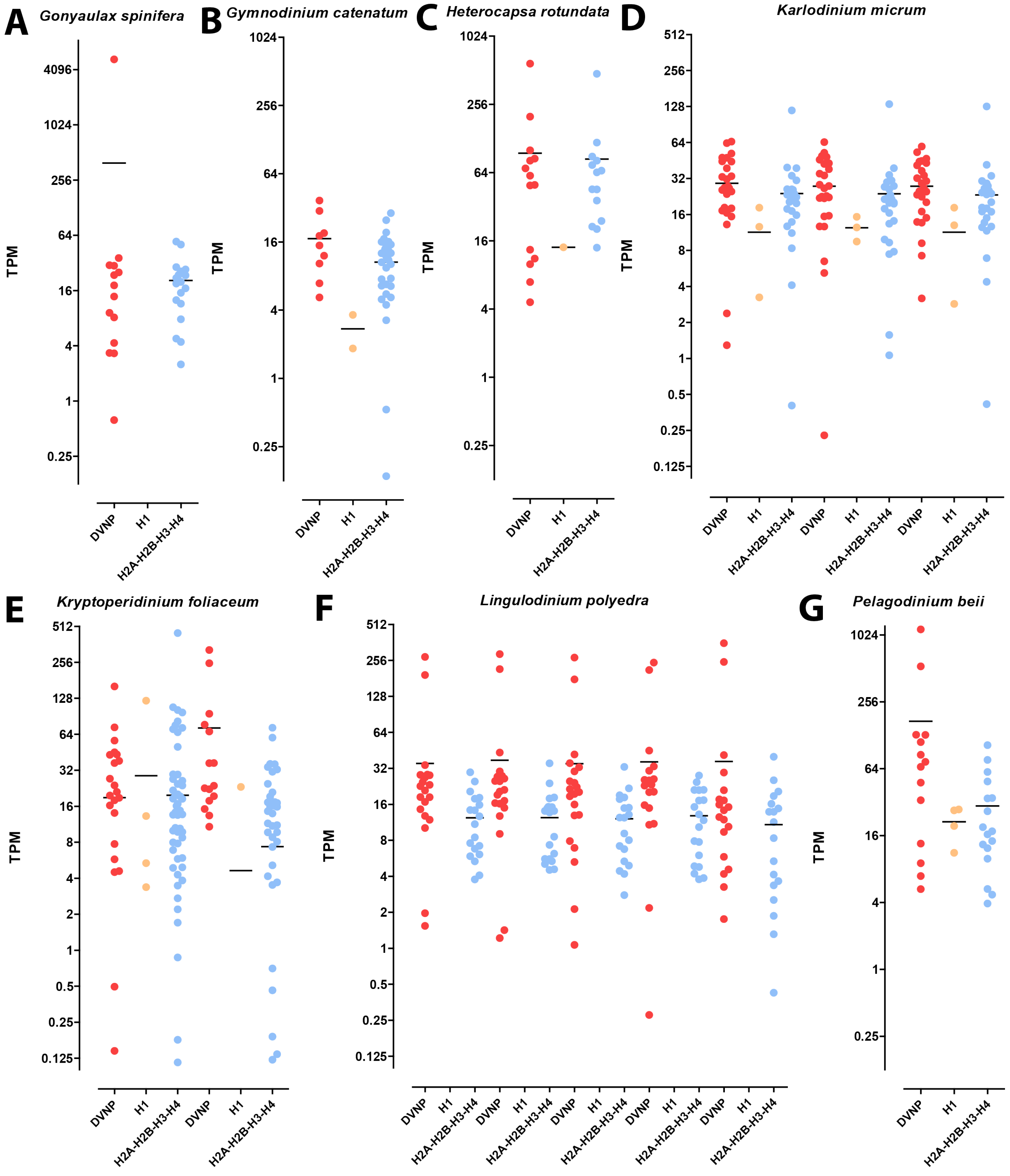


**Figure S3: Expression levels of DVNP, linker histone and histone genes in dinoflagellates**. (A) *Gonyaulax spinifera*: SRR1300518; (B) *Gymnodinium catenatum*: SRR1296705; (C) *Heterocapsa rotundata*: SRR1296810; (D) *Karlodinium micrum* CCMP2283; from left to right: SRR1300325, SRR1300326, SRR1300327; (E) *Kryptoperidinium foliaceum*; from left to right: SRR1296841, SRR1296842; (F) *Lingulodinium polyedra*; from left to right: SRR1300255, SRR1300256, SRR1300257, SRR1300258, SRR584359; (G) *Pelagodinium beii*: SRR1300503.

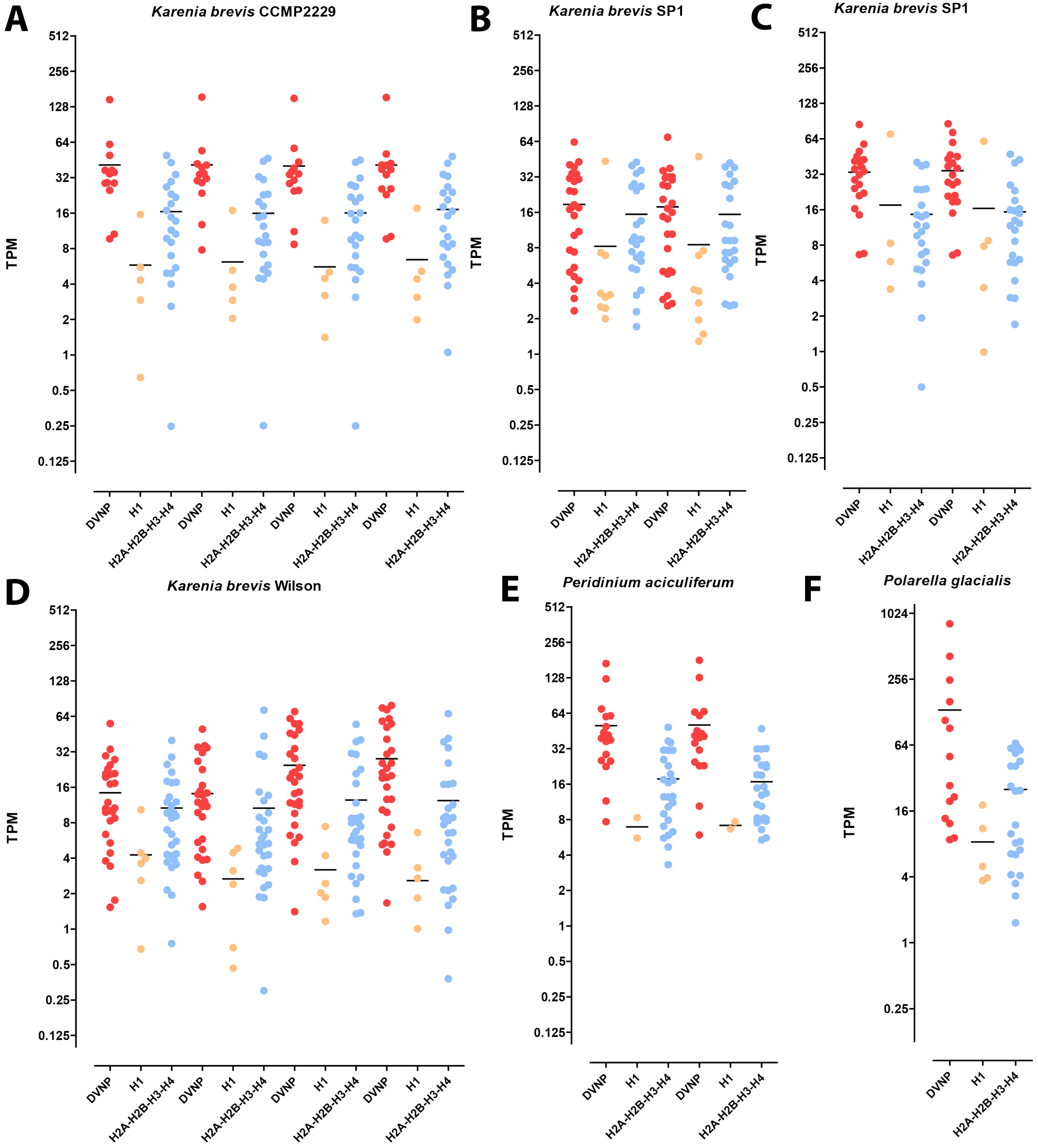


**Figure S4: Expression levels of DVNP, linker histone and histone genes in dinoflagellates**. (A) *Karenia brevis* CCMP2229; from left to right: SRR1296748, SRR1296749, SRR1296750, SRR1296952; (B) *Karenia brevis* SP1; from left to right: SRR1296712, SRR1296714; (C) *Karenia brevis* SP3; from left to right: SRR1163514, SRR1163516; (D) *Karenia brevis* Wilson; from left to right: SRR1296743, SRR1296744, SRR1296853, SRR1296854; (E) *Peridinium aciculiferum*; from left to right: SRR1294439, SRR1294440; (F) *Polarella glacialis*; SRR1296751.

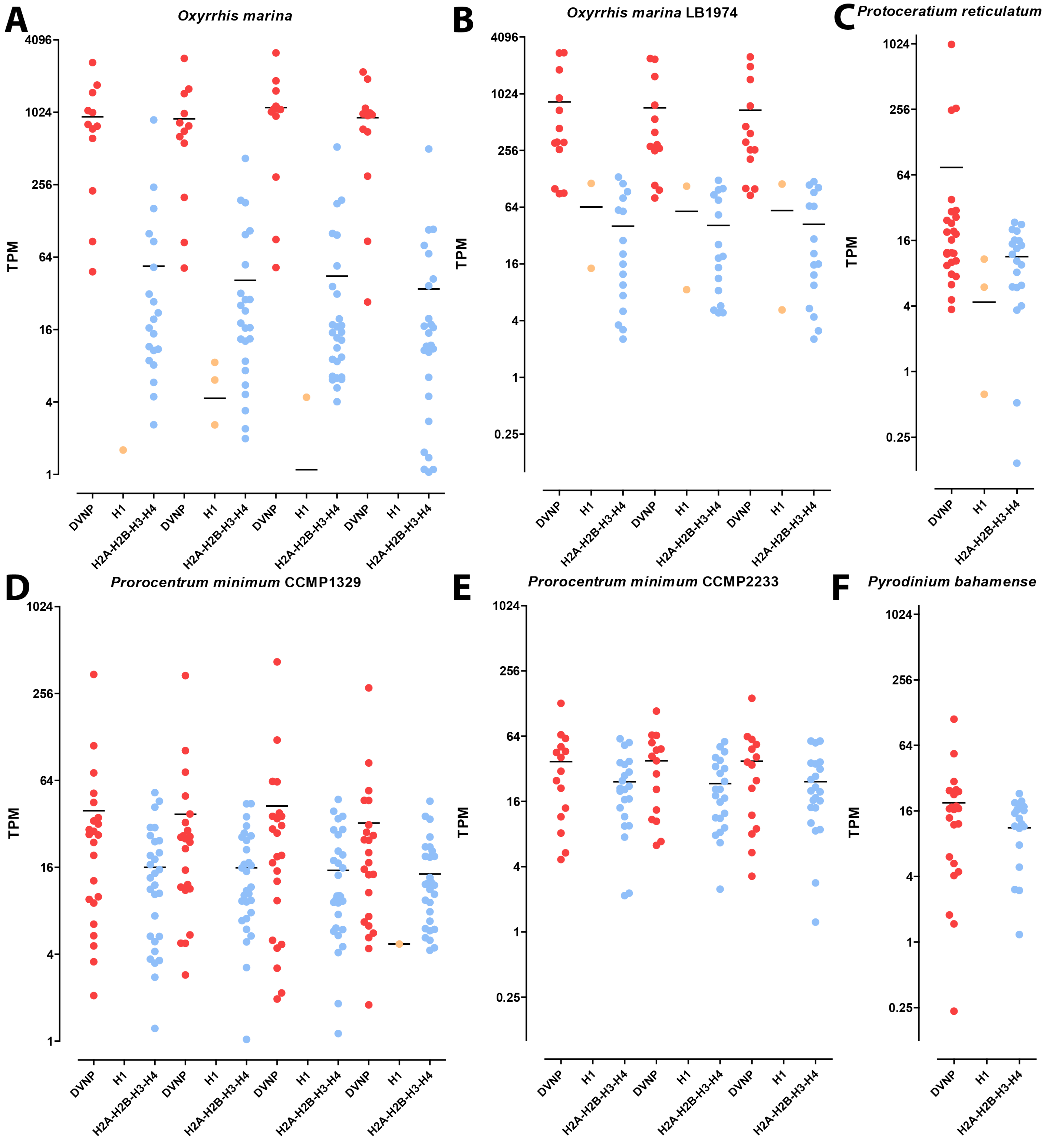


**Figure S5: Expression levels of DVNP, linker histone and histone genes in dinoflagellates**. (A) *Oxyrrhis marina*; from left to right: SRR1296900, SRR1296901, SRR1296903, SRR1296907; (B) *Oxyrrhis marina* LB1974; from left to right: SRR1300472, SRR1300473, SRR1300474; (C) *Protoceratium reticulatum*: SRR1296738; (D) *Prorocentrum minimum* CCMP1329; from left to right: SRR1296784, SRR1296785, SRR1296787, SRR1296788; (E) *Protoceratium reticulatum* CCMP2233; from left to right: SRR1296752, SRR1296753, SRR1296754; (F) *Pyrodinium bahamense*: SRR1296702.

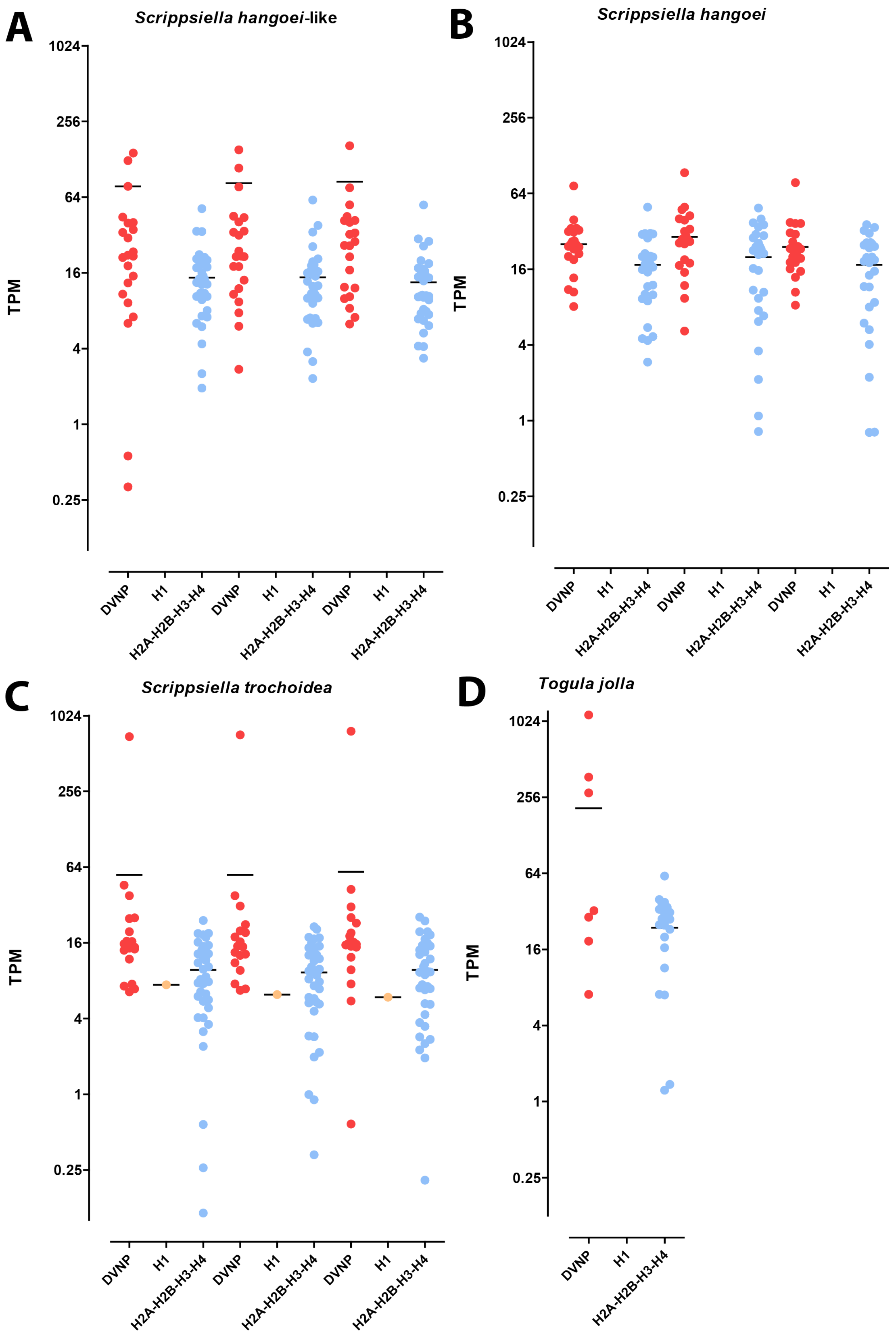


**Figure S6: Expression levels of DVNP, linker histone and histone genes in dinoflagellates**. (A) *Scrippsiella hangoei*-like; from left to right: SRR1296793, SRR1296794, SRR1296796; (B) *Scrippsiella hangoei*; from left to right: SRR1294400, SRR1296786, SRR1296972; (C) *Scrippsiella trochoidea*; for left to right: SRR1296759, SRR1296760, SRR1296761; (D) *Togula jolla*: SRR1296741.

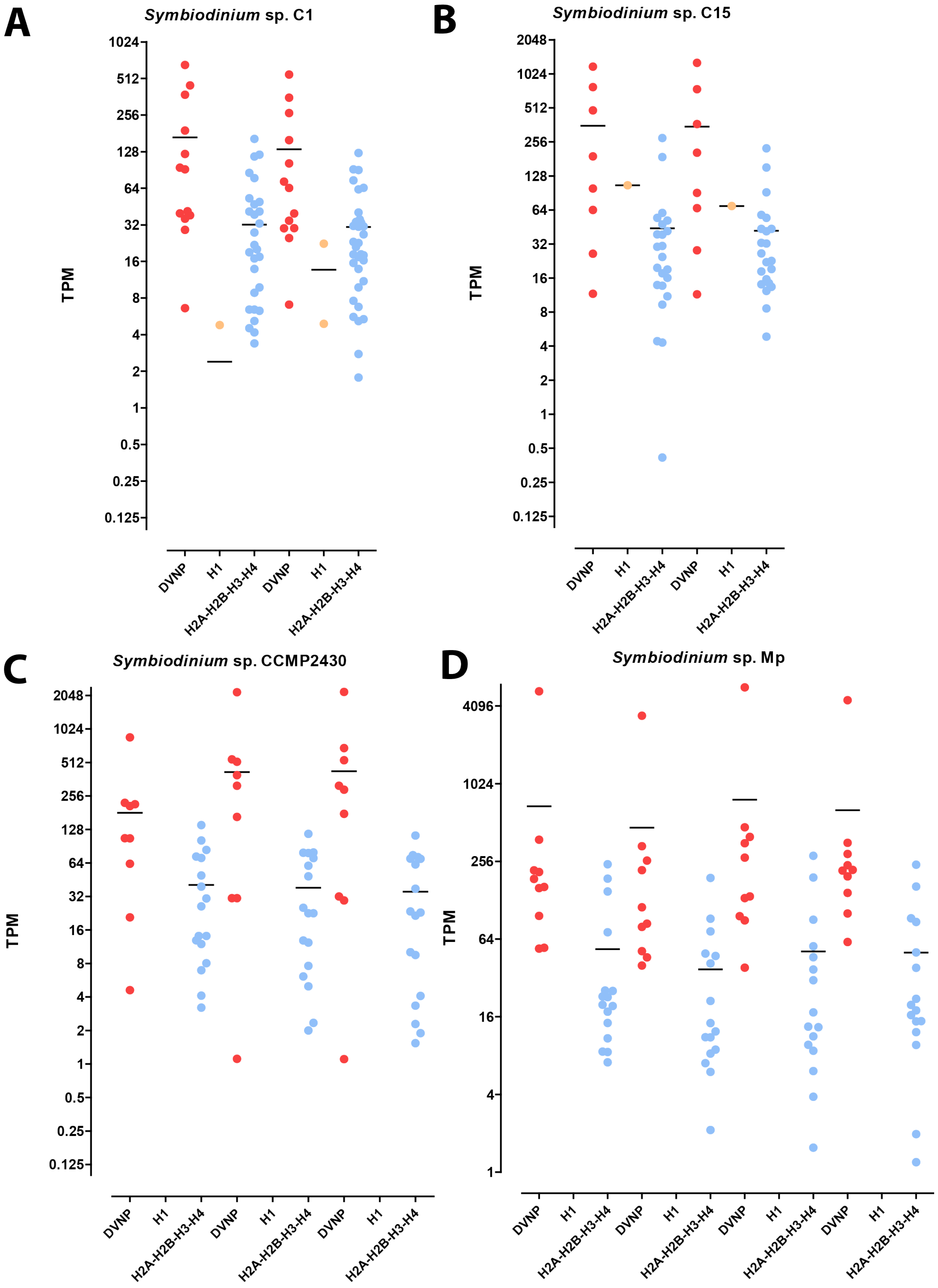


**Figure S7: Expression levels of DVNP, linker histone and histone genes in dinoflagellates**. (A) *Symbiodinium* sp. C1; from left to right: SRR1300430, SRR1300431; (B) *Symbiodinium* sp. C15; from left to right: SRR1300470, SRR1300471; (C) *Symbiodinium* sp. CCMP2430; for left to right: SRR1300264, SRR1300265, SRR1300266; (D) *Symbiodinium* sp. Mp; from left to right: SRR1300267, SRR1300343, SRR1300344, SRR1300345.

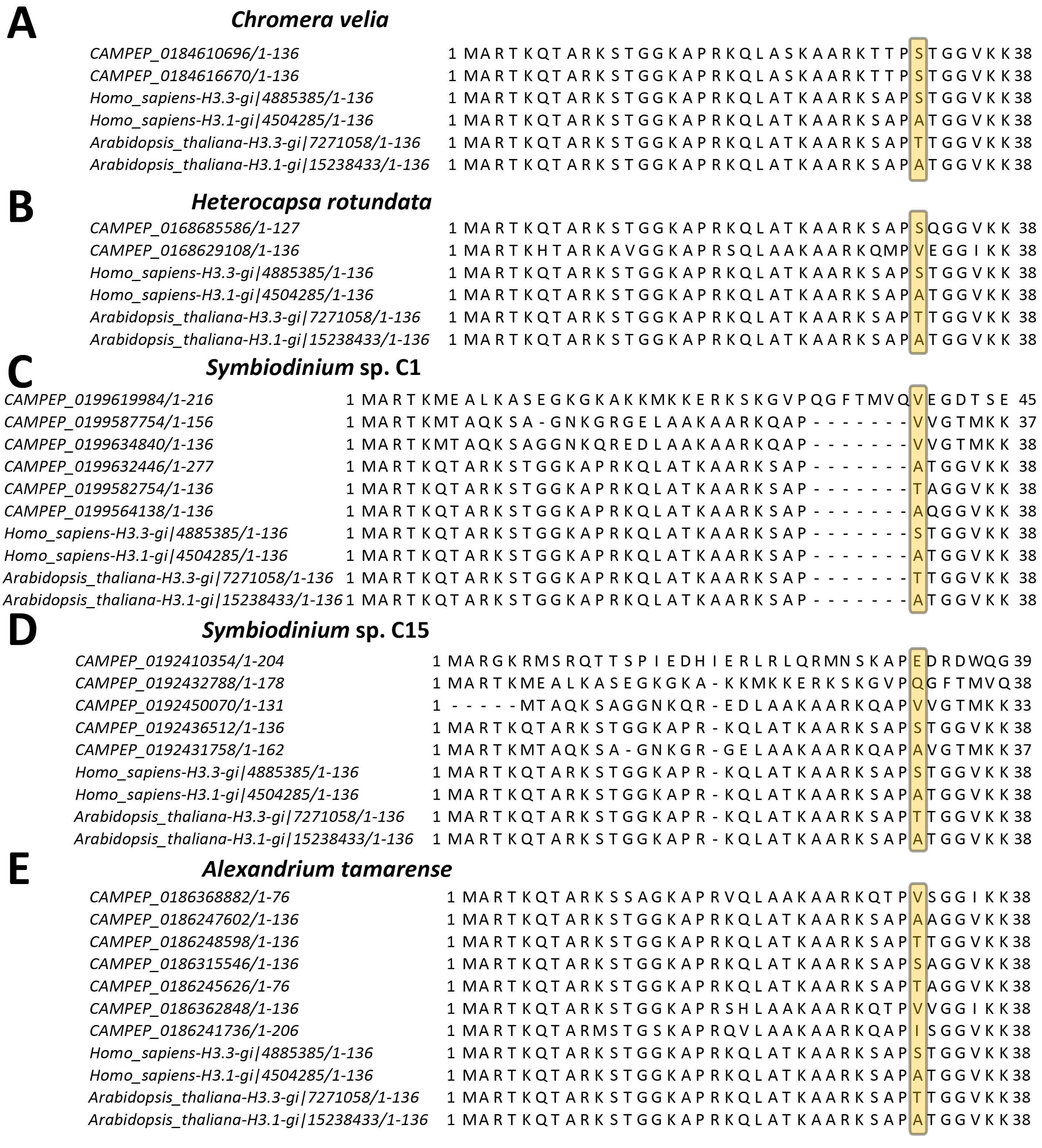


**Figure S8: Putative H3.3/H3.1 histone variants in dinoflagellates**. Histones H3.3 and H3.1 are distinguished by the sequence at position 31, which is S or T (in animals and fungi, respectively) in H3.3 and A in H3.1. Slightly different sequences are observed in other groups, for example in *Tetrahymena* the H3.3/H3.1 distinction is that the sequence is VS vs AT, respectively (Talbert et al. 2012). Putative H3.3/H3.1 pairs ar observed in several dinoflagellate transcriptomes: *Heterocapsa rotundata* (B), *Symbiodinium* sp. C1 (C), *Symbiodinium* sp. C15 (D), and *Alexandrium tamarense* (E). Only H3.3 variants are observed in the *Chromera velia* transcriptome, thus caution with respect to false negatives have to be exercised when interpreting the presence/absence of H3.3/H3.1 variants.

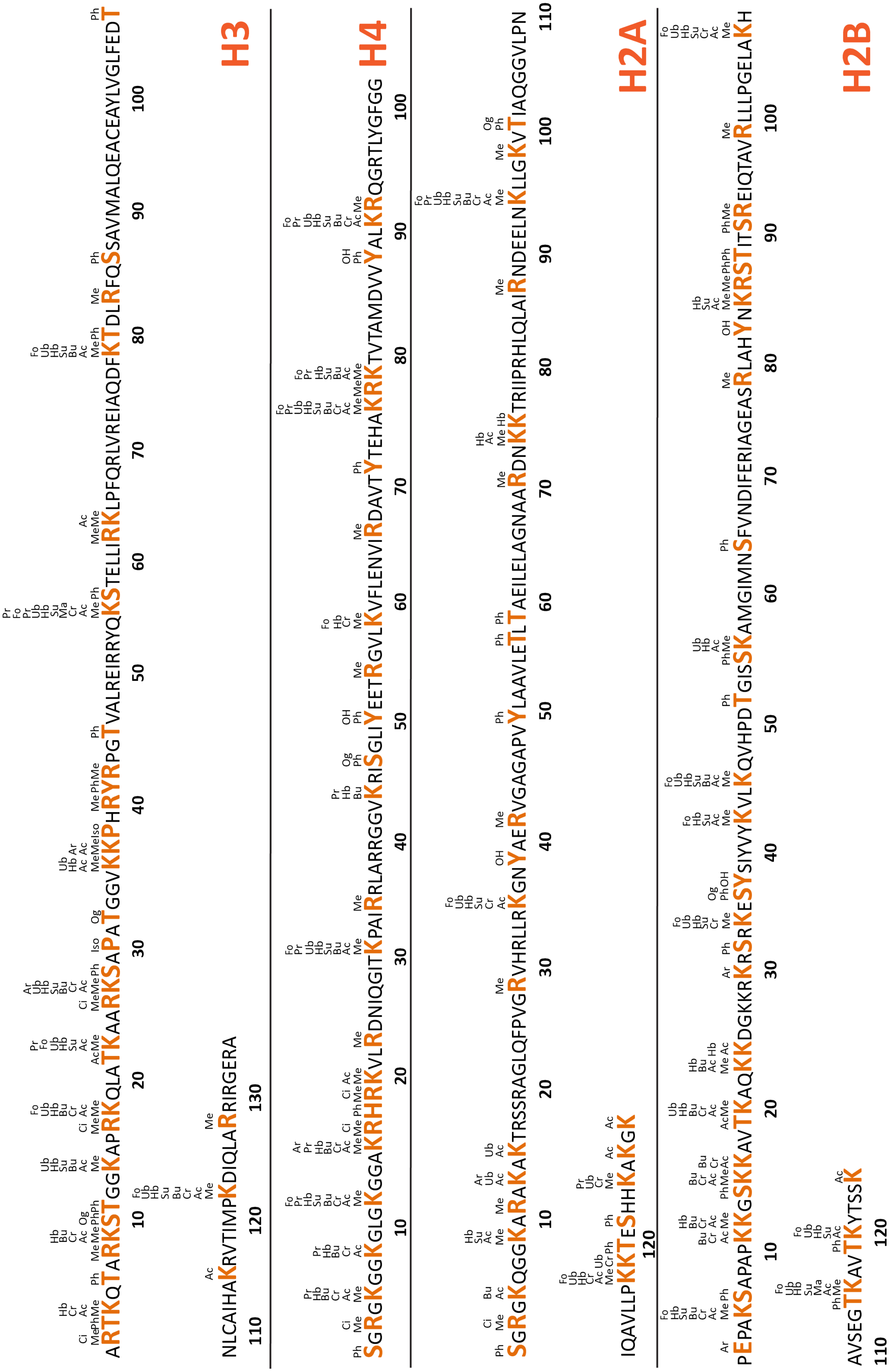
H3
H4
H2A
H2B

Figure S9 ***(preceding page)*:** **Known histone modifications in vertebrates**. *Me*: methylation (which can be mono-, di-, and trimethylation for lysines, and mono-, and symmetric and asymmetric dimethylation for arginines); *Ac*: acetylation; *Ub*: monoubiquitination; *Ph*: phosphorylation; *Cit*: citrullination; *Iso*: proline isomerization; *Pr*: propionylation; *Bu*: butyrylation; *Cr*: crotonylation; *Hb*; 2-Hydroxyisobutyrylation; *Ma*: malonylation; *Su*: succinylation; *Fo*: formylation; *OH*: hydroxylation; *Og*: O-GlcNAcylation; *Ar*: ADP ribosylation. The list of modification is mostly derived from (Huang et al. 2014).

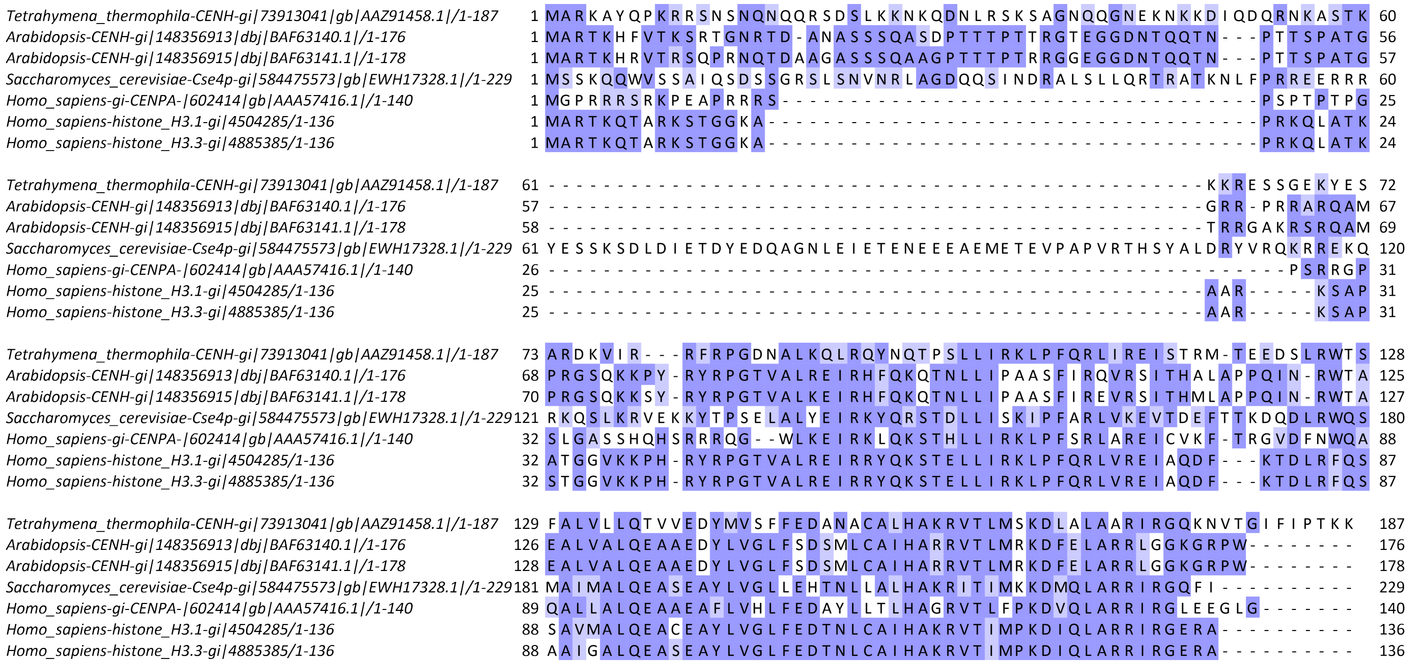


**Figure S10: Multiple sequences alignments of core histones H3 sequences and centromeric H3 variants in several eukaryotes.**. Sequences were aligned using MUSCLE (Edgar 2004) (version 3.8.31) and visualized using JalView (Waterhouse et al. 2009) (version 2.8.2).

```
CAMPEP_0200546290/1-167    1 MARTKQTARMSTGSKAPRQVLAAKTVRKQAPISGGVKKPHRYRPGTVA  48
CAMPEP_0200538292/1-136    1 MARTKQTARKSSAGKAPRVQLAAKAARKQSPVVGGIKKPHRYRAGTVA  48
Homo_sapiens/1-136         1 MARTKQTARKSTGGKAPRKQLATKAARKSAPATGGVKKPHRYRPGTVA  48
CAMPEP_0200540706/1-316    1 MARTKQTARKSTGGKAPRSQLAAKAARKQTPVAGGIKKPHRYRPGTVA  48

CAMPEP_0200546290/1-167   49 LREIRKYQKSTELLIRKLPFQRLVREIAQDFKTDLKFQSQAVLALQEA  96
CAMPEP_0200538292/1-136   49 LREIRKYQKSTDLLLRKLPFQRLVREIAQAFKSEVLFQSQAVLALQEA  96
Homo_sapiens/1-136        49 LREIRRYQKSTELLIRKLPFQRLVREIAQDFKTDLRFQSSAVMALQEA  96
CAMPEP_0200540706/1-316   49 LREIRKYQKSTDLLIRKLPFQRLVREIAQDFKTDLKFQSQAVLALQEA  96

CAMPEP_0200546290/1-167   97 AEAYLVGLFEDTNLCAIHAKRVTIMPKDVQLARRLRGERIEADGPLLL 144
CAMPEP_0200538292/1-136   97 AEAYLVGLFEDTNLCAIHGRRVTIMPKDMQLARRLRGERV-------- 136
Homo_sapiens/1-136        97 CEAYLVGLFEDTNLCAIHAKRVTIMPKDIQLARRIRGERA-------- 136
CAMPEP_0200540706/1-316   97 AEAYLVGLFEDTNLCAIHAKRVTIMPKDVQLARRLRGERIEAHDAFVF 144

CAMPEP_0200546290/1-167  145 PLPQASLACEETCNYVFLGGAVQ------------------------- 167
CAMPEP_0200538292/1-136      ------------------------------------------------
Homo_sapiens/1-136           ------------------------------------------------
CAMPEP_0200540706/1-316  145 LYKLVARAARRRVLPRQADDAWAAVNARSIASIPPIGAHAPAAGVQPV 192

CAMPEP_0200546290/1-167      ------------------------------------------------
CAMPEP_0200538292/1-136      ------------------------------------------------
Homo_sapiens/1-136           ------------------------------------------------
CAMPEP_0200540706/1-316  193 LPQPIPASCVWGGSPQMAEGYEEXSRPWQTASCARPPGGERAEHAVPS 240

CAMPEP_0200546290/1-167      ------------------------------------------------
CAMPEP_0200538292/1-136      ------------------------------------------------
Homo_sapiens/1-136           ------------------------------------------------
CAMPEP_0200540706/1-316  241 CARGAARRRRDGGEVAPVHALGPRGLDLLGSEAGSVGRVHGRLAFRDG 288

CAMPEP_0200546290/1-167      ----------------------------
CAMPEP_0200538292/1-136      ----------------------------
Homo_sapiens/1-136           ----------------------------
CAMPEP_0200540706/1-316  289 RALGAAVATRVMGRCGNSLPMVKSKKTX                     316
```

**Figure S11: Multiple sequences alignments of histones H3 sequences from *Alexandrium monilatum* and histone H3.1 from *Homo sapiens*.**. Sequences were aligned using MUSCLE (Edgar 2004) (version 3.8.31) and visualized using JalView (Waterhouse et al. 2009) (version 2.8.2).

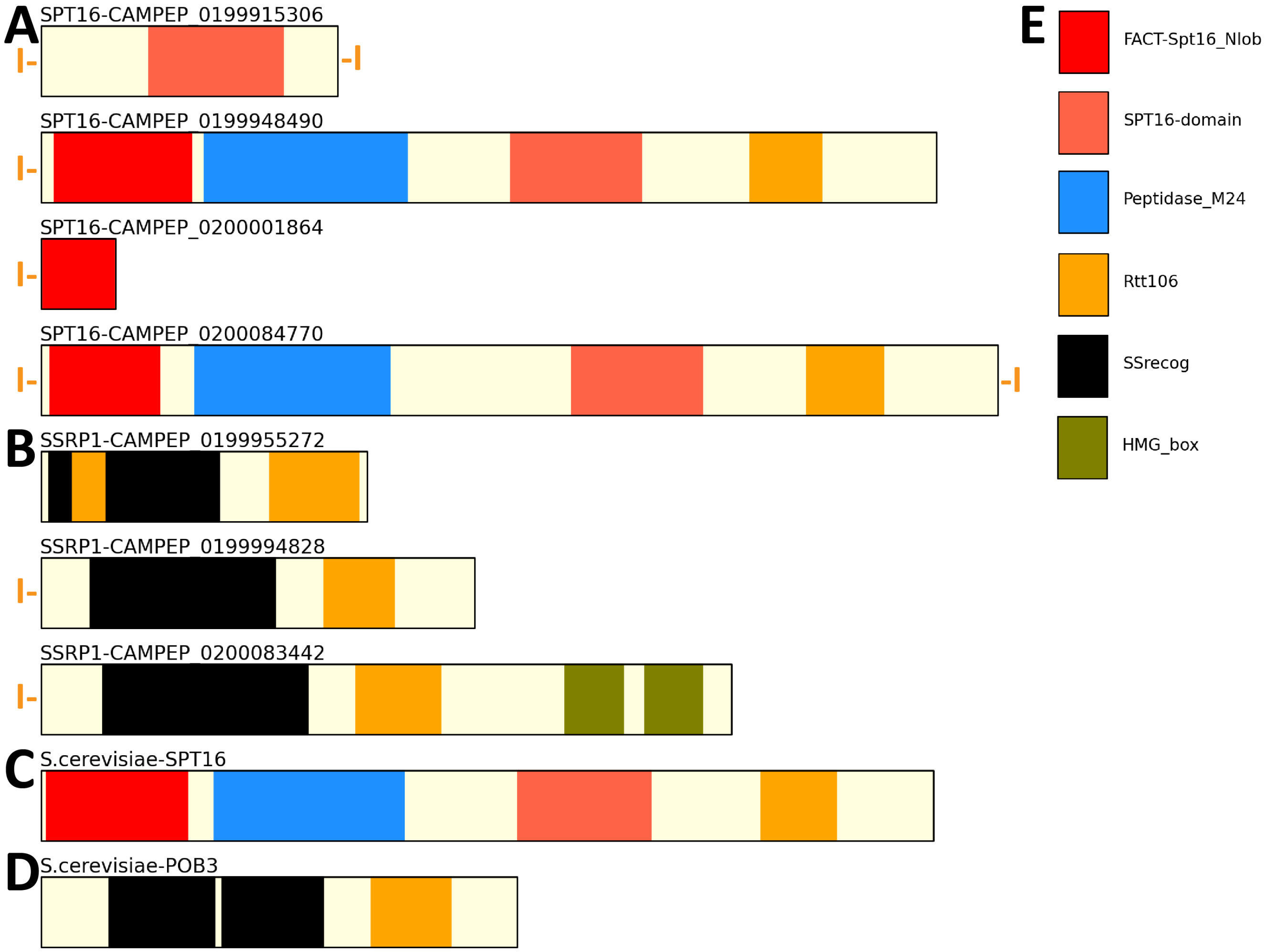


**Figure S12: FACT complex subunits and their domain organization in *Durinskia baltica*.** (A) *Durinskia baltica* SPT16 proteins; (B) *Durinskia baltica* SSRP1 proteins; (C) *Saccharomyces cerevisiae* SPT16; (D) *Saccharomyces cerevisiae* SPT16; (E) Domain color code. An orange "I" in front and/or after the protein indicates that the protein sequence is known to be not represented completely in the transcriptome assembly (note that its absence does not mean that the sequence is complete).

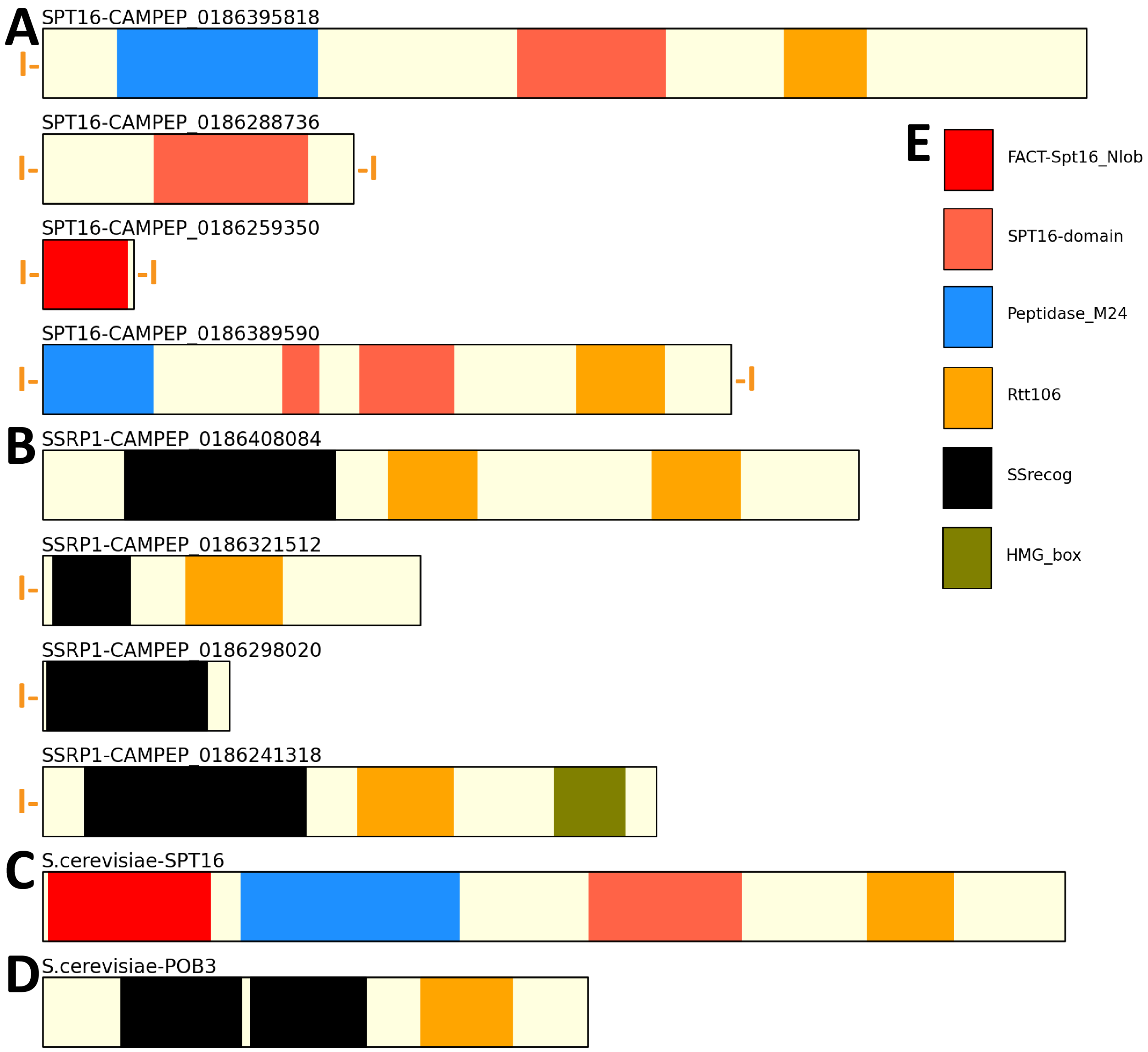


**Figure S13: FACT complex subunits and their domain organization in *Alexandrium tamarense*.** (A) *Alexandrium tamarense* SPT16 proteins; (B) *Alexandrium tamarense* SSRP1 proteins; (C) *Saccharomyces cerevisiae* SPT16; (D). *Saccharomyces cerevisiae* SPT16; (E) Domain color code. An orange "I" in front and/or after the protein indicates that the protein sequence is known to be not represented completely in the transcriptome assembly (note that its absence does not mean that the sequence is complete).

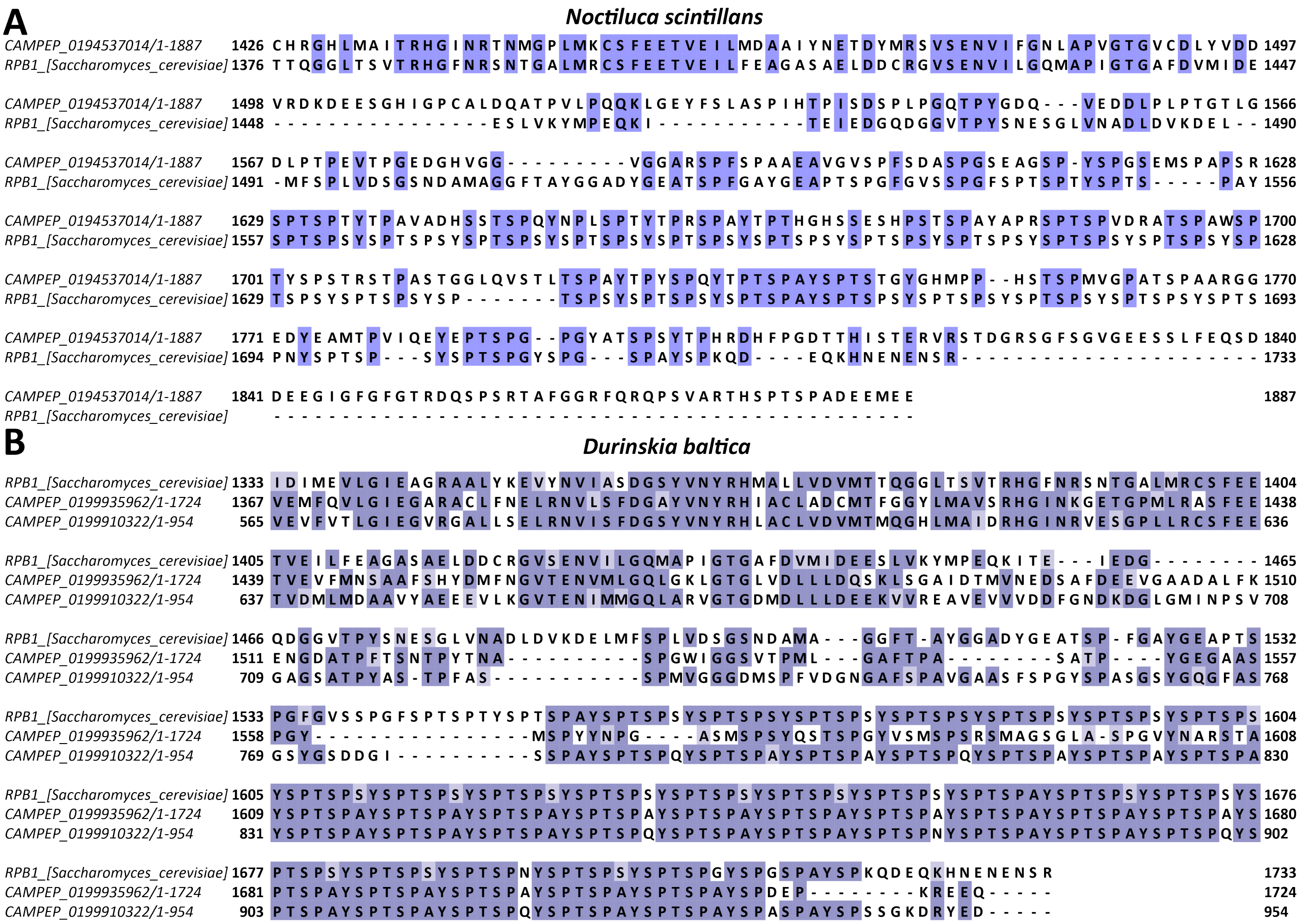


**Figure S14: RNA Polymerase II largest subunit CTD repeats in dinoflagellates**. Proteins were aligned using MUSCLE and the alignments visualized using JalView. The *Saccharomyces cerevisiae* Rpb1 protein was used as a reference. Only the C-terminal portion of the alignments is shown. (A) Candidate Rpb1 protein from *Noctiluca scintillans* with divergent C-terminal repeats (representative of the state in most dinoflagellates); (B). Candidate Rpb1 proteins from the dinotome *Durinskia baltica* showing higher level of conservation of the C-terminal repeats (almost certainly at least one of these sequences derives from the endosymbiont).
