# Supplementary material for: Diversity and Divergence of Dinoflagellate Histone Proteins
Source: G3 (Bethesda). 2015 Dec 8;6(2):397–422. doi: 10.1534/g3.115.023275 (PMC4751559; doi:10.1534/g3.115.023275)
Supplement: Supporting Information [file supp_g3.115.023275_FigureS9.pdf]

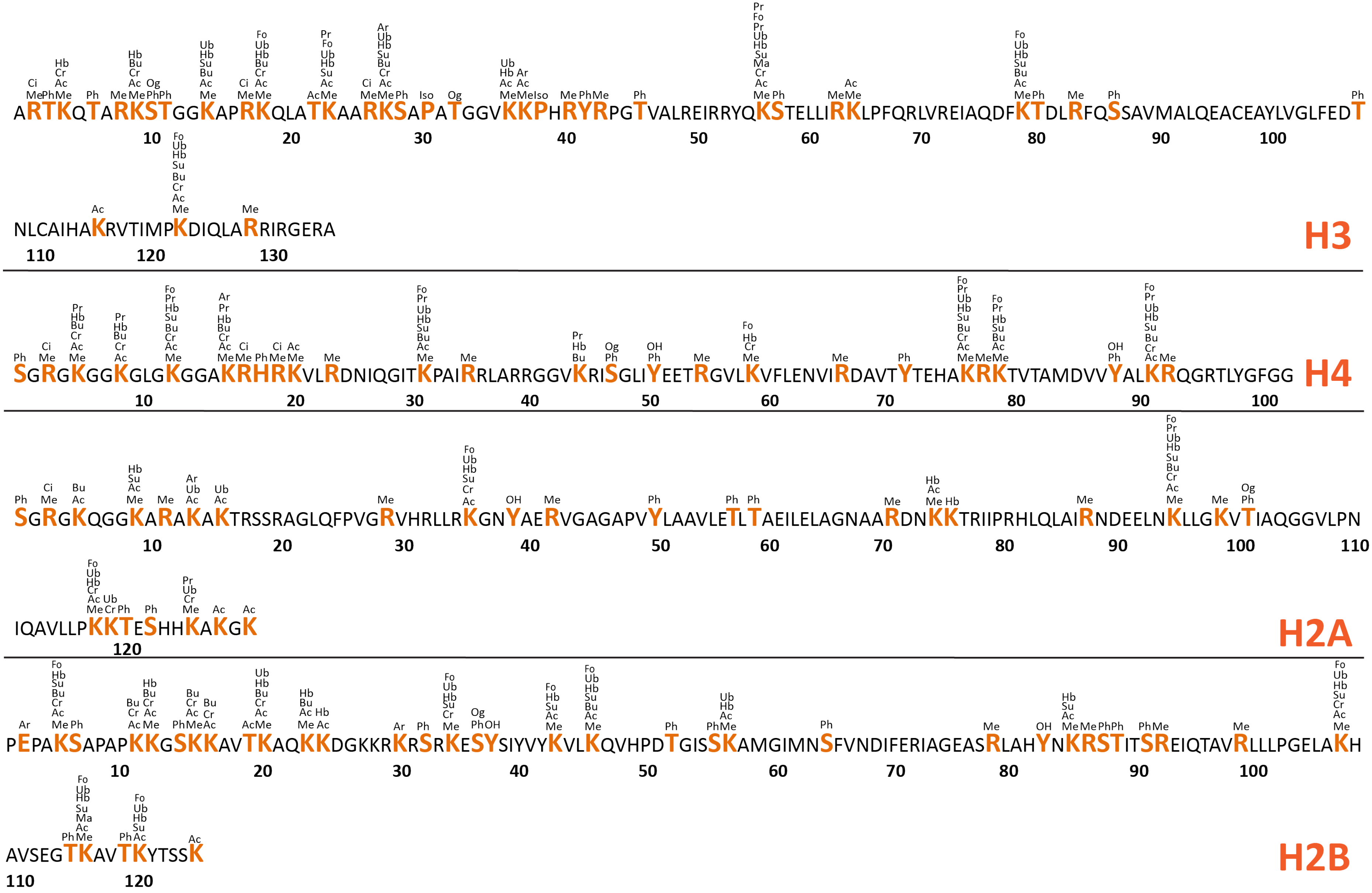

H3
ARTKQTARKSTGGKAPRKQLATKAARKSAPATGGVKKPHRYRPGTVALREIRRYQKSTELLIRKLPFQRLVREIAQDFKTDLRFQSSAVMALQEACEAYLVGLFEDT
NLCAIHAKRVTIMPKDIQLARRIRGERA
H4
SGRGKGGKGLGKGGAKRHRKVLRDNIQGITKPAIRRLARRGGVKRISGLIYEETRGVLKVFLENVIRDAVTYTEHAKRKTVTAMDVVYALKRQGRTLYGFGG
H2A
SGRGKQGGKARAKAKTRSSRAGLQFPVGRVHRLLRKGNYAERVGAGAPVYLAAVLETLTAEILELAGNAARDNKKTRIIPRHLQLAIRNDEELNKLLGKVTIAQGGVLPN
IQAVLLPKKTESHHKAKGK
H2B
PEPAKSAPAPKKGSKKAVTKAQKKDGKKRKRSRKESYSIYVYKVLKQVHPDTGISSKAMGIMNSFVNDIFERIAGEASRLAHYNKRSTITSREIQTAVRLLLPGELAKH
AVSEGTKAVTKYTSSK

Figure S9 *(preceding page)*: Known histone modifications in vertebrates. *Me*: methylation (which can be mono-, di-, and trimethylation for lysines, and mono-, and symmetric and asymmetric dimethylation for arginines); *Ac*: acetylation; *Ub*: monoubiquitination; *Ph*: phosphorylation; *Cit*: citrullination; *Iso*: proline isomerization; *Pr*: propionylation; *Bu*: butyrylation; *Cr*: crotonylation; *Hb*; 2-Hydroxyisobutyrylation; *Ma*: malonylation; *Su*: succinylation; *Fo*: formylation; *OH*: hydroxylation; *Og*: O-GlcNAcylation; *Ar*: ADP ribosylation. The list of modification is mostly derived from (Huang et al. 2014).
