# Supplementary material for: Diversity and Divergence of Dinoflagellate Histone Proteins
Source: G3 (Bethesda). 2015 Dec 8;6(2):397–422. doi: 10.1534/g3.115.023275 (PMC4751559; doi:10.1534/g3.115.023275)
Supplement: Supporting Information [file supp_g3.115.023275_FigureS10.pdf]

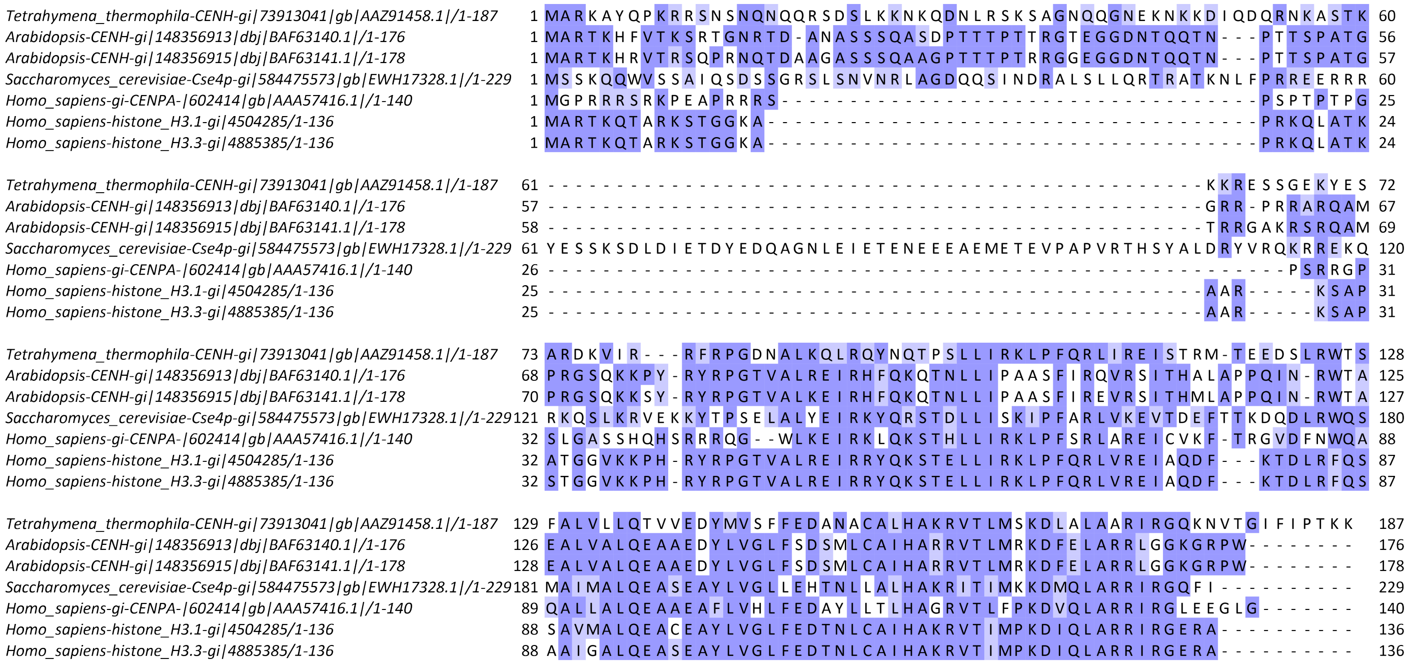


**Figure S10: Multiple sequences alignments of core histones H3 sequences and centromeric H3 variants in several eukaryotes..** Sequences were aligned using MUSCLE (Edgar 2004) (version 3.8.31) and visualized using JalView (Waterhouse et al. 2009) (version 2.8.2).
