# Supplementary material for: Diversity and Divergence of Dinoflagellate Histone Proteins
Source: G3 (Bethesda). 2015 Dec 8;6(2):397–422. doi: 10.1534/g3.115.023275 (PMC4751559; doi:10.1534/g3.115.023275)
Supplement: Supporting Information [file supp_g3.115.023275_FigureS12.pdf]

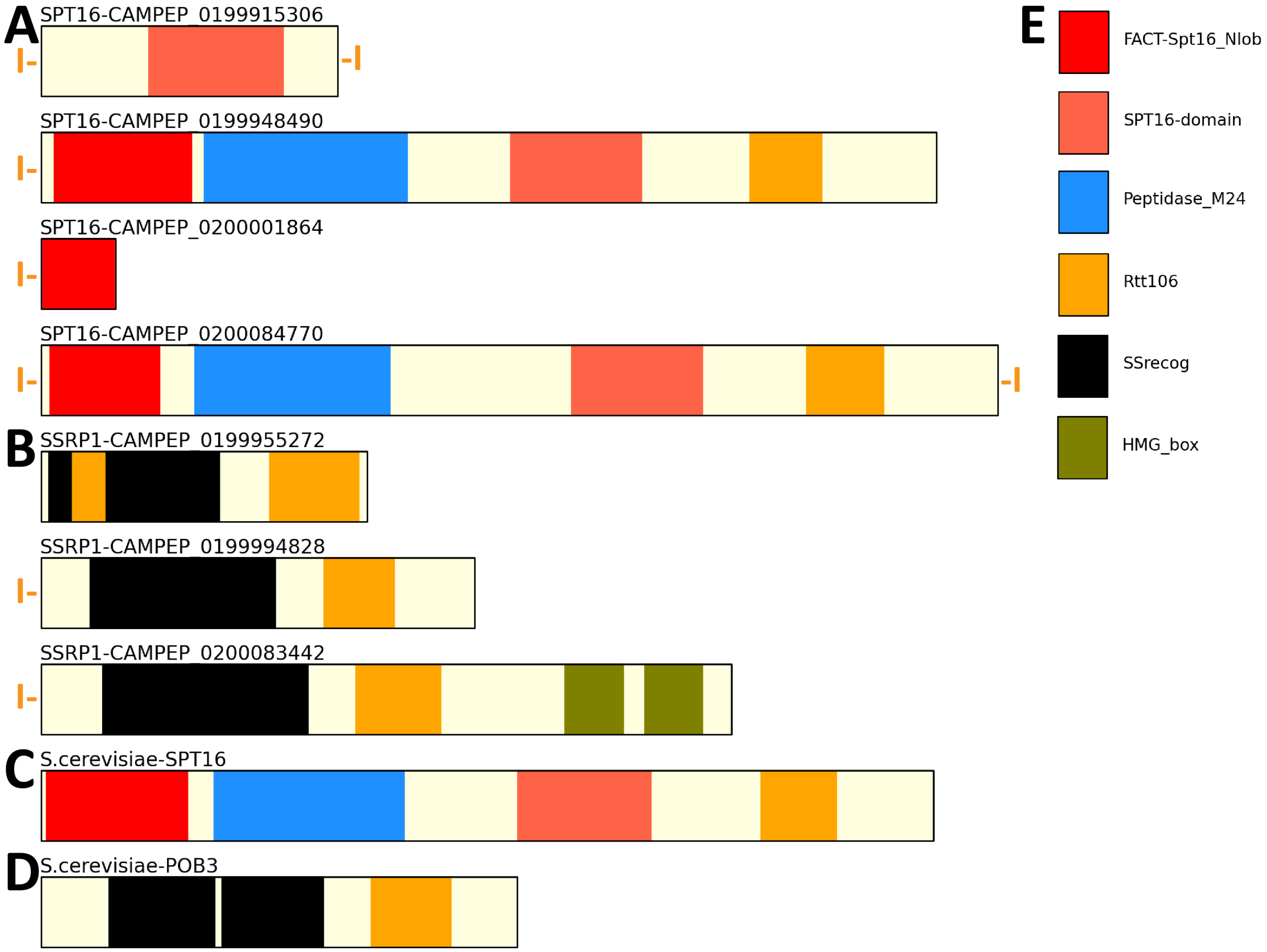

Figure S12: FACT complex subunits and their domain organization in *Durinskia baltica*. (A) *Durinskia baltica* SPT16 proteins; (B) *Durinskia baltica* SSRP1 proteins; (C) *Saccharomyces cerevisiae* SPT16; (D) *Saccharomyces cerevisiae* SPT16; (E) Domain color code. An orange "I" in front and/or after the protein indicates that the protein sequence is known to be not represented completely in the transcriptome assembly (note that its absence does not mean that the sequence is complete).
