# Supplementary material for: Diversity and Divergence of Dinoflagellate Histone Proteins
Source: G3 (Bethesda). 2015 Dec 8;6(2):397–422. doi: 10.1534/g3.115.023275 (PMC4751559; doi:10.1534/g3.115.023275)
Supplement: Supporting Information [file supp_g3.115.023275_FigureS14.pdf]

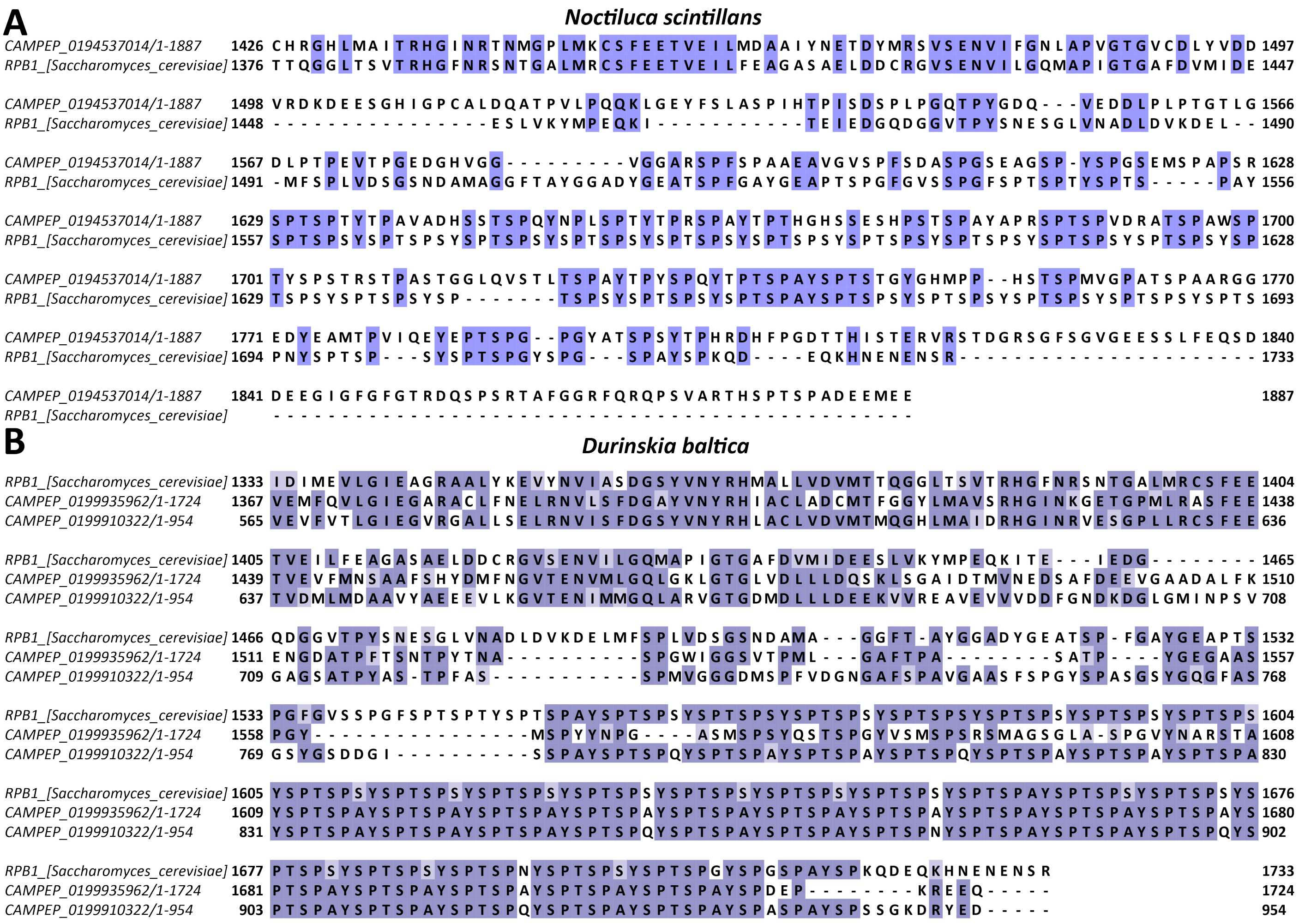

Figure S14: RNA Polymerase II largest subunit CTD repeats in dinoflagellates. Proteins were aligned using MUSCLE and the alignments visualized using JalView. The *Saccharomyces cerevisiae* Rpb1 protein was used as a reference. Only the C-terminal portion of the alignments is shown. (A) Candidate Rpb1 protein from *Noctiluca scintillans* with divergent C-terminal repeats (representative of the state in most dinoflagellates); (B). Candidate Rpb1 proteins from the dinotome *Durinskia baltica* showing higher level of conservation of the C-terminal repeats (almost certainly at least one of these sequences derives from the endosymbiont).
