# Supplementary material for: Diversity and Divergence of Dinoflagellate Histone Proteins
Source: G3 (Bethesda). 2015 Dec 8;6(2):397–422. doi: 10.1534/g3.115.023275 (PMC4751559; doi:10.1534/g3.115.023275)
Supplement: Supporting Information [file supp_g3.115.023275_TableS1.pdf]

Table S1: Summary of MMETSP samples used in this study. The table is based in information provided by the MMETSP project page.

Sample name	Group	Family	Species	Strain	Clonal	Axenic
MMETSP0093	Dinoflagellata	Goniodomataceae	*Alexandrium monilatum*	CCMP3105	1	No
MMETSP0095	Dinoflagellata	Goniodomataceae	*Alexandrium monilatum*	CCMP3105	1	No
MMETSP0096	Dinoflagellata	Goniodomataceae	*Alexandrium monilatum*	CCMP3105	1	No
MMETSP0097	Dinoflagellata	Goniodomataceae	*Alexandrium monilatum*	CCMP3105	1	No
MMETSP0378	Dinoflagellata	Goniodomataceae	*Alexandrium tamarense*	CCMP1771	1	1
MMETSP0380	Dinoflagellata	Goniodomataceae	*Alexandrium tamarense*	CCMP1771	1	1
MMETSP0382	Dinoflagellata	Goniodomataceae	*Alexandrium tamarense*	CCMP1771	1	1
MMETSP0384	Dinoflagellata	Goniodomataceae	*Alexandrium tamarense*	CCMP1771	1	1
MMETSP0795	Dinoflagellata	Goniodomaceae	*Amoebophrya* sp.	Ameob2	1	No
MMETSP0258	Dinoflagellata	Gymnodiniaceae	*Amphidinium carterae*	CCMP1314	Unknown	No
MMETSP0259	Dinoflagellata	Gymnodiniaceae	*Amphidinium carterae*	CCMP1314	Unknown	No
MMETSP0398C	Dinoflagellata	Gymnodiniaceae	*Amphidinium carterae*	CCMP1314	1	No
MMETSP0399	Dinoflagellata	Gymnodiniaceae	*Amphidinium carterae*	CCMP1314	1	
MMETSP1036	Dinoflagellata	Unknown	*Azadinium spinosum*	3D9	1	1
MMETSP1037	Dinoflagellata	Unknown	*Azadinium spinosum*	3D9	1	1
MMETSP1038	Dinoflagellata	Unknown	*Azadinium spinosum*	3D9	1	1
MMETSP1462	Dinoflagellata	Peridiniaceae	*Brandtodinium nutriculum*	RCC3387	1	No
MMETSP1074	Dinoflagellata	Ceratiaceae	*Ceratium fusus*	PA161109	1	No
MMETSP1075	Dinoflagellata	Ceratiaceae	*Ceratium fusus*	PA161109	1	No
MMETSP0323	Dinoflagellata	Crypthecodiniacea	*Crypthecodinium cohnii*	Seligo	1	1
MMETSP0324	Dinoflagellata	Crypthecodiniacea	*Crypthecodinium cohnii*	Seligo	1	1
MMETSP0325	Dinoflagellata	Crypthecodiniacea	*Crypthecodinium cohnii*	Seligo	1	1
MMETSP0326	Dinoflagellata	Crypthecodiniacea	*Crypthecodinium cohnii*	Seligo	1	1
MMETSP0797	Dinoflagellata	Dinophysiaceae	*Dinophysis acuminata*	DAEP01	Unknown	No
MMETSP0116	Dinoflagellata	Peridiniaceae	*Durinskia baltica*	CSIRO CS-38	No	No
MMETSP0117	Dinoflagellata	Peridiniaceae	*Durinskia baltica*	CSIRO CS-38	No	No
MMETSP0766	Dinoflagellata	Goniodomaceae	*Gambierdiscus australes*	CAWD 149	1	No
MMETSP0118	Dinoflagellata	Peridiniaceae	*Glenodinium foliaceum*	CCAP 1116/3	No	No
MMETSP0119	Dinoflagellata	Peridiniaceae	*Glenodinium foliaceum*	CCAP 1116/3	No	No
MMETSP1439	Dinoflagellata	Gonyaulacaceae	*Gonyaulax spinifera*	CCMP409	Unknown	No
MMETSP0784	Dinoflagellata	Gymnodiniaceae	*Gymnodinium catenatum*	GC744	1	No
MMETSP1148	Dinoflagellata	Gymnodiniaceae	*Gyrodinium dominans*	SPMC 103	No	No
MMETSP0503	Dinoflagellata	Heterocapsaceae	*Heterocapsa rotundata*	SCCAP K-0483	No	No
MMETSP0448	Dinoflagellata	Heterocapsaceae	*Heterocapsa triquestra*	CCMP 448	1	No
MMETSP0027	Dinoflagellata	Gymnodiniaceae	*Karenia brevis*	CCMP2229	1	No
MMETSP0029	Dinoflagellata	Gymnodiniaceae	*Karenia brevis*	CCMP2229	1	No
MMETSP0030	Dinoflagellata	Gymnodiniaceae	*Karenia brevis*	CCMP2229	1	No
MMETSP0031	Dinoflagellata	Gymnodiniaceae	*Karenia brevis*	CCMP2229	1	No
MMETSP0201	Dinoflagellata	Gymnodiniaceae	*Karenia brevis*	Wilson	Unknown	No
MMETSP0202	Dinoflagellata	Gymnodiniaceae	*Karenia brevis*	Wilson	Unknown	No
MMETSP0527	Dinoflagellata	Gymnodiniaceae	*Karenia brevis*	SP3	1	No
MMETSP0528	Dinoflagellata	Gymnodiniaceae	*Karenia brevis*	SP3	1	No
MMETSP0573	Dinoflagellata	Gymnodiniaceae	*Karenia brevis*	SP1	1	No
MMETSP0574	Dinoflagellata	Gymnodiniaceae	*Karenia brevis*	SP1	1	No
MMETSP0648	Dinoflagellata	Gymnodiniaceae	*Karenia brevis*	Wilson	1	No
MMETSP0649	Dinoflagellata	Gymnodiniaceae	*Karenia brevis*	Wilson	1	No
MMETSP1015	Dinoflagellata	Gymnodiniaceae	*Karlodinium micrum*	CCMP2283	Unknown	No
MMETSP1016	Dinoflagellata	Gymnodiniaceae	*Karlodinium micrum*	CCMP2283	Unknown	No
MMETSP1017	Dinoflagellata	Gymnodiniaceae	*Karlodinium micrum*	CCMP2283	Unknown	No
MMETSP0120	Dinoflagellata	Peridiniaceae	*Kryptoperidinium foliaceum*	CCMP 1326	No	No
MMETSP0121	Dinoflagellata	Peridiniaceae	*Kryptoperidinium foliaceum*	CCMP 1326	No	No

Continued on next page

Table S1 – *Continued from previous page*

Sample name	Group	Family	Species	Strain	Clonal	Axenic
MMETSP1032	Dinoflagellata	Gonyaulacaceae	*Lingulodinium polyedra*	CCMP 1738	1	No
MMETSP1033	Dinoflagellata	Gonyaulacaceae	*Lingulodinium polyedra*	CCMP 1738	1	No
MMETSP1034	Dinoflagellata	Gonyaulacaceae	*Lingulodinium polyedra*	CCMP 1738	1	No
MMETSP1035	Dinoflagellata	Gonyaulacaceae	*Lingulodinium polyedra*	CCMP 1738	1	No
MMETSP0253	Dinoflagellata	Noctilucaceae	*Noctiluca scintillans*		No	No
MMETSP0468	Dinoflagellata	Oxyrrhinaceae	*Oxyrrhis marina*		No	No
MMETSP0469	Dinoflagellata	Oxyrrhinaceae	*Oxyrrhis marina*		No	No
MMETSP0470	Dinoflagellata	Oxyrrhinaceae	*Oxyrrhis marina*		No	No
MMETSP0471	Dinoflagellata	Oxyrrhinaceae	*Oxyrrhis marina*		No	No
MMETSP1424	Dinoflagellata	Oxyrrhinaceae	*Oxyrrhis marina*	LB1974	No	No
MMETSP1425	Dinoflagellata	Oxyrrhinaceae	*Oxyrrhis marina*	LB1974	No	No
MMETSP1426	Dinoflagellata	Oxyrrhinaceae	*Oxyrrhis marina*	LB1974	No	No
MMETSP1338	Dinoflagellata	Suessiaceae	*Pelagodinium beii*	RCC1491		
MMETSP0370	Dinoflagellata	Peridiniaceae	*Peridinium aciculiferum*	PAER-2	1	No
MMETSP0371	Dinoflagellata	Peridiniaceae	*Peridinium aciculiferum*	PAER-2	1	No
MMETSP1440	Dinoflagellata	Suessiaceae	*Polarella glacialis*	CCMP2088	1	No
MMETSP0227	Dinoflagellata	Suessiaceae	*Polarella glacialis*	CCMP 1383	Unknown	No
MMETSP0053	Dinoflagellata	Prorocentraceae	*Prorocentrum minimum*	CCMP1329		1
MMETSP0055	Dinoflagellata	Prorocentraceae	*Prorocentrum minimum*	CCMP1329		1
MMETSP0056	Dinoflagellata	Prorocentraceae	*Prorocentrum minimum*	CCMP1329		1
MMETSP0057	Dinoflagellata	Prorocentraceae	*Prorocentrum minimum*	CCMP1329		1
MMETSP0267	Dinoflagellata	Prorocentraceae	*Prorocentrum minimum*	CCMP2233	Unknown	No
MMETSP0268	Dinoflagellata	Prorocentraceae	*Prorocentrum minimum*	CCMP2233	Unknown	No
MMETSP0269	Dinoflagellata	Prorocentraceae	*Prorocentrum minimum*	CCMP2233	Unknown	No
MMETSP0228	Dinoflagellata	Gonyaulacaceae	*Protoceratium reticulatum*	CCCM535 (=CCMP1889)	Unknown	No
MMETSP0796	Dinoflagellata	Gonyaulacaceae	*Pyrodinium bahamense*	pbaha01	1	No
MMETSP0359	Dinoflagellata	Peridiniaceae	*Scrippsiella hangoei*	SHTV-5	1	No
MMETSP0360	Dinoflagellata	Peridiniaceae	*Scrippsiella hangoei*	SHTV-5	1	No
MMETSP0361	Dinoflagellata	Peridiniaceae	*Scrippsiella hangoei*	SHTV-5	1	No
MMETSP0367	Dinoflagellata	Peridiniaceae	*Scrippsiella hangoei*-like	SHHI-4	1	No
MMETSP0368	Dinoflagellata	Peridiniaceae	*Scrippsiella hangoei*-like	SHHI-4	1	No
MMETSP0369	Dinoflagellata	Peridiniaceae	*Scrippsiella hangoei*-like	SHHI-4	1	No
MMETSP0270	Dinoflagellata	Peridiniaceae	*Scrippsiella trochoidea*	CCMP3099	No	No
MMETSP0271	Dinoflagellata	Peridiniaceae	*Scrippsiella trochoidea*	CCMP3099	No	No
MMETSP0272	Dinoflagellata	Peridiniaceae	*Scrippsiella trochoidea*	CCMP3099	No	No
MMETSP1115	Dinoflagellata	Symbiodiniaceae	*Symbiodinium* sp.	CCMP2430	No	No
MMETSP1116	Dinoflagellata	Symbiodiniaceae	*Symbiodinium* sp.	CCMP2430	No	No
MMETSP1117	Dinoflagellata	Symbiodiniaceae	*Symbiodinium* sp.	CCMP2430	No	No
MMETSP1122	Dinoflagellata	Symbiodiniaceae	*Symbiodinium* sp.	Mp	No	No
MMETSP1123	Dinoflagellata	Symbiodiniaceae	*Symbiodinium* sp.	Mp	No	No
MMETSP1124	Dinoflagellata	Symbiodiniaceae	*Symbiodinium* sp.	Mp	No	No
MMETSP1125	Dinoflagellata	Symbiodiniaceae	*Symbiodinium* sp.	Mp	No	No
MMETSP1367	Dinoflagellata	Symbiodiniaceae	*Symbiodinium* sp.	C1	Unknown	No
MMETSP1369	Dinoflagellata	Symbiodiniaceae	*Symbiodinium* sp.	C1	Unknown	No
MMETSP1370	Dinoflagellata	Symbiodiniaceae	*Symbiodinium* sp.	C15	Unknown	No
MMETSP1371	Dinoflagellata	Symbiodiniaceae	*Symbiodinium* sp.	C15	Unknown	No
MMETSP0224	Dinoflagellata	Gymnodiniaceae	*Togula jolla*	CCCM 725	Unknown	No
MMETSP0924C	Perkinsida	Perkinsidae	*Perkinsus chesapeaki*	ATCC PRA-65	1	No
MMETSP0925	Perkinsida	Perkinsidae	*Perkinsus chesapeaki*	ATCC PRA-65	1	
MMETSP0922	Perkinsida	Perkinsidae	*Perkinsus marinus*	ATCC 50439	1	No
MMETSP0923	Perkinsida	Perkinsidae	*Perkinsus marinus*	ATCC 50439	1	
MMETSP0290	Chromerida	Unknown	*Chromera velia*	CCMP2878	1	1
