# Supplementary material for: Diversity and Divergence of Dinoflagellate Histone Proteins
Source: G3 (Bethesda). 2015 Dec 8;6(2):397–422. doi: 10.1534/g3.115.023275 (PMC4751559; doi:10.1534/g3.115.023275)
Supplement: Supporting Information [file supp_g3.115.023275_TableS2.pdf]

Table S2: Putative H2A.X histone variants in dinoflagellates. The H2A.X variants of histone H2A are characterized by the presence of a SQ(E/D)Φ phosphorylation motif at the C-terminus of the protein (Talbert et al. 2012). Note that the motif is usually SQDY in heterokonts (Talbert et al. 2012), which include diatoms and thus the endosymbiont of dinotoms, thus one of proteins listed below in *Durinskia baltica* is most likely to be of endosymbiont origin.

Species	Protein	Length	C-terminal sequence
Perkinsus marinus	EER08766.1	137	**SQEM**
Perkinsus marinus	EER09215.1	135	**SQEM**
Perkinsus marinus	EER15538.1	162	**SQEM**
Perkinsus marinus	EER15802.1	136	**SQEI**
Perkinsus marinus	EEQ99722.1	155	**SQEM**
Perkinsus marinus	EER04007.1	164	**SQEM**
Perkinsus marinus	EER04402.1	164	**SQEM**
Perkinsus marinus	EEQ98671.1	138	**SQEM**
Perkinsus marinus	EEQ97488.1	92	**SQEM**
Symbiodinium sp. C15	CAMPEP_0192465542	177	**SQEY**
Symbiodinium sp. C1	CAMPEP_0199619000	181	**SQEY**
Symbiodinium sp. C1	CAMPEP_0199597416	160	**SQEY**
Scrippsiella trochoidea	CAMPEP_0192083196	204	**SQEY**
Polarella glacialis	CAMPEP_0115091146	166	**SQEY**
Pelagodinium beii	CAMPEP_0197627280	157	**SQEY**
Oxyrrhis marina LB1974	CAMPEP_0190412876	136	**SQQY**
Oxyrrhis marina	CAMPEP_0190349664	136	**SQQY**
Noctiluca scintillans	CAMPEP_0194480802	179	**SQEF**
Kryptoperidinium foliaceum	CAMPEP_0189651904	130	**SQEF**
Karlodinium micrum	CAMPEP_0200762398	199	**SQEF**
Glenodinium foliaceum	CAMPEP_0188370172	132	**SQEF**
Durinskia baltica	CAMPEP_0200040914	137	**SQDF**
Durinskia baltica	CAMPEP_0200047580	153	**SQDY**
Crypthecodinium cohnii	CAMPEP_0193858338	196	**SQEF**
Crypthecodinium cohnii	CAMPEP_0193883494	196	**SQEF**
Alexandrium tamarense	CAMPEP_0186381488	131	**SQSY**
Alexandrium tamarense	CAMPEP_0186337128	141	**SQEY**
Alexandrium monilatum	CAMPEP_0200550256	187	**SQEF**
Symbiodinium minutum	symbB.v1.2.004801.t1	177	**SQEY**
