# Supplementary material for: Diversity and Divergence of Dinoflagellate Histone Proteins
Source: G3 (Bethesda). 2015 Dec 8;6(2):397–422. doi: 10.1534/g3.115.023275 (PMC4751559; doi:10.1534/g3.115.023275)
Supplement: Supporting Information [file supp_g3.115.023275_FigureS1.pdf]

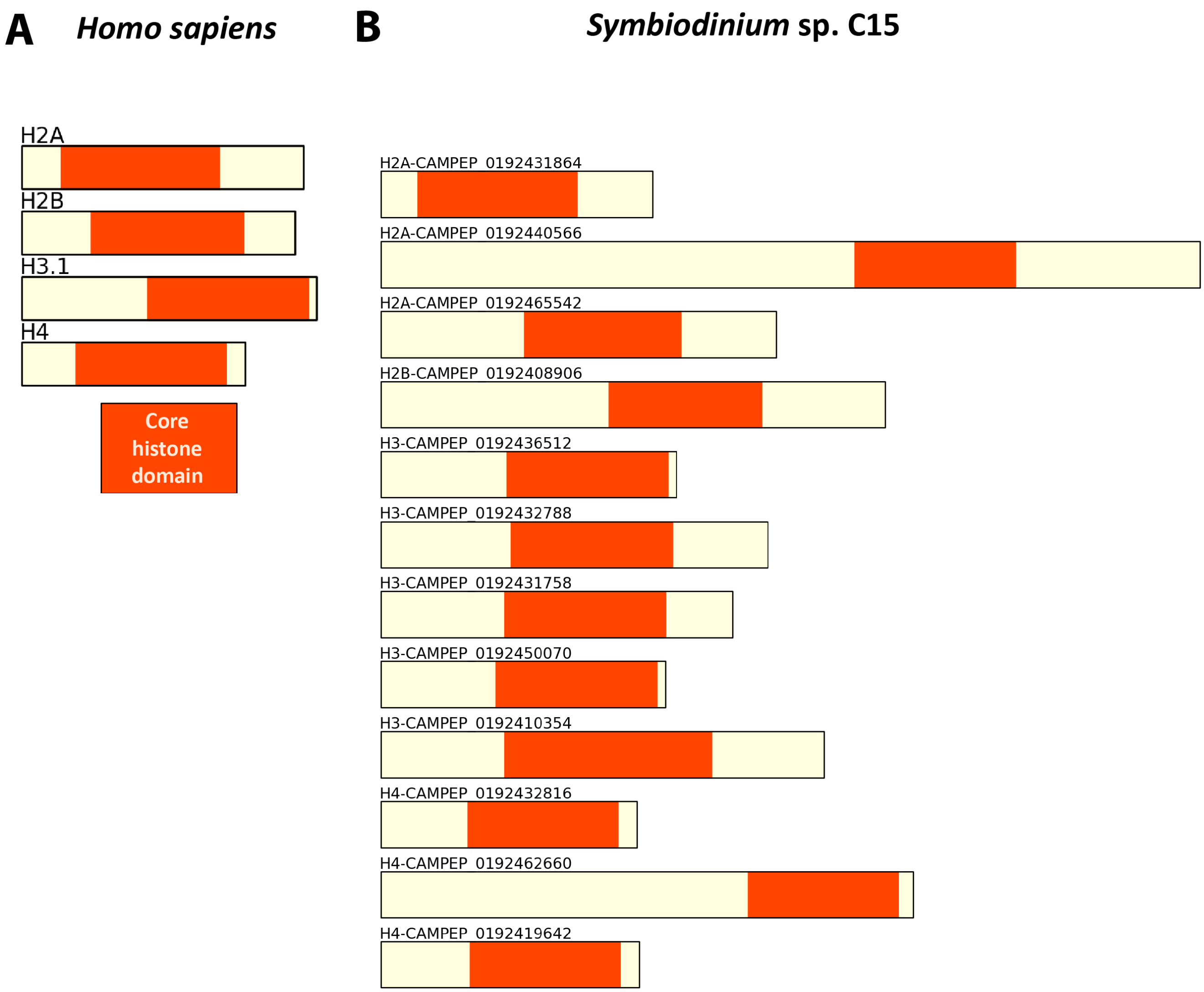

Figure S1: Protein domains in dinoflagellate histones. (A) *Homo sapiens* histones, shown for reference; (B) *Symbiodinium* sp. C15 histones.
