# Supplementary material for: Diversity and Divergence of Dinoflagellate Histone Proteins
Source: G3 (Bethesda). 2015 Dec 8;6(2):397–422. doi: 10.1534/g3.115.023275 (PMC4751559; doi:10.1534/g3.115.023275)
Supplement: Supporting Information [file supp_g3.115.023275_FigureS2.pdf]

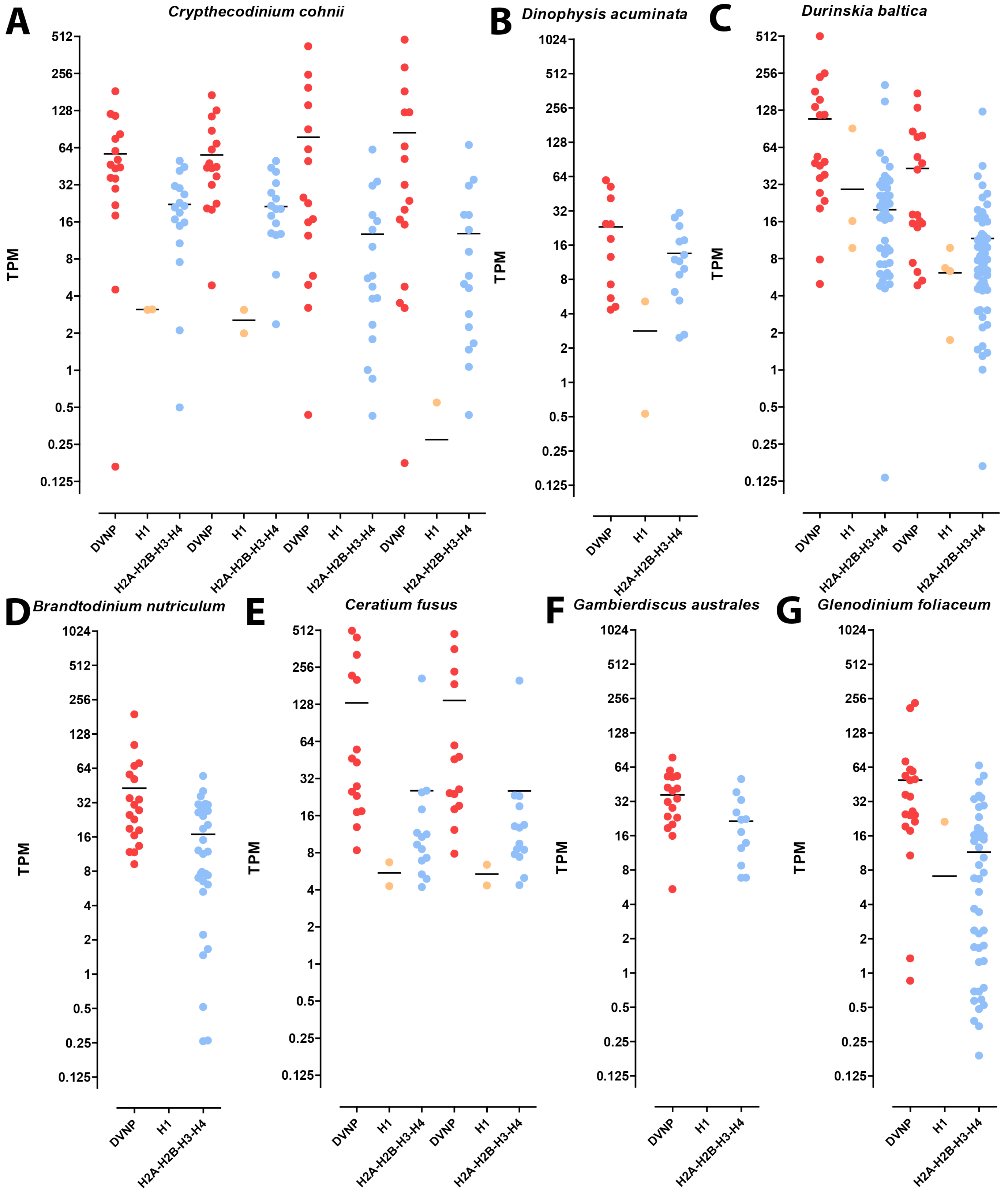

Figure S2: Expression levels of DVNP, linker histone and histone genes in dinoflagellates. (A) *Crypthecodinium cohnii*; from left to right: SRR1296889, SRR1296890, SRR1296960, SRR1296961; (B) *Dinophysis acuminata*; SRR1296701; (C) *Durinskia baltica*; from left to right: SRR1296839, SRR1296941; (D) *Brandtodinium nutriculum*: SRR1300537; (E) *Ceratium fusus*: from left to right: SRR1300300, SRR1300301; (F) *Gambierdiscus australes*: SRR1296893; (G) *Glenodinium foliaceum*: SRR1296842.
