# Supplementary material for: Diversity and Divergence of Dinoflagellate Histone Proteins
Source: G3 (Bethesda). 2015 Dec 8;6(2):397–422. doi: 10.1534/g3.115.023275 (PMC4751559; doi:10.1534/g3.115.023275)
Supplement: Supporting Information [file supp_g3.115.023275_FigureS3.pdf]

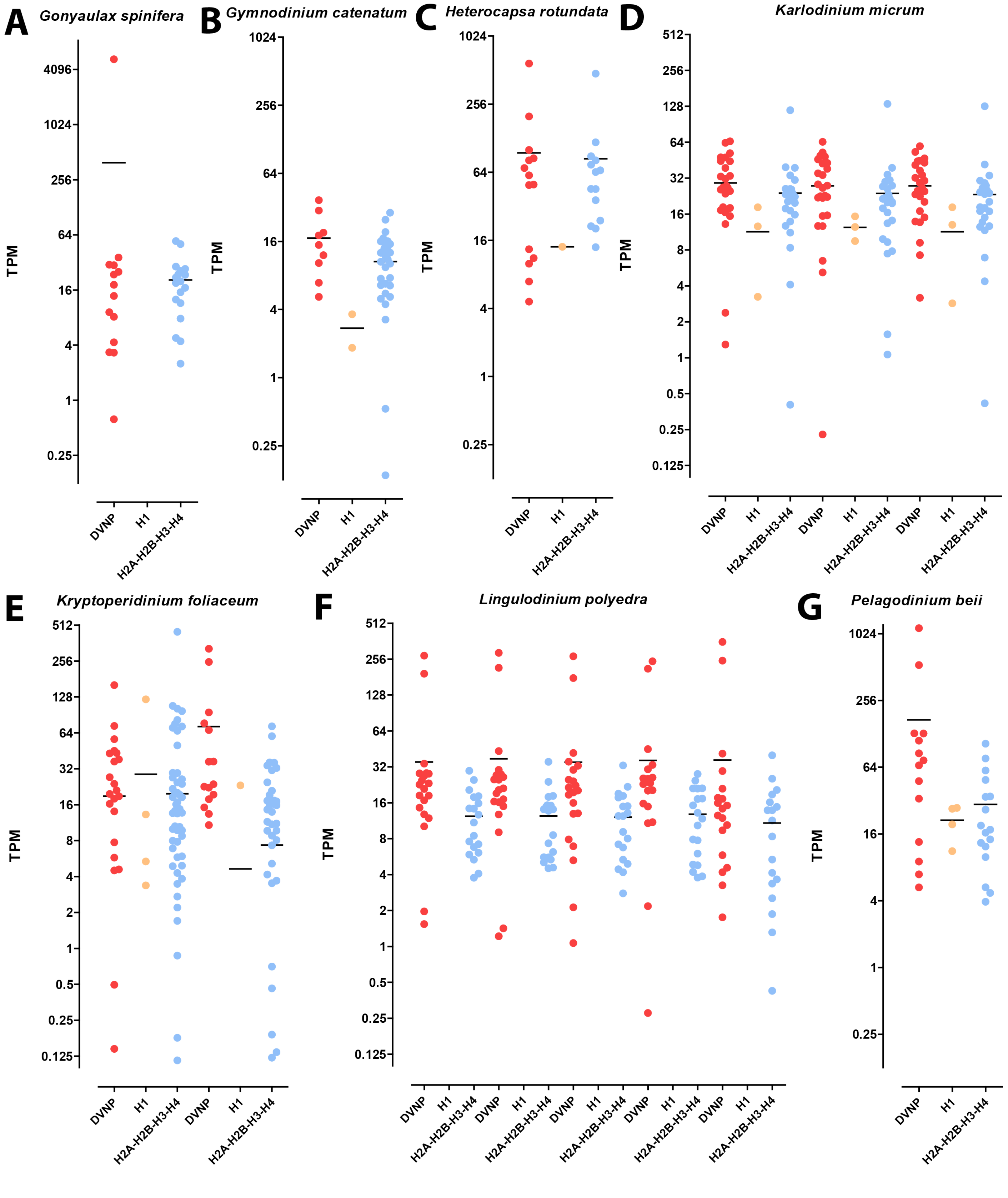

Figure S3: Expression levels of DVNP, linker histone and histone genes in dinoflagellates. (A) *Gonyaulax spinifera*: SRR1300518; (B) *Gymnodinium catenatum*: SRR1296705; (C) *Heterocapsa rotundata*: SRR1296810; (D) *Karlodinium micrum* CCMP2283; from left to right: SRR1300325, SRR1300326, SRR1300327; (E) *Kryptoperidinium foliaceum*; from left to right: SRR1296841, SRR1296842; (F) *Lingulodinium polyedra*; from left to right: SRR1300255, SRR1300256, SRR1300257, SRR1300258, SRR584359; (G) *Pelagodinium beii*: SRR1300503.
