# Supplementary material for: Diversity and Divergence of Dinoflagellate Histone Proteins
Source: G3 (Bethesda). 2015 Dec 8;6(2):397–422. doi: 10.1534/g3.115.023275 (PMC4751559; doi:10.1534/g3.115.023275)
Supplement: Supporting Information [file supp_g3.115.023275_FigureS4.pdf]

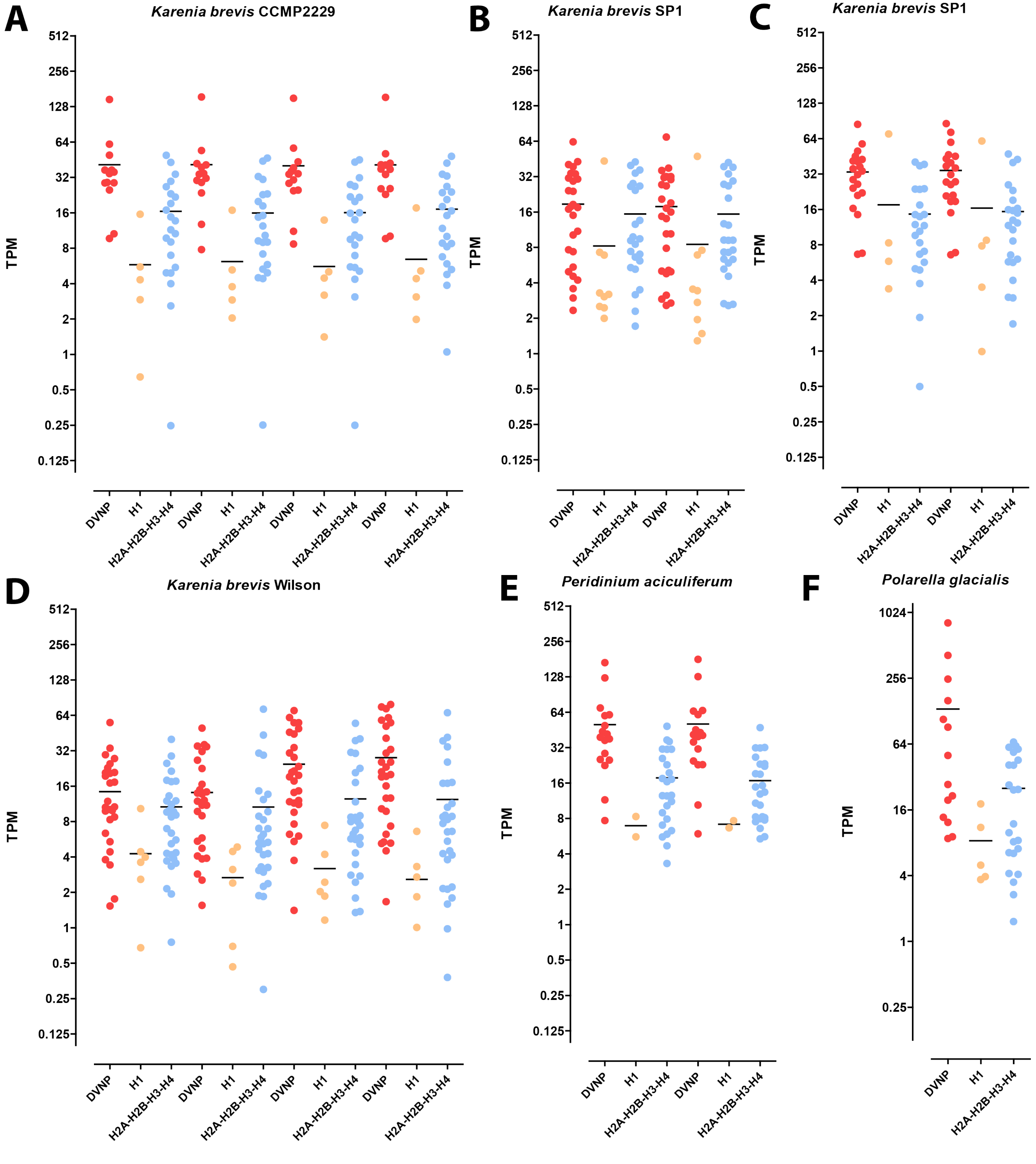

Figure S4: Expression levels of DVNP, linker histone and histone genes in dinoflagellates. (A) *Karenia brevis* CCMP2229; from left to right: SRR1296748, SRR1296749, SRR1296750, SRR1296952; (B) *Karenia brevis* SP1; from left to right: SRR1296712, SRR1296714; (C) *Karenia brevis* SP3; from left to right: SRR1163514, SRR1163516; (D) *Karenia brevis* Wilson; from left to right: SRR1296743, SRR1296744, SRR1296853, SRR1296854; (E) *Peridinium aciculiferum*; from left to right: SRR1294439, SRR1294440; (F) *Polarella glacialis*; SRR1296751.
