# Supplementary material for: Diversity and Divergence of Dinoflagellate Histone Proteins
Source: G3 (Bethesda). 2015 Dec 8;6(2):397–422. doi: 10.1534/g3.115.023275 (PMC4751559; doi:10.1534/g3.115.023275)
Supplement: Supporting Information [file supp_g3.115.023275_FigureS5.pdf]

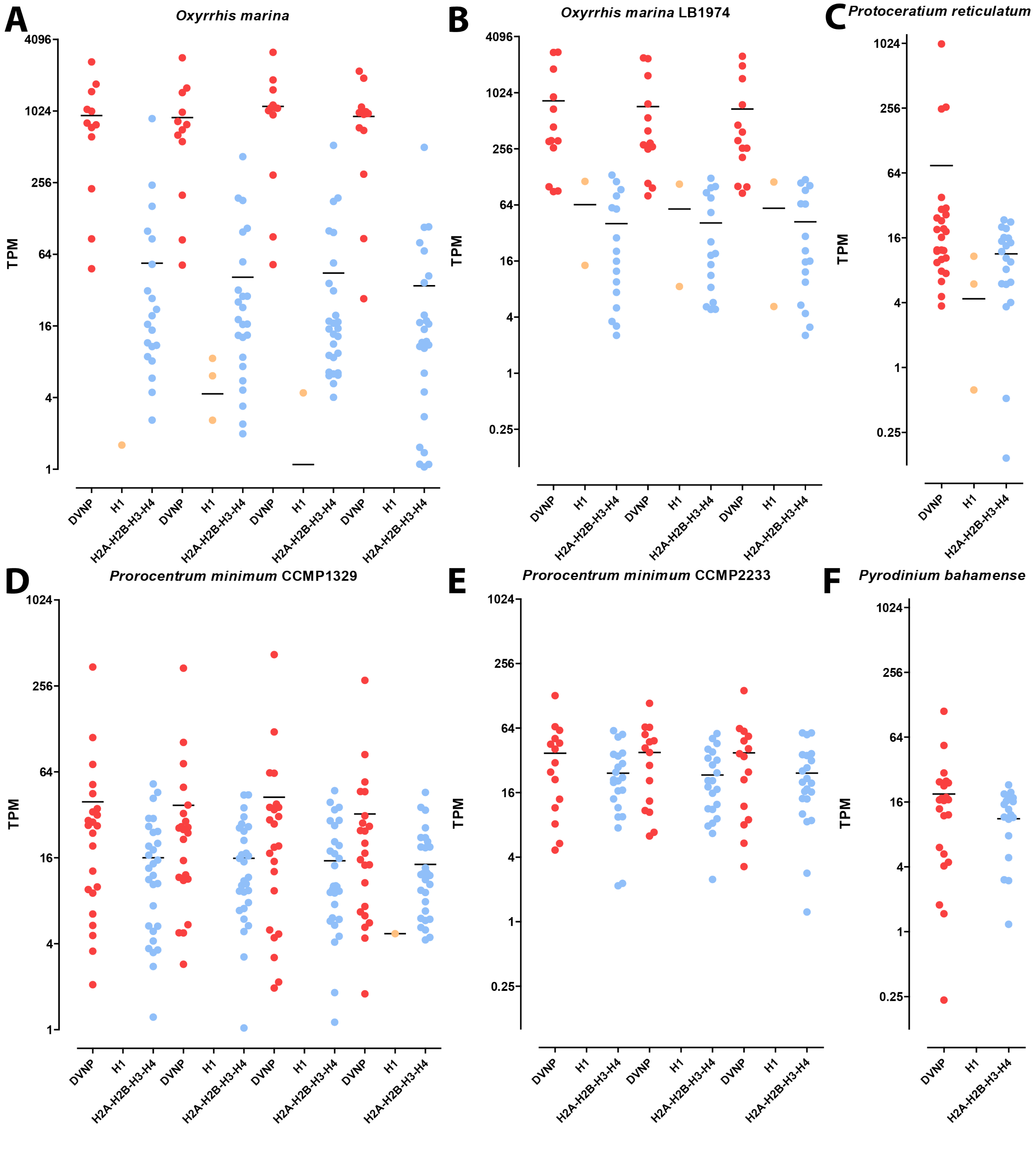

Figure S5: Expression levels of DVNP, linker histone and histone genes in dinoflagellates. (A) *Oxyrrhis marina*; from left to right: SRR1296900, SRR1296901, SRR1296903, SRR1296907; (B) *Oxyrrhis marina* LB1974; from left to right: SRR1300472, SRR1300473, SRR1300474; (C) *Protoceratium reticulatum*: SRR1296738; (D) *Prorocentrum minimum* CCMP1329; from left to right: SRR1296784, SRR1296785, SRR1296787, SRR1296788; (E) *Protoceratium reticulatum* CCMP2233; from left to right: SRR1296752, SRR1296753, SRR1296754; (F) *Pyrodinium bahamense*: SRR1296702.
