# Supplementary material for: Diversity and Divergence of Dinoflagellate Histone Proteins
Source: G3 (Bethesda). 2015 Dec 8;6(2):397–422. doi: 10.1534/g3.115.023275 (PMC4751559; doi:10.1534/g3.115.023275)
Supplement: Supporting Information [file supp_g3.115.023275_FigureS6.pdf]

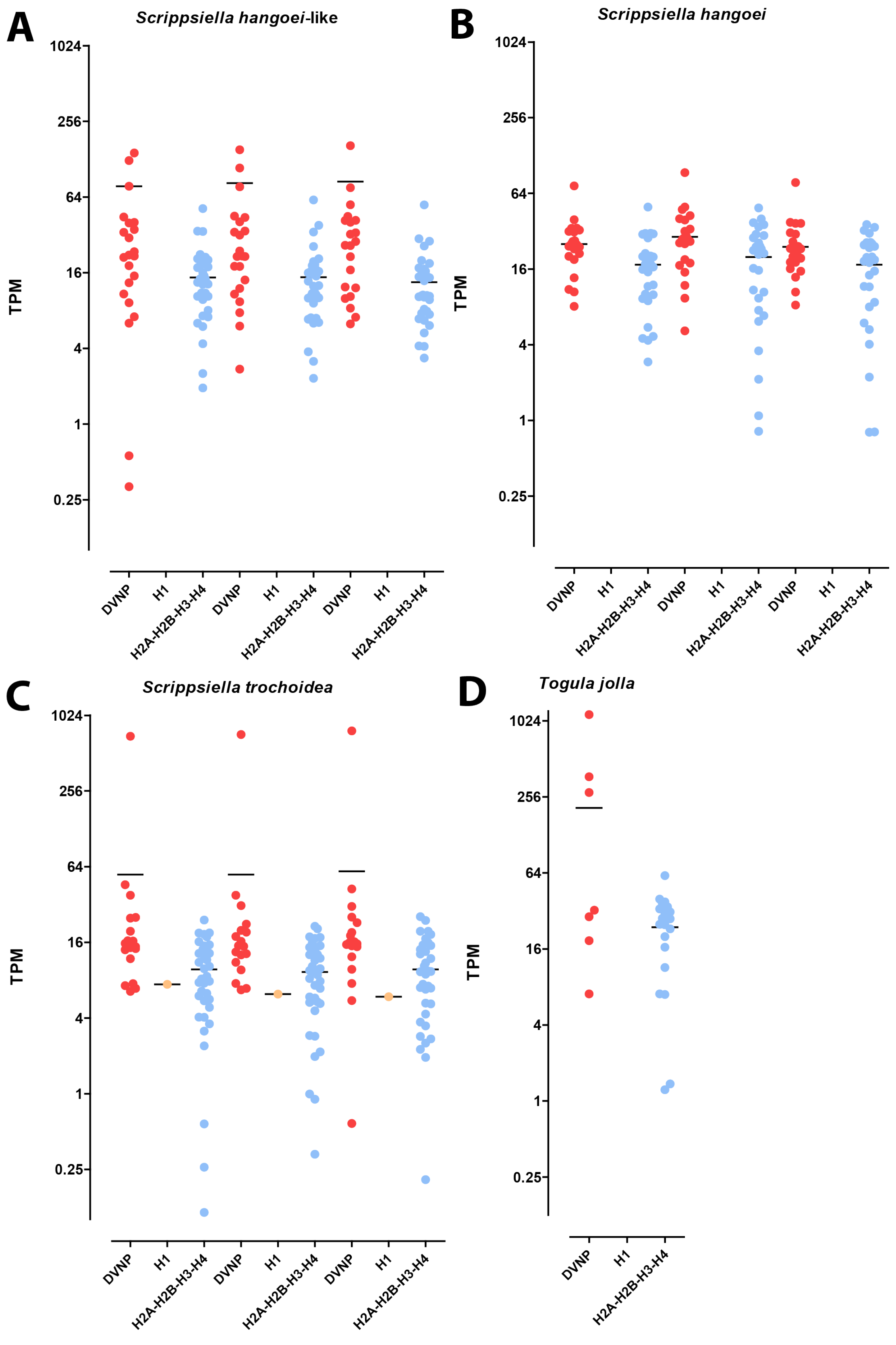

Figure S6: Expression levels of DVNP, linker histone and histone genes in dinoflagellates. (A) *Scrippsiella hangoei*-like; from left to right: SRR1296793, SRR1296794, SRR1296796; (B) *Scrippsiella hangoei*; from left to right: SRR1294400, SRR1296786, SRR1296972; (C) *Scrippsiella trochoidea*; for left to right: SRR1296759, SRR1296760, SRR1296761; (D) *Togula jolla*: SRR1296741.
