# Supplementary material for: Diversity and Divergence of Dinoflagellate Histone Proteins
Source: G3 (Bethesda). 2015 Dec 8;6(2):397–422. doi: 10.1534/g3.115.023275 (PMC4751559; doi:10.1534/g3.115.023275)
Supplement: Supporting Information [file supp_g3.115.023275_FigureS11.pdf]

```
CAMPEP_0200546290/1-167    1 MARTKQTARMSTGSKAPRQVLAAKTVRKQAPISGGVKKPHRYRPGTVA  48
CAMPEP_0200538292/1-136    1 MARTKQTARKSSAGKAPRVQLAAKAARKQSPVVGGIKKPHRYRAGTVA  48
Homo_sapiens/1-136         1 MARTKQTARKSTGGKAPRKQLATKAARKSAPATGGVKKPHRYRPGTVA  48
CAMPEP_0200540706/1-316    1 MARTKQTARKSTGGKAPRSQLAAKAARKQTPVAGGIKKPHRYRPGTVA  48

CAMPEP_0200546290/1-167   49 LREIRKYQKSTELLIRKLPFQRLVREIAQDFKTDLKFQSQAVLALQEA  96
CAMPEP_0200538292/1-136   49 LREIRKYQKSTDLLLRKLPFQRLVREIAQAFKSEVLFQSQAVLALQEA  96
Homo_sapiens/1-136        49 LREIRRYQKSTELLIRKLPFQRLVREIAQDFKTDLRFQSSAVMALQEA  96
CAMPEP_0200540706/1-316   49 LREIRKYQKSTDLLIRKLPFQRLVREIAQDFKTDLKFQSQAVLALQEA  96

CAMPEP_0200546290/1-167   97 AEAYLVGLFEDTNLCAIHAKRVTIMPKDVQLARRLRGERIEADGPLLL 144
CAMPEP_0200538292/1-136   97 AEAYLVGLFEDTNLCAIHGRRVTIMPKDMQLARRLRGERV-------- 136
Homo_sapiens/1-136        97 CEAYLVGLFEDTNLCAIHAKRVTIMPKDIQLARRIRGERA-------- 136
CAMPEP_0200540706/1-316   97 AEAYLVGLFEDTNLCAIHAKRVTIMPKDVQLARRLRGERIEAHDAFVF 144

CAMPEP_0200546290/1-167  145 PLPQASLACEETCNYVFLGGAVQ------------------------- 167
CAMPEP_0200538292/1-136      ------------------------------------------------
Homo_sapiens/1-136           ------------------------------------------------
CAMPEP_0200540706/1-316  145 LYKLVARAARRRVLPRQADDAWAAVNARSIASIPPIGAHAPAAGVQPV 192

CAMPEP_0200546290/1-167      ------------------------------------------------
CAMPEP_0200538292/1-136      ------------------------------------------------
Homo_sapiens/1-136           ------------------------------------------------
CAMPEP_0200540706/1-316  193 LPQPIPASCVWGGSPQMAEGYEEXSRPWQTASCARPPGGERAEHAVPS 240

CAMPEP_0200546290/1-167      ------------------------------------------------
CAMPEP_0200538292/1-136      ------------------------------------------------
Homo_sapiens/1-136           ------------------------------------------------
CAMPEP_0200540706/1-316  241 CARGAARRRRDGGEVAPVHALGPRGLDLLGSEAGSVGRVHGRLAFRDG 288

CAMPEP_0200546290/1-167      ----------------------------
CAMPEP_0200538292/1-136      ----------------------------
Homo_sapiens/1-136           ----------------------------
CAMPEP_0200540706/1-316  289 RALGAAVATRVMGRCGNSLPMVKSKKTX                     316
```

Figure S11: Multiple sequences alignments of histones H3 sequences from *Alexandrium monilatum* and histone H3.1 from *Homo sapiens*.. Sequences were aligned using MUSCLE (Edgar 2004) (version 3.8.31) and visualized using JalView (Waterhouse et al. 2009) (version 2.8.2).
