# Supplementary material for: Diversity and Divergence of Dinoflagellate Histone Proteins
Source: G3 (Bethesda). 2015 Dec 8;6(2):397–422. doi: 10.1534/g3.115.023275 (PMC4751559; doi:10.1534/g3.115.023275)
Supplement: Supporting Information [file supp_g3.115.023275_FigureS7.pdf]

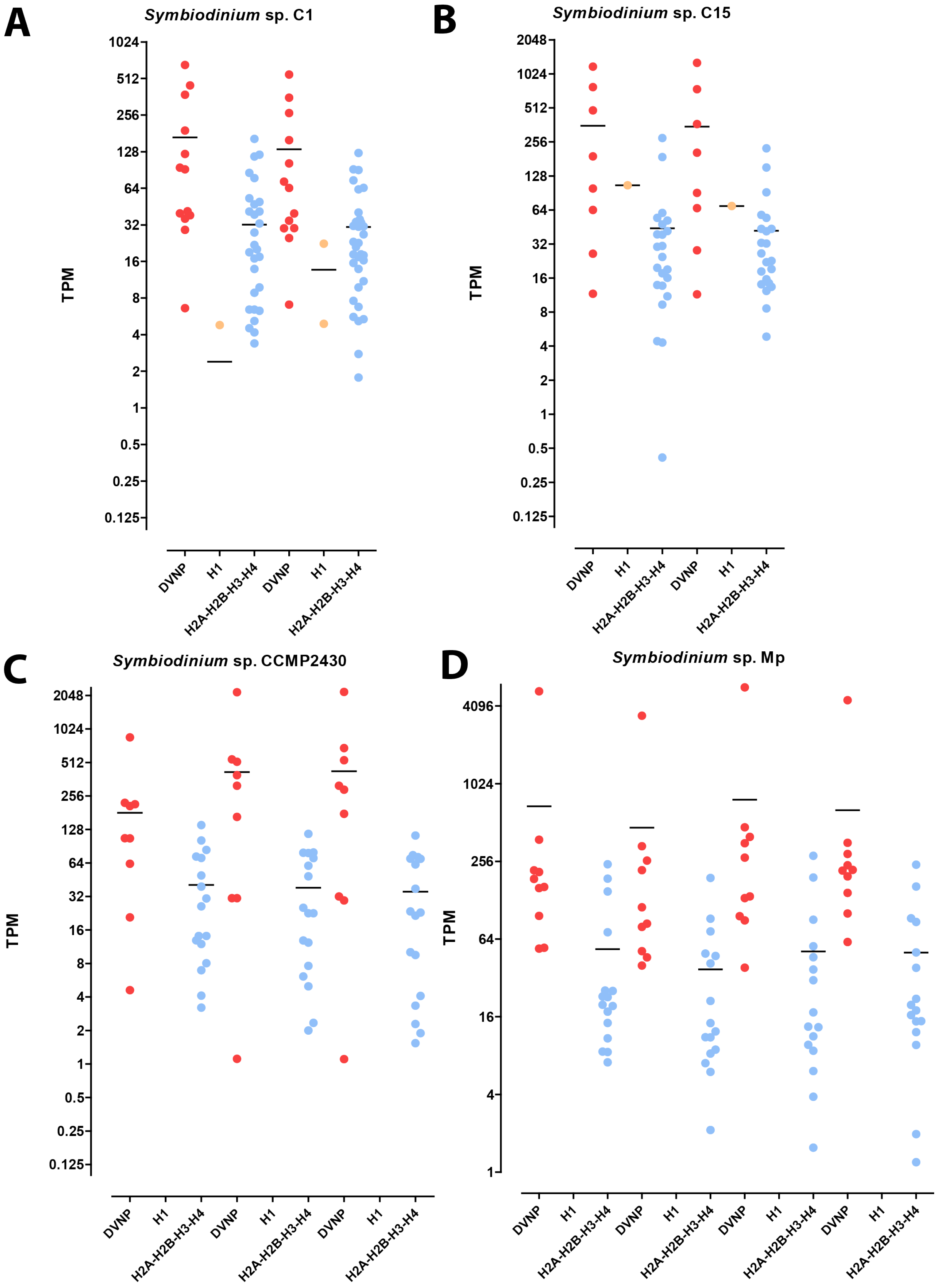

Figure S7: Expression levels of DVNP, linker histone and histone genes in dinoflagellates. (A) *Symbiodinium* sp. C1; from left to right: SRR1300430, SRR1300431; (B) *Symbiodinium* sp. C15; from left to right: SRR1300470, SRR1300471; (C) *Symbiodinium* sp. CCMP2430; for left to right: SRR1300264, SRR1300265, SRR1300266; (D) *Symbiodinium* sp. Mp; from left to right: SRR1300267, SRR1300343, SRR1300344, SRR1300345.
