# Supplementary material for: Diversity and Divergence of Dinoflagellate Histone Proteins
Source: G3 (Bethesda). 2015 Dec 8;6(2):397–422. doi: 10.1534/g3.115.023275 (PMC4751559; doi:10.1534/g3.115.023275)
Supplement: Supporting Information [file supp_g3.115.023275_FigureS8.pdf]

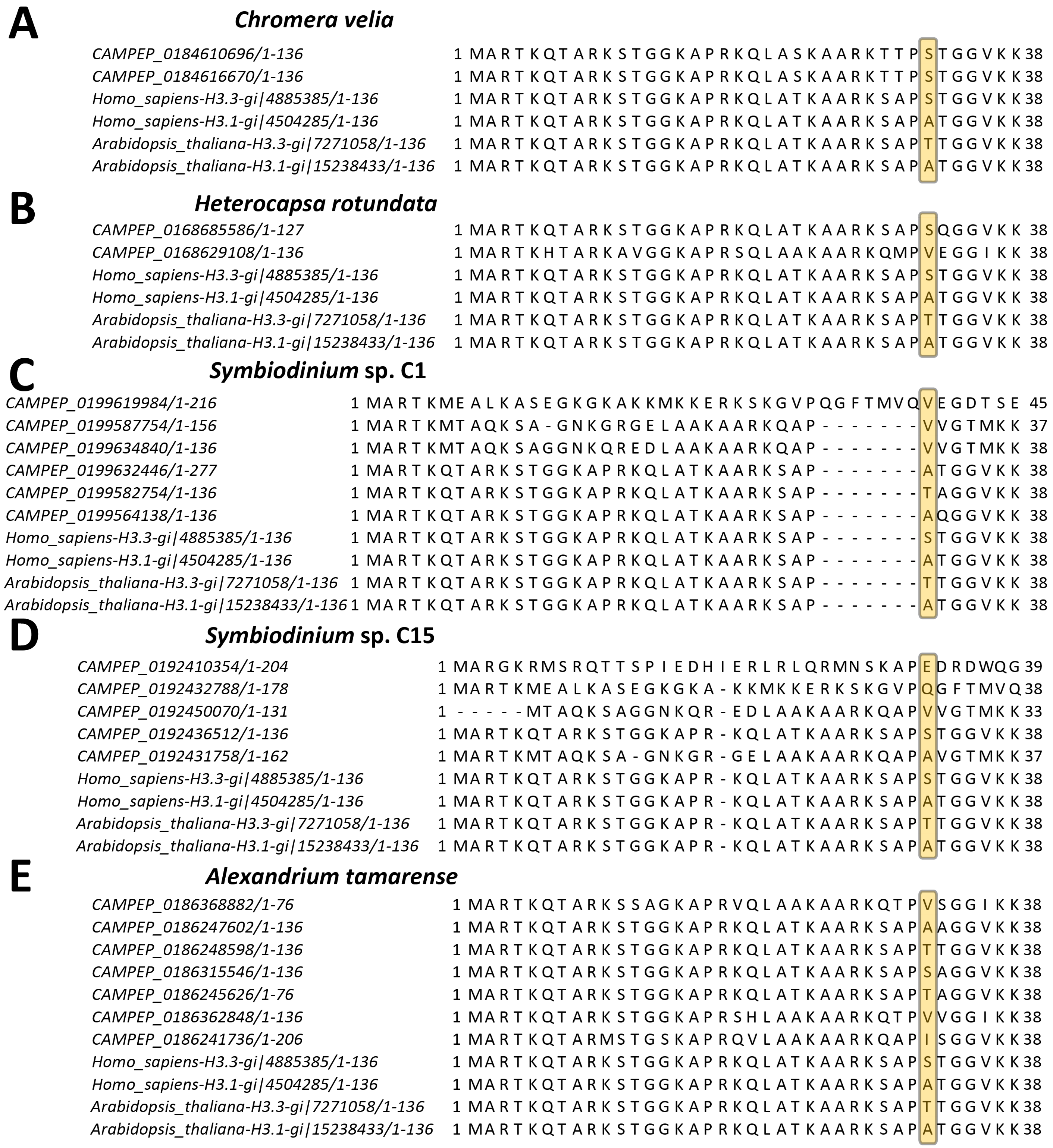

Figure S8: Putative H3.3/H3.1 histone variants in dinoflagellates. Histones H3.3 and H3.1 are distinguished by the sequence at position 31, which is S or T (in animals and fungi, respectively) in H3.3 and A in H3.1. Slightly different sequences are observed in other groups, for example in *Tetrahymena* the H3.3/H3.1 distinction is that the sequence is VS vs AT, respectively (Talbert et al. 2012). Putative H3.3/H3.1 pairs ar observed in several dinoflagellate transcriptomes: *Heterocapsa rotundata* (B), *Symbiodinium* sp. C1 (C), *Symbiodinium* sp. C15 (D), and *Alexandrium tamarense* (E). Only H3.3 variants are observed in the *Chromera velia* transcriptome, thus caution with respect to false negatives have to be exercised when interpreting the presence/absence of H3.3/H3.1 variants.
